# Breath-giving cooperation: critical review of origin of mitochondria hypotheses: Major unanswered questions point to the importance of early ecology

**DOI:** 10.1186/s13062-017-0190-5

**Published:** 2017-08-14

**Authors:** István Zachar, Eörs Szathmáry

**Affiliations:** 1 Eötvös Loránd University, Department of Plant Systematics, Ecology and Theoretical Biology, Pázmány P. sétány 1/C, Budapest, 1117 Hungary; 2 Evolutionary Systems Research Group, MTA, Centre for Ecological Research, Hungarian Academy of Sciences, Klebelsberg Kunó str. 3., Tihany, 8237 Hungary; 3 Parmenides Foundation, Kirchplatz 1, 82049 Pullach/Munich, Munich, Germany

**Keywords:** Mitochondria, Endosymbiosis, Eukaryogenesis, Ecology, Evolution, Metabolism, Parasitism, Major transition

## Abstract

The origin of mitochondria is a unique and hard evolutionary problem, embedded within the origin of eukaryotes. The puzzle is challenging due to the egalitarian nature of the transition where lower-level units took over energy metabolism. Contending theories widely disagree on ancestral partners, initial conditions and unfolding of events. There are many open questions but there is no comparative examination of hypotheses. We have specified twelve questions about the observable facts and hidden processes leading to the establishment of the endosymbiont that a valid hypothesis must address. We have objectively compared contending hypotheses under these questions to find the most plausible course of events and to draw insight on missing pieces of the puzzle. Since endosymbiosis borders evolution and ecology, and since a realistic theory has to comply with both domains’ constraints, the conclusion is that the most important aspect to clarify is the initial ecological relationship of partners. Metabolic benefits are largely irrelevant at this initial phase, where ecological costs could be more disruptive. There is no single theory capable of answering all questions indicating a severe lack of ecological considerations. A new theory, compliant with recent phylogenomic results, should adhere to these criteria.

Reviewers: This article was reviewed by Michael W. Gray, William F. Martin and Purificación López-García.

**Electronic supplementary material:**

The online version of this article (doi:10.1186/s13062-017-0190-5) contains supplementary material, which is available to authorized users.

## Background

The origin of eukaryotes was a major evolutionary transition and a hard problem of evolution [1–3]. The endosymbiotic origin of mitochondria is closely coupled to eukaryogenesis [4], in fact, so closely, that no primarily amitochondriate eukaryotes are known [5, 6]. Whether eukaryotes were evolved directly because of mitochondria or the mitochondrion was the last step in the process is heavily debated [4, 7, 8], but eukaryogenesis was certainly closely coupled to endosymbiosis. Unfortunately, the transition is not documented very clearly either by fossils or by the genome: there are no known intermediates bridging the gap between modern eukaryotes and prokaryotes. Eukaryotes, as the name tells it, were classified so because they possess a true nucleus (compacted chromosomes bound by a porous double membrane within the cytoplasm). Since all amitochondriate eukaryotes at present are secondarily derived, it’s true that just as the nucleus is a *conditio* sine qua non of Eukarya, so are mitochondria [9].

More than twenty different endosymbiotic theories were presented for the origin of the eukaryotes [10–12]. They all agree in that endosymbiosis was important, perhaps even crucial in eukaryogenesis [4, 13, 14] though there is no agreement in the nature of the host or the endosymbiont, and in the order of events, which are hidden behind the event horizon of the last eukaryotic common ancestor (LECA) [15]. The two major arguments assume that the LECA was either complex compared to contemporary prokaryotes (having most eukaryotic inventions) and mitochondria came late (e.g. [7, 16–19]), or LECA was barely more evolved than an archaeon and the early acquisition of mitochondria triggered the subsequent evolutionary innovations (e.g. [12, 13, 20]). However, the devil is in the details, and it is not constructive to sort theories into just two broad categories. There are strong arguments for and against all competing theories, but there is no comprehensive analysis on what a valid theory should account for. This paper aims to 1) provide a set of objective criteria that any theory should meet, 2) evaluate existing scenarios along these criteria, to 3) find out which theory of mitochondrial emergence provides the best explanation and 4) find the points where none deliver.

A modern mitochondrion produces ATP that is pumped out to the host’s cytosol (see Additional file 1: Figure S1). However, the microbial partners were not like that when they first met and formed the first eukaryotic common ancestor (FECA). On the other hand, from phylogenomic data, it is known that LECA was an already complex, fully eukaryotic cell: it possessed most eukaryotic hallmarks (actin-based cytoskeleton, complex endomembrane system, nuclear envelope, vesicle-trafficking, Golgi apparatus, lysosomes, autophagosomes, etc.) and mitochondria [4, 21, 22]. It was most likely a phagocytotic heterotroph with mitochondrial aerobic respiration. But what it was like *before* acquiring its symbiont?

Interestingly, most theories assume that the relationship of host and symbiont was already mutualistic when integration started, though there is some indication that this was not the case [23]. The acquisition of an alphaproteobacterium [7, 9, 15] must have provided a conflict of interest between the partners forced to coexist, as it is the case with all egalitarian major transitions [3]. The endosymbiont could exploit the lack of regulated control by reproducing at the expense of the host; or the host could simply digest away its partner. Ignoring early conflict and expecting that the partnership appeared in its present mutually beneficial form is fallacious. It assumes that the very same conditions applied at the time of the merger that apply now and that the transition from independent to dependent relationship (with nuclear transfer) was instantaneous, making initial problems momentary. Both are unlikely. Energy production, metabolic compartmentation, division of labor and genome integration cannot be the condition but the conclusion of the merger.

In this paper we focus on early mitochondrial evolution and only tangentially discuss other aspects of eukaryogenesis, e.g. the origin of the nucleus. First, we present what we *know* about the phylogenetic position of the assumed partners. Next, we define two sets of questions, one for investigating observable facts of mitochondria and eukaryotic cells, the other to inquire about presumed historical processes leading to the present relationship. Hypotheses are comparatively evaluated and discussed within the frame of these questions in the Additional file 1: S3 and S4, in the manner similar to the review of Számadó and Szathmáry about another major transition: human language [24]. Our paper intends to find out if there is a theory able to consistently account for all the observed facts and can also provide a reasonable, consistent scenario for the unknown. To make it harder, the theory has to fit the phylogenetic data. A conclusion sums up the insight drawn from the analysis: no single theory is capable of answering all questions and even those scoring more have heavy shortcomings or debatable assumptions. The analysis indicates that more emphasis has to be put on early ecology (both biotic and abiotic factors) instead of metabolism, especially to scenarios which did not start mutually beneficial.

## Results

There is a wide variety of taxa postulated as initial host and symbiont, but in the last decade, phylogenetic results strongly support an archaeal host from the TACK superphylum [17, 25–28] and an alphaproteobacterial ancestor of the guest [9, 29, 30] (for more details on possible hosts and symbionts, see Additional file 1: S1 and Table S1). The LECA was already mitochondriate and all mitochondria-related organelles (MRO: anaerobic mitochondria, mitosomes, hydrogenosomes) are monophyletic [31, 32] and any loss of mitochondria is secondary and polyphyletic [5, 6, 33].

**Fig. 1:**
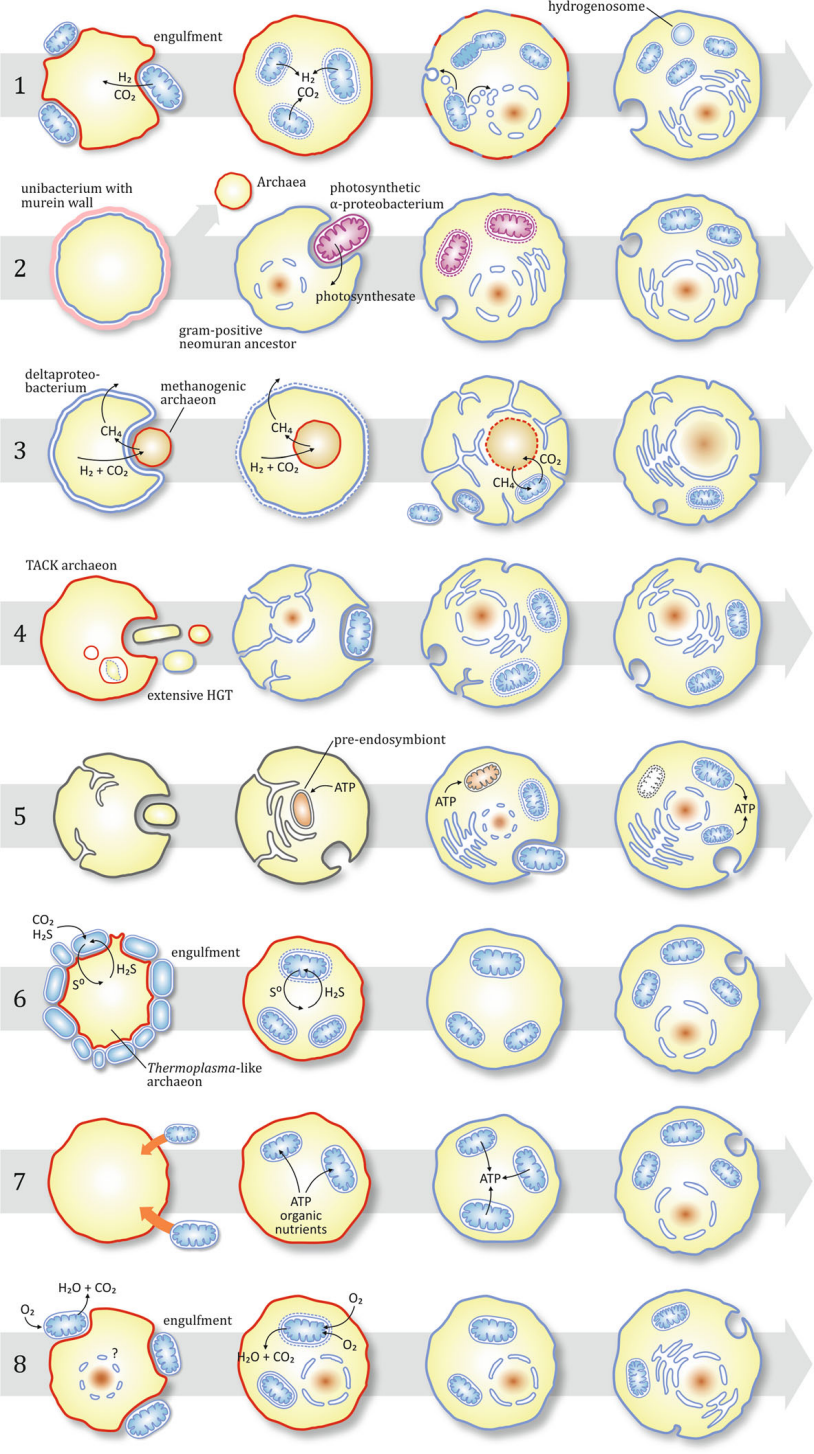
Scenarios of the various mitochondrial origin models. Scenarios focus mostly on topological changes, after the works of Martin and others [12, 31, 57, 68, 109]. Archaea are depicted with *red* membrane, Bacteria with *blue*; *purple* indicates photosynthetic ability. *Dashed curves* stand for degrading membranes. If not indicated syntrophic “engulfment”, the inclusion involved phagocytosis (even if primitive) with at least a rudimentary cytoskeleton (indicated by the host forming phagosomal inclusions). If not indicated otherwise, mitochondria perform aerobic respiration. Ultimately, in all scenarios, mitochondria implement metabolic compartmentation and produce ATP. 1) Hydrogen hypothesis [12, 45, 67]. 2) Photosynthetic symbiont theory [36, 37, 74]. 3) Syntrophy hypothesis [48, 110]. 4) Phagocytosing archaeon theory [16]. 5) Pre-endosymbiont hypothesis [9, 41]. The origin of the endomembrane system (and nucleus) is not specified explicitly, but one must assume that it evolved endogenously, the pre-endosymbiont (brown organelle) being related to the internal membrane system. 6) Sulfur-cycling hypothesis [46, 57, 111]. 7) Origin-by-infection hypothesis [57]. 8) Oxygen-detoxification hypothesis [68, 69, 103]. The presence of a forming nucleus at the start is unknown [68]

As a result of the huge list of potential partners suggested, there are infinitely many ways to combine a host with a symbiont and to split the theory space of eukaryotic and particularly mitochondrial origins. Consequently, there is a huge number of hypotheses [12]. To restrict the scope, we ignored hypotheses focusing on the origin of the nucleus or other eukaryotic features; also ignored those being reasonably refuted and are generally not accepted (e.g. the archezoa hypothesis [34] or the PTV scenario [35]). Other models of interest were left out purely to limit the size of the text. The selected eight scenarios are depicted in Fig. 1 with a brief description of each hypothesis in Additional file 1: S2.

Common divisions of theory space are whether the host was an archaeon, a bacterium or a primitive eukaryote, whether the nucleus was endogenously or exogenously derived, whether phagocytosis came before mitochondria, and what the initial metabolic relationship was. Table 1 provides a classification along two broad dimensions (for a more detailed classification with more hypotheses included, see Additional file 1: Table S1). Based on mitochondria alone, the main schism is the order of events and whether mitochondria came early or late [16]:

- **Phagocytosis early, mitochondria late**. Eukaryotes gradually evolved from a lineage without mitochondria (either the once postulated eukaryotic Archezoa [34], Neomura [36, 37], other bacteria [35, 38] or Archaea [16]) and eventually acquired mitochondria via the only mechanistically plausible way: phagocytosis. Hence mitochondria could not trigger eukaryogenesis, coming quite late to the party. Amitochondriate eukaryotes or almost-eukaryotic prokaryotes could still exist, though not found yet. Suggested early (by e.g. [39, 40]) and lately again due to supporting proteomic and phylogenomic data [6, 7, 16–19, 41–43].
- **Mitochondria early, phagocytosis late**. The eukaryotic lineage emerged from a symbiosis between a non-phagocytotic host and the mitochondrial ancestor. Ultimately, this symbiogenesis [42] triggered the subsequent evolution of typical eukaryotic features and possibly the nucleus. If the host is assumed to be an archaeon then the origin of eukaryotes was initiated by a fusion between Archaea and Bacteria [35]. Phagocytosis only became feasibly later, perhaps due to the energy provided by the mitochondria [13]. Amitochondriate eukaryotes are primarily missing, Archezoa never existed [44]. Mitochondriate prokaryotes could still exist, but not found yet. Syntrophic theories belong here, either assuming an archaeal [45–47] or a bacterial host [48]. The early appearance of mitochondria also has some phylogenetic support [49].

**Table 1:** Possible combinations of components and scenarios discussed in this paper

	Host (cytoplasm and possible nucleus)			
		Primitive eukaryote	Archaeon	Bacterium
Ecological relationship (inclusion mechanism)	syntrophy (+ / +) (engulfment)	• ox-tox model [68, 69]	• hydrogen hypothesis [45] • sulfur-cycling hypothesis [57]	• syntrophy hypothesis (+archaeon as nucleus) [48]
	predation (+ / -) (phagocytosis)	• pre-endosymbiont hypothesis [9, 41]	• phagocytosing archaeon theory [16]	• photosynthetic symbiont theory [36]
	parasitism (- / +) (invasion)	•	• origin-by-infection hypothesis [57]	•

The + and - signs in the second column indicate ecologically beneficial (+) or costly (−) interactions for the host/guest. For a more detailed view and more hypotheses included, see Additional file 1: Table S1

Such limited classifications however are not immensely useful as they blur important differences. One should rather ask more questions to investigate the case in detail (for further criteria, see [15]). Hereby we provide an extended inquisitive frame by asking twelve specific questions that any reasonable hypothesis of mitochondrial origin must answer. We restrict questions particularly relating to the origin of mitochondria but within the unavoidable context of eukaryogenesis.

Six questions point to readily observable facts about partners and the result of the merger. These questions are discussed in detail in section Observables and results of the comparative evaluation of the eight hypotheses are provided in Additional file 1: S3 and Table S2:

1. unique, singular origin of eukaryotes and mitochondria;
2. lack of intermediate, transitional forms;
3. chimaeric nature of eukaryotes, especially membranes;
4. lack of membrane bioenergetics in the host;
5. lack of photosynthesis in symbiont;
6. origin and present phylogenetic distribution of MROs.

Six questions investigate the historical events that cannot be observed anymore [41] and we can only guess about. We’ve introduced a rather important aspect that is often neglected though has a profound impact on the unfolding of events: the initial ecological relationship, predating the establishment of the ATP transport between host and symbiont. Accordingly, the unknowns are (discussed in section Historicals, results listed in Additional file 1: Table S3 and detailed in Additional file 1: S4):

7) the original metabolism of host;
8) the original metabolism of symbiont;
9) the initial ecological relationship of the partners that specified the initial conditions and restrictions of the merger, and what stabilized this relationship;
10) the early selective advantage of the partnership;
11) the mechanism of inclusion;
12) the mechanism of vertical transmission of the proto-endosymbionts.

While “ecology” should include abiotic factors as well, here we only focus on the biotic aspect (relationship of partners), ignoring the environmental conditions, as that would take up another review of its own. Nevertheless, it must be emphasized that abiotic factors are as important as biotic ones in defining the selective forces in the course of evolution.

Strictly speaking, any hypothesis should account for all the steps of obligate endosymbiosis, including (not necessarily in this order) the followings: initial benefit of the partners to maintain a stable relationship, metabolic or ecological, that could lead to long-term dependency; avoiding digestion or other defensive measures of the host; vertical transmission of the partnership; loss of photosynthesis (if any) in the symbiont; degradation of the phagosomal membrane (if there was one); insertion of nucleotide exporters and protein importers into the mitochondrial inner membrane; establishing the mitochondria as the main energy provider; relinquishing the host’s bioenergetic membranes; genetic transfer between nucleus and mitochondria; regulated, synchronized division of host and symbiont; uniparental inheritance. These twelve questions are discussed in turn.

## Discussion

### Observables

#### Eukaryotic singularity

The most enigmatic aspect of mitochondria is that they are closely and singularly bound with the origin of eukaryotes. All eukaryotes primarily have mitochondria or MROs and there are no known second origin of mitochondria nor eukaryotes. If mitochondria provided such an enormous opportunity, why in 4.5 billion years did eukaryotes only evolve once and why we don’t see similarly complex sister groups to Eukarya? Either there was an ecological, environmental or energetic barrier for eukaryogenesis that was hard to cross, or becoming eukaryote was easy but all parallel trials failed for some reason (for discussion, see Additional file 1: S3). While it is possible that new karyotic lineages could not invade already occupied eukaryotic or “archezoan” niches, we should at least see their phylogenetic signal (which we don’t).

**Fig. 2:**
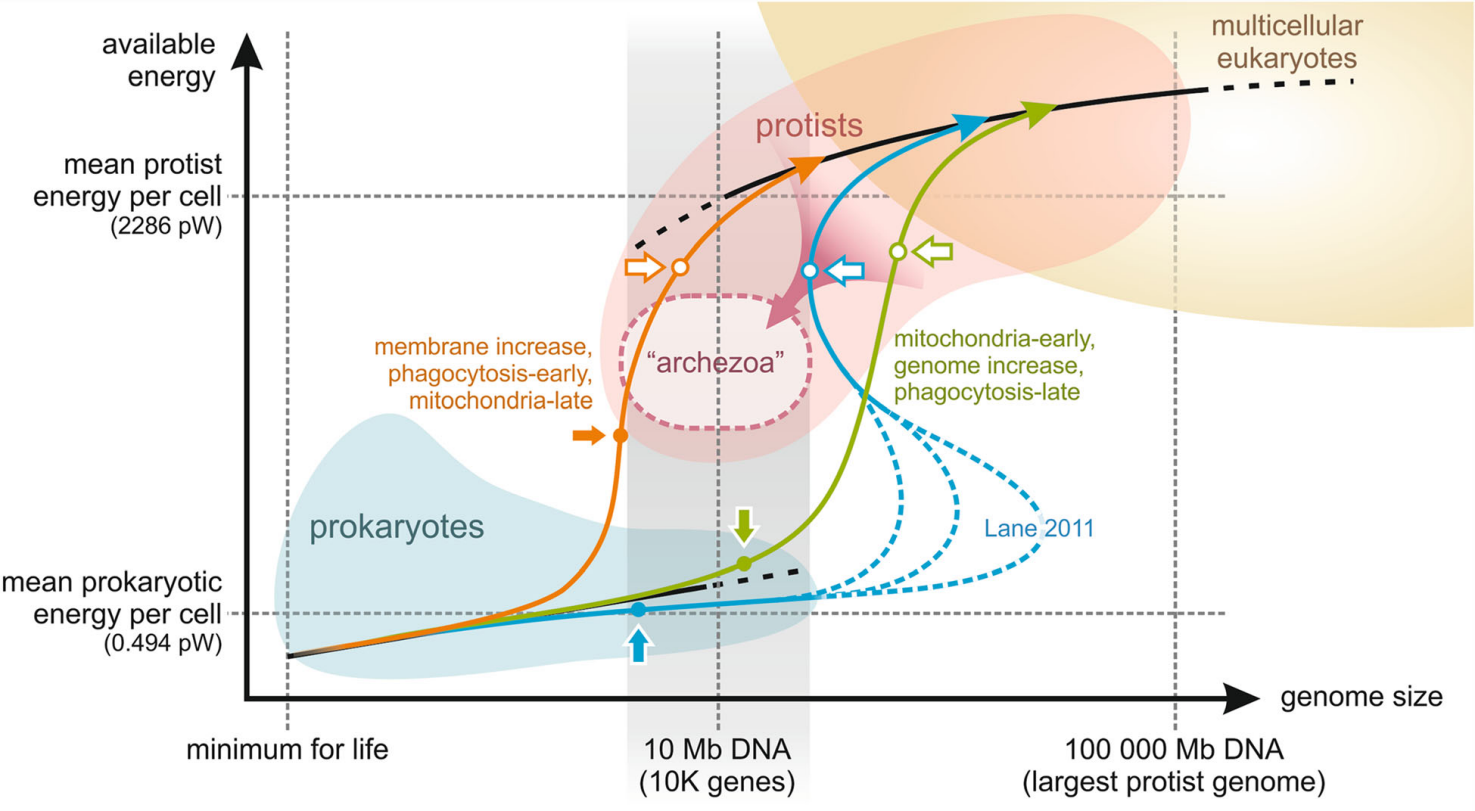
Energetic scenarios for the origin of eukaryotes. Filled arrows indicate FECA and the acquisition of mitochondria, empty arrows stand for LECA. Black lines roughly indicate averages in prokaryotes and eukaryotes. Prokaryotes cannot have genomes much larger than ~10 Mb (or ~10 K genes); smallest unicellular eukaryotes overlap with prokaryotes at this complexity. According to Lane and Martin [13, 50], there is an energetic barrier that prevents prokaryotes to maintain larger genomes (energy per cell values are from [13]). They claim that the early acquisition of mitochondria permit the transition of this barrier by temporarily increasing the gene count (blue curve; though the multiplier factor is only guessed by Lane, hence the dashed curves) to be able to experiment with new gene families. They maintain that amitochondriate eukaryotes cannot evolve directly from prokaryotes, only by losing the endosymbiont. Another possible scenario is to increase the area of internal respiratory membranes which provides extra energy with no additional genes (*orange curve*). This might just have been enough to power primitive phagocytosis. Mitochondria had to be acquired at a point where respiratory membranes could not be further exploited. Early mitochondria might induce gradual genome increase that progressively made inventions possible (*green curve*), though if this happened at low energetic levels, the archezoan niche (*dashed oval*) again could only be reached reductively. Theoretically, any trajectory between the orange and green curves is possible, either with early or late mitochondria. Ultimately, all scenarios lead to the same LECA, though starting from different FECAs. Present amitochondriate eukaryotes are secondarily derived (*purple arrow*), but some scenarios allow (*orange and dark green*) the existence of primarily amitochondriate “archezoan” eukaryotes

Lane and Martin argues that mitochondria released an energetic constraint and allowed an increase both in cell and genome size [13, 50, 51]. However, the fact that there are (secondarily reduced) amitochondriate eukaryotes in the archezoan niche barely having more genes than large prokaryotes (~10 K) indicates that for example phagocytosis is feasible without mitochondria. It is very likely that mitochondria-provided energy was indispensable for ultimate genome expansion but Lane’s claim of a 10-fold intermediate genome increase to experiment with new genes is unlikely, for at least two reasons. Firstly, it goes against the concept of gradual evolution of complex traits, as was pointed out by [3], denying the possibility of stepwise accumulation of adaptations. Even a small increase in genome size provides a huge increase in exploration space. While Lane expects an unlikely sequence of events, Booth and Doolittle point out that all evolutionary stories are “genuinely unlikely” [52] – especially applicable to eukaryogenesis, being a singular event in life history. Secondly, a ten-fold increase in a reasonable FECA genome would lead to increased rate of replicative errors, making such large proto-eukaryotic genomes unmanageable without sophisticated error-correcting mechanisms, lacking at that time [53] (the argument is further detailed in Additional file 1: S5). Furthermore, the claim that larger cell and genome sizes can only be achieved by surplus energy seems to dissolve when the relative cost of an added gene is considered within the evolutionary context of longer division times and smaller effective population size [54]. Accordingly, it is unlikely, that a huge genome increase was sudden and solely dependent on mitochondrial power (see Fig. 2 for a visual understanding). A more detailed quantitative study suggests that prokaryotes have available energy budgets in the same ballpark as eukaryotes have [55]. One also has to consider that if mitochondria were supposed to provide an energy surplus, predation (by phagocytosis) could have provided the very same boost. Parasitic symbiont theories [56, 57], by assuming that the parasite reduces the host’s fitness, provide a natural barrier preventing frequent symbiogenesis. There had to be a unique context though, for example a fluctuating environment, where the host-parasite pair had the advantage over independent individuals.

#### Absent intermediates

All extant eukaryotes share the signature eukaryotic features (endomembrane system, nuclear envelope, organelles, sex and syngamy), most importantly mitochondria, but these features seemingly appeared simultaneously as there are no intermediate transitional forms, only a glaring lack of apparent graduality. The genetic code is similarly unique, without intermediates or parallel solutions, however, the causes might not be the same (see Additional file 1: S3). It is reasonable to assume that some exclusively eukaryotic features represent the ancestral state. It is inevitable to accept the once presence of proto-eukaryotes with various combinations of inventions gradually acquired (mitochondria included). It is undeniable that all scenarios, either mitochondria-early or mitochondria-late [16, 29], are missing transitional forms. Fossil traces of intermediates, or even eukaryotes, from the Proterozoic are far from being convincing (see S6).

Either all eukaryotic inventions were rolled out in an extreme short time or there was strong selection in a prolonged time that ensured no intermediates survived. Assuming that the first is unlikely [58], either all intermediates disappeared or they have not been found yet. There are many features thought to be exclusive to Eukarya that have been found in prokaryotes [43, 59], though these are not part of a gradual evolutionary trajectory from simple to complex. Nevertheless, contrary to Lane and Martin, many phylogenetic analyses [7, 17, 21] support the theory that the archaeal host already had cytoskeletal features and possibly even primitive phagocytosis, strongly suggesting that mitochondria were added late, perhaps even lastly [7, 16, 18, 19] (other studies suggest mitochondria did not appear late [6, 49]) with the gradual acquisition of core eukaryotic traits. Whether phylogenetic reconstructions are right or not, it is a fact that many eukaryotic signature proteins [60] thought to be exclusive have homologs in Archaea, most prominently membrane remodelling and cytoskeletal proteins. While we do not necessarily know their functions in Archaea, their presence suggest the (once had) “ability to bend membranes and to form and transport internal vesicles, albeit at a much more primitive level than observed in modern eukaryotes” [61] which could have allowed primitive phagocytosis early on.

#### Chimaeric eukaryotes

Eukaryotes are genetic mosaics, with a more archaea-related information processing and intracellular organization machinery (e.g. ribosomes, histones [62], crenactin [59], tubulin homologs [43], ubiquitin-like system [63], membrane remodelling [64], etc.) and bacteria-related energy-metabolism [58, 65] and membranes. Archaeal genes in eukaryotes have a strong relation to the Archaea, stronger than to any bacteria.

The crucial difference between hypotheses is whether the archaeal component of the genome is vertically or horizontally acquired. Bacterial host models (see Table 1 and Additional file 1: Table S1) while inheriting the heterotrophic lifestyle or at least some enzymes from the host vertically, are not supported by the tight phylogenomic match of archaeal genes in eukaryotes to Asgard [61] or TACKL archaea [17], as then archaeal genes had to be acquired from archaea via HGT, which would result in a more diverse collection of archaeal genes. On the other hand, it seems equally inplausible that the host being an archaeon replaced its archaeal membrane entirely with bacterial membrane but retained most of its informational genes. The membrane-discrepancy of archaea and eukaryotes seriously impacts all hypotheses postulating an archaeal host (or those that derive archaea from bacteria [37]). There is no clear selective advantage of any membrane over the other in non-extreme habitats, which questions the motive of any scenario relying on replacement. It must be emphasized that even if replacement happened during eukaryogenesis there is no known other case in life’s history where membranes were replaced (in any direction; see [66]), not even locally for an organelle, neither are there known cases of symbiont-induced conversions (as postulated by [67]). Membrane replacement is something that could be tested experimentally.

#### Lack of membrane bioenergetics

All prokaryotes rely on their plasma membrane to generate energy. The host clearly lost all components of membrane bioenergetics and ATP synthesis from its plasma membrane. Did the host primarily lack these due to already being heterotrophic [16, 41, 48, 68, 69], or it was autotrophic (performing photosynthesis and/or respiration) and lost its electron transport chain (ETC) and phosphorylation ability due to acquiring mitochondria [12]? There could have been endogenous organelles that undertook bioenergetics before mitochondria, e.g. an endomembrane system [16, 70].

If the host already internalized respiration before mitochondria, there should be clues left that could explain how membrane energetics were lost. If endomembranes turned out to predate mitochondria (see [34, 71]), it would be a strong indication that they evolved primarily to increase absorption or/and energetic surface area. Either way it was an enabling condition, paving the road for phagocytosis. While no comparable endomembrane system ever evolved in prokaryotes [71], the facts that 1) endomembranes have evolved multiple times independently [72] and 2) the ones evolved mostly have an energetic purpose (e.g. cyanobacterial thylakoids) suggest that their forming is not too complicated. An experimentally testable question is, whether bioenergetically charged membranes can be used directly for phagocytosis. If phagocytosis can only work when all ETC and related proteins are removed from the membrane (as otherwise functionalities would interfere), it is unlikely that budding phagocytotic capabilities drove the membrane functionality change. More likely other compartments, endo- or exogenous, have already taken over bioenergetics (being more efficient) and the denuded plasma membrane later became the instrument of phagocytosis.

#### Non-photosynthetic mitochondria

Both Alphaproteobacteria and Cyanobacteria are able to both photosynthesize and generate ATP through an ETC. Apart from the photosynthetic symbiont theory (by Cavalier-Smith [36, 73], ideaoriginating from Woese [74]) no theory discussed here relies on the potential photosynthetic capabilities of the symbiont. While a photosynthetic symbiont could provide the initial benefit in the form of leaked photosynthates, most recent analyses exclude certain photosynthetic groups (Rhodobacterales [75, 76] and Rhodospirillales [76–78]) and the obligate endosymbiotic parasites of the Rickettsiales from the direct ancestry of mitochondria [76]. This does not ultimately exclude a photosynthetic ancestor, as photosynthesis can be easily lost (see plastids). Furthermore, a recent metagenomic analysis found support for an ancestor closely related to Rhodispirillales [79]. And there is one trace of possible ancient photosynthesis in mitochondria: cristae (see under Host metabolism).

#### Origin of anaerobic MROs

There is a diverse range of extant MROs varying in working conditions, metabolic functions and structural forms: there are aerobic and anaerobic mitochondria, hydrogenosomes and mitosomes [68, 80]. These types do not correspond to monophyletic groups but to ecological niches and they accordingly appear interspersed across the eukaryotic tree [32, 80, 81]. This fact might indicate that the proto-mitochondrion was a fit-for-all facultatively aerobic energy producer that selectively retained specific functions in various lineages or that there was a single function for which the ancestral mitochondria evolved and later on the various clades evolved different metabolisms; the latter meaning that anaerobic mitochondria are secondary, independent adaptations to various environments, the ancestor being strictly aerobic. Studies converge on the independent, secondary origin of anaerobic MROs [69, 81–83], though an ancestral (facultatively) anaerobic metabolism is also hypothesized (e.g. [12]). Comparative evolutionary studies investigating how conservative one method of respiration is and how fast prokaryotes can adapt from anaerobic to aerobic environments (or back), acquiring (or relinquishing) appropriate metabolisms, could lend support to either scenario. Data gathered on diverse prokaryotic groups that has a better documented correlation between function and phylogeny (better than mitochondria) could provide insight on whether the present diversity of MROs can be expected from a strictly aerobic ancestor.

### Historicals

#### Host metabolism

The ancestral metabolism of the host is unknown. Methanogenic metabolism [45, 48] is unlikely [57, 84, 85] but not impossible (see SI). Before Asgard archaea [61], the previously proposed closest living relative of the host, Lokiarchaea, were claimed to be hydrogen dependent based on genome reconstruction [86], that lends some support to the hydrogen and syntrophy hypotheses, with the caveat that no living lokiarchaeon has yet been observed. Furthermore, as mitochondria group robustly within (or close to) Rickettsiales [78, 87] (not necessarily indicating parasitic origin), but certainly not with H_2_ producing Rhodospirillales [78], thus hydrogen dependency [12, 45] might not be relevant. One can assume that the host had a mixed metabolism, autotrophic in light, heterotrophic in dark, thus both metabolic pathways were present. There is no direct evidence supporting an alphaproteobacterial (only mixed proteobacterial) origin for enzymes responsible for anaerobic energy metabolism in eukaryotes [81], as was suggested by [12, 45]. Even so, it is still possible that proteobacterial enzymes simply replaced a previously existing inferior archaeal set. On the other hand, it is still unknown if the host already had oxidative phosphorylation, as assumed by Cavalier-Smith [36]. As there is a growing amount of evidence that the ancestral symbiont was at least facultatively aerobic [9, 88, 89], it only seems reasonable to assume the host to be aerobic as well, or at least tolerating mild oxygen exposure, giving support to heterotrophic (and possibly phagotrophic) host theories. Recent phylogenomics support that LECA was most likely a heterotroph capable at least of a primitive process of particle engulfment [4, 90].

#### Symbiont metabolism

It now seems adequately supported that the ancestral symbiont was at least facultatively aerobic [9, 91, 92], capable of oxidative phosphorylation under low oxygen condition [89]. If it was strictly aerobic, then anaerobic mitochondria are secondarily descended, either by acquiring anaerobic genes via HGT from prokaryotes or other eukaryotes (both of which were rejected [6, 93]) or inheriting anaerobic genes from the anaerobic host. On the other hand, the ancestral metabolism is similarly debated as the host’s (cf. [36, 76, 78]). While there is a wide range of metabolic processes performed by extant MROs, neither ATP synthesis nor respiration is common to all [31]. Furthermore, it is unknown if the ancestor was photosynthetic or respiring (or both [36]), free-living prey or ectosymbiotic partner or was already exploiting the host. It’s true that there is no trace of ancient photosynthetic capability in mitochondria, but perhaps we didn’t look hard enough. Mitochondrial cristae might evolved prior to the symbiosis to increase metabolic surfaces for respiration or for photosynthesis, as supported by the recent finding of a homolog of cristae morphogenesis complex in alphaproteobacteria [94]. Photosynthesis is lost in the recently acquired cyanobacterial endosymbiont of the diatom Epithium [95]; this case might provide insight on how a primarily photosynthetic endosymbiont changes after metabolic integration. Recent metagenomic findings support an ancestral mitochondrion with capabilities for both anaerobic and aerobic metabolisms [79].

#### Initial relationship

The ecological setup of host and symbiont must have been different to what they are now as the ANT proteins were integrated at a later phase. Syntrophic theories [12, 45, 48, 57, 69] assume an initially mutually beneficial setup. Most of them take it granted that if there is metabolic compatibility, there will be syntrophy. However, in many cases, compatibility is not enough, and even if there is real demand at the sink and excess at the source side, there could be ecological or topological factors preventing the integration. To this day, no case is known where a syntrophic relationship among prokaryotes was turned to obligate endosymbiosis (though there are some known consortia where dependence already caused genome reduction [96]). Phagocytotic scenarios usually assume that the host preyed on the mitochondrial ancestor, but since they cannot answer how unilateral predation turned to mutual cooperation (why the prey was not digested), they also postulate a pre-existing metabolic symbiosis [9, 16, 36, 41]. To find out what the first exchanged substrate was between host and symbiont, the first carrier protein inserted into the mitochondrial outer/inner membrane must be found. Perhaps the most common transport protein found in any other organelle in the eukaryotic cell could shed some light on the original carrier. On the other hand, the relationship might not have been mutually beneficial at all, but parasitic [56, 57], where the host didn’t receive anything from the symbiont. This poses an ecological problem: there had to have an advantage for the pair as a unit, even before ANT integration, otherwise the relationship was evolutionarily unstable.

#### Early selective advantage

Metabolic compartmentation and ATP exporting are the end results and were not in effect when the relationship started. Since it was not necessarily mutual, any theory should account for how a possibly costly early relationship become advantageous and stable in evolutionary terms. Phagocytosis only allows endosymbiosis, if the host does not eat all its engulfed prey. This was addressed by Szathmáry [3], suggesting either a metabolic “bribe” [36] or internal farming [1]. The prey, providing photosynthetic metabolites for the host, could provide the selective edge daytime, especially when no heterotrophic resources are available, while respiration goes on in the dark [36]. Blackstone proposed that selective digestion (necessary for farming) could happen under stressful conditions, if early endosymbiont was capable of emitting ATP to stabilize a hungry host and avoid its own (and possibly its copies’) digestion [2]. According to Gray, the metabolically active endogenous pre-endosymbiont [41] was replaced by mitochondria as they had their own energy source and didn’t rely on the host’s ATP [41]. The problem is that the ANTs (if used before to pump ATP into the organelle) had to be immediately reversed in function (or directionally correct ones had to be recruited from other sources, e.g. the peroxisome according to [36]) otherwise the symbiont would have consumed all the host’s cytosolic ATP. This again points to parasitic scenarios [56, 57]. Similarly to farming, parasitism couples with reproductive costs. However, initially defective parasites could induce resistance and ultimately dependence in the host [97]. The question is, how to survive the infection for a period long enough for evolution to tame the parasite.

#### Mechanism of inclusion

The symbiont either was engulfed via phagocytosis or via slowly increasing surface contact during ectosymbiotic syntrophy, or was a bacterial invader (see Table 1 and Additional file 1: Table S1). Since there is no third host-derived membrane wrapping the symbiont, it either was degraded, or never existed (e.g. [90]), suggesting a non-phagocytotic host or early escape from a phagosome. Phylogenomics support that a dynamic filament system is far from impossible in prokaryotes [43, 59, 98–100] and primitive phagocytosis could have existed even without a dynamic actin cytoskeleton [101]. If the host had a cell wall (most archaea do) it was lost eventually. Insertion of transporters by the host was probably easier when there was one less membrane, however, degrading the phagosomal membrane must have taken time, which further prolonged a possibly unstable relationship lacking proper exchange mechanisms. Parasitic origins [23, 56, 57] however do not require expensive machinery from the host side and there is no extra membrane to get rid of. As the parasite was probably already equipped with a nucleotide transporter that could steal ATP from the host, exploitation could have been easy [89, 102, 103]. Problem is then in evolutionary stability.

#### Vertical transmission

At any point during eukaryogenesis the newly forming chimaeric cell had to divide so that its selective advantage was heritable and cooperation could fixate. How did the initial partnership (mutual or unilateral) remain stable without means to pass on adaptations and without the control of possible selfish mutations? It is extremely hard to explain conservative vertical transmission of the proto-symbiont in syntrophic scenarios [45, 48, 84]. Again, parasitic theories have the better hand here as parasites naturally replicate inside the host and are distributed when the host divides.

## Conclusions

The integration of mitochondria was a major transition, and a hard one [3]. It poses a puzzle so complicated that new theories are still generated 100 years since endosymbiosis was first proposed by Konstantin Mereschkowsky [104] and 50 years since Lynn Margulis cemented the endosymbiotic origin of mitochondria into evolutionary biology [39]. The challenge and singularity of eukaryotic origins lie in the fact that the resulting new unit is not just an amalgamation of organisms of different ancestry but also because lower-level units overtook energy metabolism [2]. New phylogenetic data are trickling in each year, shining light to new pieces of the puzzle, and old and buried theories are dusted again (e.g. [56]).

One would expect that by this time, there is a consensus about the transition, but far from that, even the most fundamental points are still debated. Major discrepancies are in the nature of the host and inclusion mechanism, but of course these aspects have far reaching dependencies. While there are strong arguments on all sides, the debate about engulfment or infection, and early or late phagocytosis is still ongoing (see [23] and comment [105]; [7] and responses [20, 106], respectively).

In the last few decades, some have realized that the real question lies in the initial relationship that predated the nucleotide translocase insertion. Blackstone’s scenario points out the fact that even if the metabolic coupling is feasible, one has to comply with ecological considerations and – to run a selectively superior joint – one has to deal with occasional subordinate partners (mutants).

Asking the right questions helps evaluating contending hypotheses. Each question examined in this paper refers to processes that once surely happened thus have to have a purely mechanistic explanation, that complies with bioenergetics but also with ecology. The number of unanswered questions piling up clearly indicates the need for better mechanistic models with testable predictions, focusing more on ecology than on metabolism. We have objectively investigated eight theories of mitochondrial origins, some mainstream some less famous, through the same twelve questions. These questions stem from the evolutionary drive behind endosymbiosis, accordingly all of them had to be accounted for by a plausible mechanistic model (existing or future) of mitochondrial origin.

Interestingly, not all host-symbiont combinations were proposed (see Table 1 and Additional file 1: Table S1). For example syntrophy where both partners are aerobic bacteria and archaeal genes are acquired via HGT or by a late endosymbiotic archaeon; or an aerobic, phagocytotic archaeal host (early theories still hold strong on the widespread anoxic nature of hosts); or a bacterial host that was invaded by parasitic archaea and alphaproteobacteria (*Nanoarchaeum equitans* is the only known archaeal parasite).

There is no single theory that can adequately answer all questions (see Table 2). Furthermore, and more importantly, some answers have turned out to be untenable in light of new results. Of course, hypotheses are not mutually exclusive and there are enough theories to have at least one acceptable answer to all critical points. One might be able to piece together the compatible parts to craft a scenario that maintain a causal and cohesive course of events. We omitted this reconstruction as that would itself constitute a new hypothesis. It wouldn’t necessarily be superfluous but would certainly increase the size of the paper. It is left for future work.

**Table 2:** Summary of hypotheses and how they account for the unavoidable questions of mitochondrial origins

	Hydrogen hypothesis [12, 45]	Photosynthetic symbiont theory [36, 108]	Syntrophy hypothesis [48]	Phagocytosing archaeon theory [16]	Pre-endosymbiont hypothesis [9, 41]	Sulfur-cycling hypothesis [57]	Origin-by-infection hypothesis [57]	Oxygen detoxification hypothesis [68, 69, 103]
Eukaryotic singularity	✓			✓				✓
Lack of intermediates	✓						✓	
Chimaeric nature (membrane conversion)	✓ (✓)	✓ (✓)	✓ (✓)	✓ (?)	✓ (?)	✓ (?)	✓ (?)	✓ (?)
Lack of membrane bioenergetics in host	✓	✓	✓		✓			
Non-photosynthetic mitochondria	✓	✓	✓					
Variety of mitochondria	✓	✓	✓					✓
Metabolism of host	✓	✓	✓	✓	✓	✓	✓	✓ (?)
Metabolism of symbiont	✓	✓	✓	?	✓	✓	✓	✓
Initial relationship	✓	✓	✓	✓ (?)	?	✓	✓	✓
Early selective advantage	✓	✓	✓		✓			✓ (untenable)
Mechanism of inclusion	✓	✓	✓	✓	✓	✓		
Vertical transmission							✓	

A checkmark indicates that the hypothesis reasonably accounts for the observed facts and complies with empirical data (even if debatable). A blank cell indicates that it is unclear how the theory deals (if at all) with the given question

The theory that provides the most answers is the hydrogen hypothesis, but it does not mean that it is the single valid hypothesis. It still has some holes and debatable claims (lack of a host-derived membrane wrapping mitochondria; vertical transmission of intermediate syntrophic stages; membrane replacement; primarily derived MROs). No syntrophic case is known where the strong metabolic coupling actually lead to obligate endosymbiosis among prokaryotes. From a mechanistic point of view, phagocytosis is more likely than syntrophic inclusion [71].

The fact that Archaezoa were not found does not mean they never existed, as Martin often claims [44]. Similarly, there is no phylogenetic or modern evidence for prokaryote-prokaryote (especially bacterium-in-archaeon) syntrophy that lead to obligate symbiosis, but this does not seem to hinder Martin’s argument that such a hypothetic interaction lead to the first mitochondriated eukaryote. While the two cases are analogous in that they both lack the crucial evidence for now, the fact that new archaeal groups are recognized due to rapidly expanding metagenomics and these groups constantly provide new evidence to the field indicates that it is only expectable to find clues in new groups that turn out to be relevant to the archaeal host debate. Most recently it was demonstrated that “several fundamental building blocks for the evolution of a primordial vesicular machinery derive from the archaeal ancestor of eukaryotes rather than from the mitochondrial endosymbiont […] origin of the eukaryotic trafficking machinery predates the mitochondrial origin” [61].

Furthermore, the bioenergetic argument put forward by Nick Lane, supportive of early mitochondria, is debated [3, 107]. The important point is that gaining energy cannot be explained with the mitochondrion, as initially it did not provide much. Consequently, any reasoning about the energy requirements of early eukaryogenesis must rely on a *gradual increase of energy*. In this light, the source of extra energy might as well have come from the simplest possible source: increasing energetic membrane surfaces by internalizing respiration. Endomembranes evolved for example by photosynthetic cyanobacteria are able to power multicellularity (though still far from eukaryotic levels). There exist phagocytotic eukaryotes lacking active mitochondria proving that phagocytosis can be sufficiently powered without the powerhouse.

Finding clues of ancient bioenergetics in eukaryotic membranes is extremely important to find out if endomembranes have ever evolved for energetic reasons. The endoplasmic reticulum does require oxygen. If it evolved to increase the bioenergetic surface, it could explain the missing energy required for an active cytoskeleton. The respiratory chain in the endoplasmic reticulum can be a relic of the protoeukaryote’s plasma membrane ETC [108], with the ancestral V-ATPase playing a part in early oxidative phosphorylation. The fact that mitochondria implement metabolic compartmentation, while the early endomembrane system simply increases surface provides the advantage of mitochondria over endomembranes. This can be further pursued by analyzing the ETC proteins in eukaryotic endomembranes. To our knowledge, there is no explicit theory that ever explored the possibility that invaginating bioenergetic membranes powered phagocytosis and early eukaryotic evolution before mitochondria. It certainly worth an investigation.

The only theory that actually speculated about an initially disadvantageous role of the host was the infection hypothesis by Searcy [57] (taken up recently by [56]). This is understandable as many build on the implicit assumption that if something is working now, it also had to start from cooperative benefit, otherwise it wouldn’t have started at all. However, an evolutionary relationship doesn’t need mutually beneficial starting conditions: it only requires that the partnership is ecologically stable, so that evolutionary adaptations have enough time to accumulate. Ecological stability can be achieved in many ways, and mutual cooperation is only one. The cooperative end stage should not blur the focus in our assumptions about initial factors.

Postulating early parasitism has many positive corollaries apart from the straightforward mechanism of inclusion. It also accounts for the extreme streamlining and seemingly rapid evolution of the symbiont, as the parasite can quickly get rid of unwanted genes relying on the host. As plastid ancestors were never parasitic, and as such, were merely food for the phagocytotic host, parasitism naturally provides an explanation why mitochondria arose only once (compared to plastids). It was the entirely different initial relationship responsible for the different evolutionary outcomes. There are results suggesting an early parasitic relationship – which is entirely not surprising, considering that parasitism is initially more common than mutualism between two species. However, phylogenetic association with Rickettsiales could simply mean an ancestrally free-living protomitochondrion or could entirely be an artefact of long branch attraction, considering the fast evolution of modern parasites.

For an evolutionary adaptation to go to fixation it must be preceded by an ecological equilibrium where partners coexist for a prolonged time. The solution to mitochondrial origins and eukaryogenesis lies in this early relationship and, in turn, due to a probably unstable proto-nuclear host lineage, it is a question of ecology rather than evolution. If, however, early ecology was costly for the host, as the symbiont was rather a parasite at the time, the host had to receive some indirect benefit from the relationship to achieve the unprecedented success eukaryotes exhibit today.

## Reviewer comments and author responses

### Reviewer #1: Michael gray

#### Reviewer summary

In this review, Zachar and Szathmáry provide a refreshing assessment of various hypotheses for the origin of mitochondria within the broader issue of the origin of the eukaryotic cell (eukaryogenesis). To establish a framework for comparing and contrasting the various hypotheses they discuss, the authors set out 12 questions that they assert a robust hypothesis should be able to answer. In the end, they conclude that none of the current hypotheses is able to do so, differing in the extent and degree to which they individually address each of the questions. This review constitutes a most interesting and very valuable contribution to the on-going debate about the origin of mitochondria. I thoroughly enjoyed reading it (several times!). Both the main text and the supplemental information are comprehensive (indeed exhaustive) in their coverage of the literature. The framework set out by the authors will challenge proponents of the various hypotheses to revise/reformulate them in order to try to take account of the points raised. The overview and the extensive reference list will prove particularly useful for researchers in this specific area as well as for less well informed readers interested in an in-depth look at the current state of the field. A hallmark of this submission is its thoughtful and balanced critique of both the strengths and weaknesses of the various proposals the authors evaluate.

***Author’s answer:*** *We are extremely grateful for Michael Gray for the enthusiastic review and especially for the thorough reading and meticulous checking of sources and citations! It has provided an enormous help for us to improve the manuscript. We indeed hope that our framework would challenge proponents of all hypotheses, thanks! However, it is more likely that some of them will close their eyes and ears.*

#### Reviewer recommendations

The authors raise innumerable points that could be discussed/debated at length, and I’m sure they will be, among aficionados in the field. Here, I will restrict my comments to points that I feel the authors need to correct or consider further.

### Main text (PDF file)

1. Two very recent papers that undoubtedly appeared after the authors submitted their paper are highly relevant, and should be included in the reference list, and discussed. (1) Niedzwiedzka K et al. (2017) Asgard archaea illuminate the origin of eukaryotic cellular complexity. Nature) 541,359–364. This paper adds to the discussion of the nature of the eukaryotic ancestor, in particular the finding of “eukaryote signature proteins” in an archaeal lineage that appears to be the sister group to eukaryotes. (2) lynch M, Marinov GK (2017) membranes, energetics, and evolution across the prokaryote-eukaryote divide. eLife 6:e20437. This paper addresses the controversy about whether the advent of mitochondria provided a huge increase in cellullar energy that was responsible for the diversification, increase in complexity and appearance of multicellularity in eukaryotes.

*We now refer to these publications throughout the text.*

2. **pg. 6, lines 17–18**: The reference to “the respiring pre-endosymbiont [39]” is incorrect. In that particular hypothesis, the pre-endosymbiont is an endogenously evolved, membrane-bound compartment that has a protein import system, a variety of ion/ metabolite transporters, and various metabolic pathways, but does NOT have an ETC or respire. In the Supplemental Information (pg. 4–5), the pre-endosymbiont hypothesis is accurately summarized, but elsewhere it is referred to incorrectly as being an energy-generating/respiring entity.

*Indeed, that reference, and others, were wrong, we have now corrected them.*

3. **pg. 6, line 28**: Regarding the question of “whether bioenergetically charged membranes can be used directly for phagocytosis”, one possibility (if one envisages a heterotrophic phagocytic host having a bioenergetic cell membrane) is that ETC complexes might be non-randomly distributed in such a membrane, such that phagocytosis is limited to a specialized region that lacks such complexes.

*We are thinking exactly along these lines. However, since this is purely speculative for the moment, we did not want to include this in the paper yet.*

4. **pg. 6, line 40–41**: The authors cite ref. [54] in support of the statement that “recent analyses exclude ... the obligate endosymbiotic parasites of the Rickettsiales from the direct ancestry of mitochondria.” What these authors actually said was, “Our results suggest that mitochondria most likely originated from a Rickettsiales endosymbiont already residing in the host, but not from the distantly related free-living Pelagibacter and Rhodospirillales.”

*Our intended meaning is in the ellipsis: “most recent analyses exclude* ***photosynthetic species (Rhodobacter and Rhodospirillum)*** *and the obligate endosymbiotic parasites of the Rickettsiales from the direct ancestry of mitochondria* [54, 70–72]*”. It was an unfortunate compression of citations, where only Degli-Esposti et al. excluded obligate parasites, while others (and Degli-Esposti et al. also) excluded photosynthetic species (Rhodobacterales and Rhodospirillales). We have corrected it.*

5. **pg. 8, lines 16–21**: The issue of the acquisition (when and from where) of the ANT ultimately used to pump ATP out of the evolving mitochondrion is an important one. In the pre-endosymbiont hypothesis, the initial state is one in which host ATP is transferred from the cytosol into the pre-endosymbiont to support metabolic functions. The endosymbiont, unless it is an energy parasite, would initially generate its own ATP and not use that of the host, having no ANT for transporting ATP into the cytosol. Only after the endosymbiont acquires the directionally correct ANT to allow it to supply ATP to the host (this change need not have been a rapid one) will it have a selective advantage over both the pre-endosymbiont and whatever energy-generating system the host is using. Although the hypothesis suggested a functional reversal of the original pre-endosymbiont ANT (from ATP in, ADP out, to ADP in, ATP out), it is possible that it was acquired from elsewhere, perhaps from the peroxisome, as suggested by Cavalier-Smith, if in fact the peroxisome predated the mitochondrial endosymbiosis and is not, as I had suggested, a possible remnant of the pre-endosymbiont.

*These are indeed both possible, and would reduce the requirement for immediate change; we’ve extended the sentence to accommodate both alternatives.*

6. **pg. 8, lines 28–29:** The fact that there is no third membrane (representing the host plasma membrane) surrounding the mitochondrion really doesn’t discriminate, in my opinion, between phagocytotic and non-phagocytotic inclusion mechanisms. The endosymbiont could readily have ‘escaped’ from an engulfing phagosome, especially if the mitochondrial endosymbiosis occurred at a relatively early stage when the phagocytotic machinery was still primitive and inefficient. I personally find the alternative ‘slow engulfment’ scenario (archaeal host and bacterial symbiont) difficult to envisage, given that bacterial symbionts in an archaeal host have never been reported, particularly if the host had a cell wall (as is likely).

*It is true, that the lack of the phagosomal membrane doesn’t prove anything when uncovering this ancient crime. But only one aspect is the mechanism and the other is which was more ecologically stable. In our opinion, the systematic escape from phagosomes renders the prey a parasite, which poses different short-term coexistential (ecological) problems than the matter of a slowly (i.e. in evolutionary timescale) degraded phagosomal sheath. We have indicated though the options in the text. The cell wall (if existed) had to be lost either way, regardless whether the inclusion was parasitic, syntrophic or phagocytotic.*

#### Supplemental information

1. **pg. 8, “3. The chimaeric nature ...”, 2nd para.**: The authors state that “the hydrogen hypothesis assumes that it was the endosymbiont that provided the bacterial genes for glycolysis ...”, and later in the same paragraph, “Even if modern glycolysis is of alphaproteobacterial origin ...”. Also, pg. 12, “7. Metabolism of the host”, para. 3: “The fact that most of the eukaryotic energy metabolism is of proteobacterial origin (glycolysis, TCA cycle, etc. …” However, in one of the cited refs. [169], Canback et al. concluded: “In fact, we cannot identify a single eukaryotic glycolytic enzyme family within the present cohort that clusters in a single node with α-proteobacteria or with any other modern bacterial phylum.”

*That was our mistake. Proteobacterial origin of these enzymes is not proven thus we shouldn’t have considered it as a fact. Corrected. Canbäck et al.’s (and others’) result is also a strong evidence against those who claim that the host inherited its glycolytic enzymes (and heterotrophic lifestyle) from the alphaproteobacterial symbiont.*

**2. pg. 11, “5. Non-photosynthetic mitochondria”, 1st para**. The authors cite Cavalier-Smith with regard to the proposal that “the ancestral mitochondrion was a photosynthetic purple non-sulphur bacterium.” Carl Woese made the suggestion early on [Woese, Carl R. Endosymbionts and mitochondrial origins. Journal of molecular evolution 10.2 (1977): 93–96] that the mitochondrion “was initially a photosynthetic organelle, analogous to the modern chloroplast.”

*Thank you for the original source, we’ve included a reference there.*

**3. pg. 17:** “Bacterial intracellular predators are known, e.g. Bdellovibrio …” Margulis, in [186], termed this bacterium an amazing example of prokaryote-prokaryote “emboîtement” “without phagocytosis”, suggesting it as a possible protomitochondrial candidate (“it is likely that protomitochondria invaded their hosts just as modern predatory bacteria Bdellovibrio invade prey bacteria”). The problem is that Bdellovibrio very effectively destroys its ‘host’ bacterium in the process of invading it, which does not exactly predispose it to entering into a stable relationship with the invaded organism.

*Yes, we are aware of it, though we failed to make this explicit. Added a sentence about host death.*

**4.** [74] is missing from the list of supplemental references.

*Corrected.*

#### Minor issues

pg. 1, line 25: “...a valid hypothesis ...”

pg. 2, line 44: “... integration started, though there is some ...”

pg. 3, line 4: “... the theory has to fit ...”

pg. 4, line 2: “... We restrict questions ...”

pg. 5, line 23: I would say “Absent” rather than “Lacking”.

pg. 5, line 37: “...disappeared or they have not been found yet.”

pg. 5, lines 37–38: delete sentence fragment beginning “Considering that more ...”

pg. 5, line 39: “... that have been found in prokaryotes ...”

pg. 5, lines 57–58: “... while inheriting the heterotrophic lifestyle ...”

pg. 6, line 55: “... metabolisms, the latter meaning ...”

pg. 6, line 56: “... various environments, the ancestor being ...”

pg. 7, line 12: “... no living lokiarchaeon has yet been observed ...”

pg. 7, line 36: “... or was already exploiting ...”

pg. 8, line 3: “... evolutionarily unstable ...”

pg. 8, line 43: “... remain stable ...”

pg. 9, line 13: “The number of unanswered questions ...”

pg. 9, line 17: “These questions stem from ...”

pg. 9, lines 45–46: “... might as well have come ...”

pg. 9, line 52: “... endomembranes have ever evolved...”

*All minor issues were corrected.*

### Reviewer #2: William Martin

#### Reviewer summary

If the authors are upset at what I have to say, recall that they asked me to publically review their paper in this journal. This paper reviews a few theories about mitochondrial origin and passes judgement upon them, which has become a very popular undertaking of late, especially among authors who have no theory of their own. I like the result (the hydrogen hypothesis fares well under heir test), but…. The paper evokes the impression of being more complete and scholarly than it is, as I will point out in these comments. In their highlights, the authors conclude that no present hypothesis deals with their twelve criteria, but they cover a very small segment of existing hypothesis and their twelve criteria miss the most important phylogenetic observations (a property shared by population genetic based approaches to eukaryote origin that avoid endosymbiosis as an evolutionary mechanism). Their statments that phagotrophy is more likely than syntrophy are a restatment of a 50 year old idea and have no support in any kind of evidence, a view that furthermore supposes that evolution has no energetic price, one can just print as much ATP as one needs to surmount any evolutionary hurdle. Were it that easy, many different groups of prokaryotes would be phagocytotic, hence one gets the impression that the authors are not really paying attention to the observations (in addition to the theories).

***Author’s answer:*** *We are not upset at all about Bill Martin’s review, instead we are grateful for his honest critique, corrections, and the important points he has raised. There might not have been enough room to include all his suggestions, but we are certainly open to improvement. On the other hand, we don’t agree with Martin on a few points. Firstly, passing judgement on hypotheses would be perfectly legitimate when the setup is declared to be a review based on objective evolutionary criteria, however, we didn’t really judge any of the theories too harshly (the hydrogen hypothesis is indeed the one that answers the most questions). We tried to be objective, and we let the Reader decide about whether we succeeded or not. As Bernard Show famously said after having been attacked to act as a musical critic while not being able to produce music: „I cannot lay eggs but I can tell whether an egg is bad or not. Furthermore, Martin is mistaken that we don’t have our own theory: we do (recall the farming hypothesis by Maynard Smith and Szathmáry from 1995 [1]), but since this was intended to be a review only, one does not discuss his own theory in it, because 1) of size reasons and 2) it might bias the outcome of the analysis – researchers usually being overly fond of their own ideas. Be assured, that when our own hypothesis is published, it will be judged along the very same criteria. As a matter of fact, this was our aim: any new idea should adhere to these (and possibly more) criteria.*

*Concerning the energetic price of evolution: as one of us notes [2], there is no theoretical, comparative, or experimental evidence yet, beyond Haldane’s cost of selection, that such a price exists. Extraordinary claims require extraordinary evidence.*

*Including more hypotheses (more or less mainstream) would also cause serious size-problems as the manuscript is already barely fits the limits of publishability. As a matter of fact, in an earlier version of this manuscript there were many more theories discussed, though they were sacrificed to achieve a reasonable page number.*

#### Reviewer recommendations

**Page 2 line 40.** they say that the eukaryote common ancestor was most likely a phagocytosing heterotroph. On the basis of what evidence (not from what opinion), do they infer the presence of phagocytosis in Leca? What comparative studies indicate that phagocytotic processes in the eukaryote supergroups that possess phagocytosis are homologous?

*The possible archaeal origin of phagocytosis and a possibly (primitively) phagocytotic LECA were discussed by many [3–6]s. While it is true that there is no comparative phylogenomic analysis favoring a single, archaeal origin of phagocytosis, there are at least two strong arguments for a phagocytotic LECA. First, phagotrophy is so widespread in eukaryotes, that it is only parsimonious to assume its ancestry, possibly only of a core set of essential proteins. Interestingly, Yutin et al. question whether the cyanobacterial plastid is engulfed via bona fide phagocytosis, it is quite clear from all subsequent plastid inclusions that they were acquired via phagocytosis, sometimes they even retain the phagosomal membrane [7], so it is only reasonable to assume that the cyanobacterium was also captured this way. Certainly no parasitic cyanobacteria are known, neither prokaryote-prokaryote syntrophic inclusion. While the phagocytosis in wall-less prasinophytes (see [8, 9]) is different than in other eukaryotic groups, the basic mechanism and molecular requirements are the same. Also, the claim that Fungi seems to be ancestrally phagocytotic (Rozella wall-less basal fungus in Cryptomycota, possibly capable of phagocytosis and closely related to obligately phagotrophic aphelids [10–13]) strongly suggests ancestral phagocytosis in LECA, as all eukaryotic supergroups are then primarily phagotrophic. Hence we maintain that there are no primarily non-phagocytotic eukaryotes, even if phagocytosis was primitive – which of course is not a well-defined term.*

*Second, many of the necessary eukaryotic proteins for the actin-based cytoskeleton, membrane remodelling, vesicle formation, etc. have homologues in archaea (l small GTPases) [5, 14]. Koonin [15] states based on comparative genomic analyses: “Taken together, these results clearly indicate that LECA was a typical, fully developed eukaryotic cell” while also inferring an archaeal origin for at least some key phagocytosis-related-proteins. Independent of these, we note that for mitochondria to be engulfed by primitive phagocytosis it is not required that all eukaryotic phagocytoses are of a single origin.*

*We also seize the opportunity, that as Cymbomonas, Rozella, the Loki and Asgard archaea, Paulinella and others were discovered and provided evidence against theories excluding their possibilities, so can we find Archezoa once. We don’t claim that it existed, but the argument of lack of evidence in microbiology seems to be sufficient only temporarily. Finding archezoa (or phagocytotic archaea) in the microbiota (and failing) is definitely not like trying to find DNA (and failing) in the undulipodium.*

**p.2 l. 54.** Boldface type. This is obvious, who argues the contrary?

**p. 2 l. 57.** "We address mitochondrial origin and only tangentially discuss eukaryogenesis"... That conveniently biasses the whole paper in favour of the view that symbiosis had nothing to do with eukaryogenesis, which is exactly what population geneticists have always thought, becuase symbiosis does not fit in the gradualist point mutation (drifty) popgen view. If symbiosis was essential to eukaryogenesis, as symbiogenic models posit, then the tangent they disregard is actually the main road.

*We never stated, that symbiosis was not essential for eukaryogenesis – on the contrary, we strongly believe (and claim many times) that the singularity of both eukaryotes and mitochondria are not coincidences but are causally coupled. We certainly didn’t want to imply the bias Martin attributes to us. Accordingly we corrected the sentence to say: “only tangentially discuss other aspects of eukaryogenesis, e.g. the origin of the nucleus” as that is what better describes our original intent. However, let us remind our worthy Referee that symbiosis is regarded as one of the main ways to increase in complexity in The Major Transitions (1). We have nothing against popgen (it is part of our trade), but we have always gone significantly beyond it, whenever necessary.*

**p. 3 line 4.** fit the phylogenetic data. Which phylogenetic data do they mean. The phylogenetic data are generally conflicting on everything more ancient than human and chimpanzee. Phylogenetic data that show that the vast majority of eukaryotic genes are bacterial in origin rather than archaeal in origin would bneed to be taken into account (ignored in the paper): Esser et al. 2004; Pisani et al. 2007 supertrees MBE; Cotton and McInerney 2010 PNAS; Thiergart et al. 2012; Ku et al., 2015). As I pointed out with Dagan (The tree of 1 %) the bacterial majority of genes in eukaryotic genomes is biology’s best kept secret, it stays secret here as well, even though the authors (roghtly) demand that theories account for the phylogenetic data. I say that the thrioes need to account for all the data and some level of resolution. I have been doing genome wide phylogenies of all genes for a long time, so has McInerney. Folks who do not like the results (for example Lopez Garcia and Moriera this year in JTB) just ignore it and go on reciting their favorite eukatryote origin stories, the stories that are not supported by the genome wide data. I recently sent Szathmary my unpublished manuscript on phagocytosis, which makes exactly the distinction we see here in this paper.

*We agree with Martin on that a valid theory should agree with all the phylogenetic data. The problem is usually that scientists do not agree on how to interpret the data, and – a more objective matter – the phylogenetic data we evidence is a moving target that could change our perspective significantly as new data comes in.*

**p. 3 line 36 and l. 45.** I am not going to go into the treatment of the literature in these two sections, but I would ask them to go back and read my 2001 review of endosymbiotic theories for eukaryote origin in Biol. Chem.

**p. 3, Line 46:** symbiogenesis named by Koonin in 2015?! Oh come on, read the literature. Only wrong by 105 years, try Merechkowsky 1910 (the word Symbiogenesis is in his title, you even cite it) or Cavalier Smith or Margulis or my papers, come on, read the literature before you start to claim that xyz named this or that.

*Indeed this (and the next point) was an incorrect reference, we’ve removed it.*

**p. 3, Line 50:** fusion named by Forterre?! Ich muss lachen. In 2001 I reviewed several models that used the word fusion to describe aspects of their theories for eukaryote origin: Zillig, Lake, Gupta, Margulis all talked about fusions. Forterre just ridiculed symbiogenesis, in fact he has gone completely silent on eukaryotes now that most folks seem to agree that eukaryotes arose from prokaryotes, not vice versa.

*As with the above point, the reference was removed.*

**p. 3, l. 57**. Investigate the case in detail? What did that paper inverstigate in detail. Ref [14] is a brilliant example of people passing judgement on a literature that they do not know well and evaluating theories where they have none of their own. Ref [14] is a kind of theory parasitic literature, a new kind of paper nowadays. By about p 4 I am convinced that the authors have not read many of the papers that they cite and I loose interest in the text because it recites dogmatic liturgies that we know very well. For example...

*Poole and Gribaldo did raise some important criteria and they have discussed intermediate cases (other than mitochondria-first and -last scenarios), and our reference citing them only reflects these facts, nothing else. We didn’t want to imply that they did an in-depth analysis, thus we have modified our sentence.*

**On page 5, line 4**, there is a quote from which the mindset of these authors (and many others) very clearly comes into focus: "Firstly, it goes against the concept of gradual evolution of complex traits...denying the possibility of stepwse accumulation of adaptations." As if that were a flaw. In prokaryotes and definitely at symbiogenesis (symbiosis) it is a distinct virtue. That quote makes it clear that the authors are convinced that i) evolution is always gradual, and ii) that adaptation is the cause of all evolutionaery change. I comes from physiology, and if we look at the origin of plastids (primary or secondary) there was nothing gradual about it, and furtheromore in physiology, single genes are useless, as physiology is composed of parthways and larger units of function (photosynthesis, respiration), and as such transitions many many genes tend to change hands in the cases we know (Martin et al. 2002 “thousands of cyanobacterial genes” PNAS; Nelson Sathi et al. “acquisition of 1000 eubacterial genes” PNAS; Ku et al. 2015, “endosymbiotic origin” Nature). So, like Lynch, the authors close thier eyes to what is actually happening at symbiotic events (gene transfer) that mark major transitions in the microbial world and hold the gradualist party line. Fine, but then the rest of the argumentation becomes less and less convincing, so much so that I put the paper down, happy that they find the hysdrogen hypothesis to be the best theory out there by their criteria, though their criteria are designed along lines that have nothing to do with physiology. For example, on page 9, around line 56, they start talking about the ER as an oxygen requiring membrane (except in anaerobic eukaryotes, of course, chuckle....) and Cavalier Smith’s invagination theory again (archezoa). In summary, this is last paper I will ever review for Biol. Direct beacuse I have to write something that will go into print when I would rather be doing my own work. Physiology is apparently a lot harder than it looks.

*Concerning rapid, non-gradual appearance of plastids, one has to consider that membrane-invagination, phagocytosis, protein-import mechanisms and other eukaryotic features facilitating symbiotic integration were already in effect and need not have to be invented again. Moreover, note that there is a long way from endosymbiosis to organelle. Especially for the mitochondrion (whether early or later) literally thousands of advantageous mutations must have gone to fixation by natural selection. Exaptations are not adaptations.*

**p. 5 line 42**. "Phylogenetic analyses support a complex archaeon"? That is not true. The Loki and Asgard lineages are hope to be complex. The phylogentic analyses support an archaeon, one apparently with H_2_-dependent metabolism (Sousa et al., Nature Microbiology 2016). The phylogenetic analyses are only for the archaeal component of eukaryotes anyway, which as explained above is a very small minority of eukaryotic genes. If someone would finally publish images of the Lokiarchaea type archaea so that we could see how small and umcomplex they are the world would be a lot better off. The authors are falling into the same trap as Koonin and believing that Loki and asgard archaea are archezoa. There is no such evidence. But let them believe what they want to believe.

*We have corrected our sentence to read: “Phylogenetic analyses support the* ***theory*** *that the archaeal host already had cytoskeletal features and possibly even primitive phagocytosis […]”. We add it here, that we also eagerly await seeing the actual cellular complexity and functions of Loki and Asgard archaea.*

**p. 5, line 46.** "It is a fact that many eukaryotic features are existing in archaea". Wrong, The authors are making the same mistake as Ettema: They see a protein homologous to ESCRT and say “archaea have an endomembrane!” Wrong. The ESCRT homologues are called CDV in archaea for cell division, and that is what they do, they pinch off cells to the environment, not vesicles to the cytosol. The authors are uncritically believing everything they read that supports their hope for the Archezoon.

*We have corrected our fallacious statement according to most recent results.*

**p. 5. Chimeric host**. How do these authors come to the conclusion that the host was chimeric before the origin of mitochondria?? Maybe they believe Pitts and Gabaldon (2016), a complete artefact in every respect (Martin et al. Late mitochondrion is an artefact, GBE 2017).

*We never stated (or believed) that the host was chimaeric* ***before*** *the appearance of the symbiont. We have corrected “chimaeric host” to “chimaeric eukaryotes” for clarity. Nevertheless, if one accepts early phagocytosis, it is only reasonable to assume that the host has integrated all sorts of bacterial and archaeal genes prior to the nucleus being established.*

**p. 9, line 38.** “No syntrophic case actually leads to endosymbiosis”. Wrong. Methanogenic endosymbionts in ciliates. Fenchel, Finlay, Embley, Hackstein, dozens of papers from the 1990s. This paper is just like Poole and Penny boasting about how true and correct and invulnerable to error the three domain tree was, in the face of many papers that showed that it was wrong. Now the evidence indicates that it was really really wrong. But just ignore the evidence and make sweeping statements. I am just picking up on the most glaring problems, life is so short. “No syntrophic case actually leads to endosymbiosis”. Wrong. “What is true is that no mitochondrion lacking cell has ever demonstrably evolved phagocytosis”.

*That was our mistake, we have corrected the sentence to read: “No syntrophic case actually leads to* ***obligate*** *endosymbiosis* ***among prokaryotes*** *”. The Reviewer’s last sentence (“What is true is that no mitochondrion lacking cell has ever demonstrably evolved phagocytosis”) is only true if one accepts the hypothesis that ancestral eukaryotes evolved phagocytosis* ***after*** *mitochondria and not the other way around, which is not proven yet (the debate is exactly about this).*

Figure 1 Everybody is now drawing collections of theories for eukaryote and organelle origin like I did (starting in 2001, Biol. Chem.). Please have the courtesy to state where the practice of comparing these theories in this fashion came from. Any reader unfamiliar with the literature will think that the authors invented this kind of review and comparison themselves, which is not true, they just like siuch figures (“in many of my reviews”) and wanted to do something similar

*Actually, the idea of doing an objective critical comparison came from a very different source, from Számadó & Szathmáry: Selective scenarios for the emergence of natural language [16]. We originally omitted citing this paper as its topic is not related to the manuscript, but now it is included in the Background section. The suggestion what the Reviewer is hinting at (“someone, possibly him, „invented this kind of review and comparison”) is false, in the same way as he attributes this suggestion to us. Reviews of this kind existed well before any us. Nevertheless, we’ve included reference to his works in the figure caption, acknowledging his profound and pioneering works*.

Figure 1 **panel 2** photosynthetic symbiont. Sigh. Wrong. That was not Cavalier Smith’s idea. Go to the literature and look for “a purple protist” by Bernard and Fenchel.

*We are aware that it was Woese who first suggested the photosynthetic symbiont (it was also pointed out by another Reviewer). We have included further citations to his work now. In this paper however, we’ve focused on Cavalier-Smith’s version of the photosynthetic symbiont theory as this provides the most detail of all. The figure is the exact listing of eight specific (to certain scientists/labs) theories discussed in detail within the paper (and in the Supplementum).*

I have corrected enough in this paper. Really. The weakest and most glaring problem with the present paper is that the authors do not present their own proposal. They just pass judgement over other people’s ideas. This is how a lot of people have gotten by over the last 30 years. What the present authors are saying here is somehow very reminiscent of Gray’s pre-endosymbiont hypothesis: All symbiotic models are wrong and there is no alternative to gradualism at eukaryote origin. The authors should show some guts and apply the same reasoning they use here to the origin of plastids and then see if they are not confronted with a siutualtion in which plastids should be arising all the time from cyanobacteria because there are so many advantages. Or maybe they could explian what special propoerty the host lineage that acquired the plastid bneeded to evolve in order to acquire a cyanobacterial plastid (while other eukaryotes did not). The fact is (I never use the word fact) that the gradualist mechanisms and reasoning they envoke here do not apply to endosymbiosis. Some major transitions in evolution are not gradual in nature. Some symbiotic theories add extra symbints to ease the transition from prokaryotes to eukaryotes. Let people believe what they want to belive, but when reporting on the literature, please get it right.

***Cited references (local to our responses)*** *:*

1. *Maynard Smith J, Szathmáry E (1995) The major transitions in evolution. Oxford: Freeman & Co., 360 pp.*
2. *Szathmáry E (2015) Toward major evolutionary transitions theory 2.0. Proceedings of the National Academy of Sciences of the United States of America 112: 10104–10111.*
3. *Cavalier-Smith T (2002) The phagotrophic origin of eukaryotes and phylogenetic classification of Protozoa. International Journal of Systematic and Evolutionary Microbiology 52: 297–354.*
4. *Jékely G (2007) Origin of phagotrophic eukaryotes as social cheaters in microbial biofilms. Biology Direct 2.*
5. *Yutin N, Wolf MY, Wolf YI, Koonin E (2009) The origins of phagocytosis and eukaryogenesis. Biology Direct 4.*
6. *Koonin EV, Yutin N (2014) The dispersed archaeal eukaryome and the complex archaeal ancestor of eukaryotes. Cold Spring Harbor Perspectives in Biology 6.*
7. *Keeling PJ (2010) The endosymbiotic origin, diversification and fate of plastids. Philosophical Transactions of the Royal Society of London B: Biological Sciences 365: 729–748.*
8. *Maruyama S, Kim E (2013) A modern descendant of early green algal phagotrophs. Current Biology 23: 1081 - 1084.*
9. *Gagat P, Mackiewicz P (2017) Cymbomonas tetramitiformis - a peculiar prasinophyte with a taste for bacteria sheds light on plastid evolution. Symbiosis 71: 1–7.*
10. *Powell MJ (1984) Fine structure of the unwalled thallus of rozella polyphagi in its host polyphagus euglenae. Mycologia 76: 1039-1048.*
11. *Karpov SA, Mikhailov KV, Mirzaeva GS, Mirabdullaev IM, Mamkaeva KA, et al. (2013) Obligately phagotrophic aphelids turned out to branch with the earliest-diverging fungi. Protist 164: 195–205.*
12. *James TY, Pelin A, Bonen L, Ahrendt S, Sain D, et al. (2013) Shared signatures of parasitism and phylogenomics unite cryptomycota and microsporidia. Current Biology 23: 1548–1553.*
13. *Letcher PM, Longcore JE, Quandt CA, da Silva Leite D, James TY, et al. (2017) Morphological, molecular, and ultrastructural characterization of rozella rhizoclosmatii, a new species in cryptomycota. Fungal Biology 121: 1–10.*
14. *Dey G, Thattai M, Baum B (2016) On the archaeal origins of eukaryotes and the challenges of inferring phenotype from genotype. Trends in Cell Biology 26: 476–485.*
15. *Koonin EV (2010) The origin and early evolution of eukaryotes in the light of phylogenomics. Genome Biology 11: 209.*
16. *Számadó S, Szathmáry E (2006) Selective scenarios for the emergence of natural language. Trends in Ecology and Evolution 21: 555–561.*

### Reviewer #3: Purificación López-García

#### Reviewer recommendations

This manuscript critically examines current models for the origin of mitochondria during eukaryogenesis around twelve elements or questions that any eukaryogenetic hypothesis should try to account for: six present-day observables and six historical inferences. The authors find that no single hypothesis explains satisfactorily all the points and urge for new models that accommodate those observables and inferences well. This is a laudable objective that should stimulate the proposal of better-elaborated or refined models providing increasing levels of detail for all these points. The discussion is rich and insightful at some points, more naïve or simplistic and possible subjective at others. At any rate, this discussion is interesting and much welcome.

***Author’s answer:*** *We are grateful for Purificación López-García for her review, especially for the invaluable comments and corrections.*

Given the variety of elements provided, a point-by-point treatment would be too long. I will only highlight a few points:

Perhaps my most important concern regarding this manuscript relates to the lack of a real ecological perspective, which is absolutely required for eukaryogenetic models (but most often ignored). The authors use the term “ecology” but in a very restrictive way; they refer to the biotic interactions between the mitochondrial ancestor and its host. However, the fate of any symbiotic relationship, and most particularly of metabolic symbioses, depends on the environment. The environmental context is absent from this debate, despite being crucial. The authors may not want to enter in this discussion, given that most models don’t, but then I suggest that they simply talk of ‘biotic interactions’, not ‘ecology’. Ecology implies biotic and abiotic interactions, the latter are not considered here.

*In our understanding (due to our theoretical biologist background), ecology is about the dynamical coexistence (or extinction) of different species in local space and time. Nevertheless, López-García is right in that we deliberately ignored environmental factors relevant in endosymbiogenesis. As a matter of fact, we actually removed a section of our manuscript about the environmental background of the transition, realizing that it would take up too much space and would constitute its own review – which we humbly leave for those more experienced in geochemical processes. To make it clear, we have included explicit wording about the biotic and abiotic factors and an explanatory sentence at the end of the Results section.*

The authors reason that because membrane remodelling and cytoskeletal functions are present in archaea, early phagocytosis is supported. However, the link is not that straightforward, since archaea apparently lack phagocytosis even if they can remodel, and in some instances even fuse, their membranes. Those elements are therefore necessary but not sufficient for phagocytosis.

*We were indeed too enthusiastic in our expressions about archaeal phagocytosis: of course we only meant to refer to recent findings about the archaeal homologs of ESP-s. We have corrected statements about assumed archaeal (and LECA) phagocytosis.*

Phylogenomic analyses to unravel old relationships are to be taken with caution. The authors give credit to analyses suggesting a rickettsial ancestry for mitochondria and, based partly on this, favour a parasitic origin of mitochondria. However, that affinity is far from solid given potential problems in phylogenetic reconstruction derived of compositional biases and long-branch attraction (rickettsiales and mitochondria being prone to it because fast-evolving). Increasing the alphaproteobacterial sampling in these analyses can possibly lead to some improvement.

*We actually rely more on analyses not supporting direct rickettsial ancestry; we write under section Non-photosynthetic mitochondria: “most recent analyses exclude […] obligate endosymbiotic parasites of the Rickettsiales from the direct ancestry of mitochondria [Degli-Esposti et al. 2014].” (also discussed in section Additional file* 1 *: S1. Symbiont). Nevertheless, we have modified a sentence in the Conclusions to indicate that phylogenetic association is not a strong argument for parasitic origin.*

**Lokiarchaeota**. The authors may want to refer to the Asgard archaea collectively, since there are other TACK-like archaeal lineages that seem to share more genes with eukaryotes (Zaremba-Niedzwiedzka et al., Nature 2017). Also, the fact that Lokiarchaeota might use hydrogen does not necessarily support the hydrogen hypothesis (page 7, line 10). The syntrophy hypothesis, for instance, is equally based in interspecies hydrogen transfer.

*We’ve included references to Asgard archaea and added the syntrophy hypothesis as one that is also supported by a possible hydrogen-dependent extant archaeon.*

**Page 7, lines 48–54.** Here, the authors claim that metabolic complementarity is not enough to establish syntrophy. I agree in principle. However, this is where the ecological part is missing in this manuscript and where it would be important to consider these interactions in natural ecosystems. Some syntrophic models clearly specify metabolic interactions in plausible environmental contexts. Furthermore, the metabolic interactions proposed are based in actual syntrophies occurring in oxygen-depleted environments (for a detailed review see Lopez-Garcia et al., J Theor Biol, 2017). The assertion that “To this day, no case is known where a syntrophic relationship was turned to endosymbiosis” (again mentioned in page 9, lines 38–40) is not true. This has happened many times in protists. It is arguably rare in prokaryotes, but… eukaryotes evolved only once.

*We have corrected the sentence to read: “To this day, no case is known where a syntrophic relationship* ***among prokaryotes*** *was turned to* ***obligate*** *endosymbiosis […]”.*

**Page 8, lines 44–46.** The authors favour parasitic scenarios of eukaryogenesis because, in their opinion, conservative vertical transmission of proto-symbionts in syntrophic scenarios is hard to explain. This claim is not substantiated. Syntrophic scenarios provide a flexible historical path in an ecologically meaningful context whereby, in the beginning, interactions are facultative. Under those conditions, neither vertical conservation nor eukaryogenesis occur. However, at a given point, syntrophy becomes obligatory (this is likely stabilized by transfer of essential genes from the symbiont to the host genome and lost from the donor) and at some point eukaryogenetic. Under this situation, natural selection imposes vertical transmission, as the survival of the consortium in their precise ecological context depends on the two partners.

*We strongly believe that if two partners do not engage in intracellular contact (digestion, parasitism, whatever), there would be no way of a major transition to happen. A (now) classical objection is from Cavalier-Smith [1]: “extracellular syntrophy, sometimes postulated (Martin & Müller 1998), would not have helped; carrier insertion would probably not be mechanistically possible—if it occurred it would be disadvantageous by extruding proteobacterial metabolites into the environment, not the host”. Unless someone proves the possibility of this idea among prokaryotes (via experimentation or modelling), we remain sceptic. Furthermore, unless partners are so strongly coupled that their co-reproduction and co-inheritance is ensured, selection cannot act on the partnership as a new unit of evolution. In syntrophic scenarios, partners can come and go freely and are not linked with various hosts – if there is a host at all and not just a loose bunch of various syntrophic partners of equal rank/size colonizing a resource together. Syntrophic consortia should divide “together” so that their selective advantage is heritable. Also, assuming an initially mutual relationship does not explain how the host controlled possible parasites: partners that did not provide anything in exchange for the host’s resources.*

In their supplementary discussion, the authors discard models proposing an endosymbiotic origin of the nucleus because in addition of requiring two steps (two endosymbioses), phylogenomic analyses do not support a third, major genome donor apart of the archaeal and proteobacterial sources. The endosymbiotic origin of the nucleus is not without problems, but these are not the good arguments against it. First, multiple metabolic symbioses are extremely frequent in the microbial world (see e.g. Lopez-Garcia et al., J Theor Biol 2017 and references therein) and parsimony does not necessarily work in evolution. Second, phylogenomic analyses do indeed reveal additional discernible bacterial heritage to that of Alphaproteobacteria in eukaryotic genomes, including deltaproteobacteria, planctomycetes or actinobacteria (Pittis & Gabaldon, Nature 2026).

*While there is certainly a non-alphaproteobacterial component to the eukaryotic genome, we strongly believe that this is a result of strong horizontal gene transfer before the nucleus evolved and the genome was stabilized. And we strongly believe that the source of this HGT was either parasitic or phagocytotic. While we cannot prove any of these (hence we have ignored to discuss them in detail) one also cannot prove (or disprove) the source to be a third endosymbiotic partner for the moment. Also, we believe in the endogenous origin of the nucleus, as being part of the eukaryotic endomembrane system – but discussing this issue above mere opinions would increase the manuscript beyond limits. Nevertheless, we have included reference about multiple metabolic symbioses being frequent.*

The authors also dismiss the possibility of methanogenesis as energetic metabolism for the archaeon based on the required strict anaerobiosis and on the fact that methanogenesis-related genes are absent from eukaryotes and that archaea-related eukaryotic genes do not resemble those of classical methanogens. However, the authors need to separate the metabolism of the archaeal ancestor of eukaryotes and that of the evolving methanogenic consortium. It might well be that another kind of anaerobic metabolism involving e.g. fermentation occurred at the origin of eukaryotes, but the anaerobiosis transition to aerobiosis must have represented the same problem. Both the hydrogen and the syntrophy models imply a transition from the methanogenic consortium involving a facultative aerobic partner (future mitochondrion) to an aerobic consortium that abandons methanogenesis in favour of a much more efficient aerobic respiration. Both models predict the same: methanogenesis is lost. Consequently, it is not at all surprising that methanogenesis related genes are missing in eukaryotes. The contrary would be surprising. Finally, although archaeal-related genes in eukaryotes seem to resemble more TACKL-archaea genes that those of classical euryarchaeotal methanogens, it is now clear that at least Bathyarchaeota, on the TACKL side, do have methanogenesis genes. This implies that the ancestor of archaea was likely a methanogen and leaves open the possibility of a methanogenesis-based consortium at the onset of eukaryogenesis; methanogenesis being subsequently fully lost.

*From the viewpoint of parsimony, we believe that it is more probable that what is missing in eukaryotes (clues of ancient methanogenic metabolism) was not there ab initio. Phylogenomic data do not support methanogenic metabolism, though many ancestral cues of the same era can be traced within the eukaryotic genome, indicating that signals of once-metabolism can possibly survive till today. Furthermore, no eukaryote has returned to methanogenesis.*

Table 2 **.** The “(untenable)” in the syntrophy hypothesis case is not justified in light of the above. The syntrophy hypothesis, as the rest of the models, can be criticized in many ways but from an ecological and evolutionary perspective, the kind of ab-initio metabolic consortium proposed makes sense. Methanogenesis itself is somehow irrelevant because this metabolism is subsequently lost. The same is true for any other kind of anaerobic metabolism, since the ancestor of eukaryotes was an aerobic heterotroph.

*It is right that the transition from anaerobic to aerobic must be answered by all hypotheses, but the present formulation of the hydrogen hypothesis (e.g. in [2]) ultimately dropped the methanogenic consortia from the initial setup, emphasizing that the hypothesis only requires strict H*_*2*_ *-dependency from the host, any kind will do. While in the syntrophic hypothesis, the first endosymbiotic event, giving rise to the nucleus, specifically depends on a methanogenic consortium. “It is true, that this step precedes the integration of the mitochondria, and one can independently evaluate the latter, but according to the latest formulation [3], the early mitochondrion relied on the host’s methane. We have corrected the statement to ‘methanogenic metabolism untenable’.”*

**Cited references (local to our responses)**:

1. *Cavalier-Smith T (2006) Origin of mitochondria by intracellular enslavement of a photosynthetic purple bacterium. Proceedings of the Royal Society of London B: Biological Sciences 273: 1943–1952.*
2. *Martin WF, Garg S, Zimorski V (2015) Endosymbiotic theories for eukaryote origin. Philosophical Transactions of the Royal Society of London B: Biological Sciences 370: 1–18.*
3. *López-García P, Moreira D (2006) Selective forces for the origin of the eukaryotic nucleus. BioEssays 28: 525–533.*

### Reviewer #2, 2nd revision: William Martin

1. On page 9 the paper now states that Mereschkowsky proposed a symbiotic origin [of mitochonrra in the context of the sentence], but of course he never suggested a symbiotic origin of mitochondria as I have pointed out in many of the papers cited here. Mereschkowsky never suggested an endosymbitic origin of mitochondria (nor did Altmann), so there is a big error in the second sentence of the conclusions.

*We have modified the sentence to make it more clear for the Reader.*

2. **p. 9 line 44,** there is a theory out there where host and symbiont are both bacteria and all of the archaeal genes in eukayrotes wereacquzured via lateral gene transfer, it was from the Nobel laureate Christian de Duve 2007 (ref [38] in this version). That needs to be corrected, and a carefuzl read of de Duve would be required to make sure that he was not suggesting syntrophy.. The reason that de Duve went to such evolutionary acrobatics as to suggest that eukaryotes acquired their cvytosolic ribosomes via LGT is because he had to make sure that hydrogenosomes had no evolutionary significance.

*De Duve in 1969 (Annals of the New York Academy of Sciences 168(2)) explicitly refers to a “primitive phagocyte” that acquired mitochondria. Later on in 2007 (Nature Reviews Genetics 8(5)) he also assumes a phagocytic mechanism in the eubacterium-related host (p.396-397) and states very clearly his standpoint: “Eukaryotic cells most probably acquired mitochondria after they had developed the cytomembrane and cytoskeletal machineries that are involved in the endocytic uptake of extracellular materials, and not before” (p.401). Of course, the ability of phagocytosis per se does not exclude any concurrent syntrophic relationship (we also believe that these were of serious importance), but the* ***mechanism of inclusion*** *must have been either slow syntrophic engulfment or phagocytic capture (or parasitic invasion) – and not a mixture of these. Anyway, thank you for pointing out that we have missed to include his theory in Table S1, it is added now.*

3. On page 7, they contradict themselves (or maybe they don’t understand that it is the same issue). On line 14 they say that they (or most everyone, per convergence) believe that eukarypte to eukaryote LGT is the mechanism speading the anaerobic lifestyle among eukaryotes, which I think is really wrong (and which Ku et al. 2015, [6] tested and rejected, OR they think that eukaryotes acquire their genes for the anaerobic lifestyle from prokaryotes, which Ku et al. 2016 (BMC Evol Biol, not cited here) also tested and rejected. But on the same page line 48 they say that the symbiont was a facultative anaerobe, which is what the data do in fact say ind which is what Martin and Müller said in 1998. So it is very had to see what the authors think.

*We certainly do not believe that the eukaryote to eukaryote LGT is the mechanism spreading the anaerobic lifestyle among eukaryotes. We have clarified and corrected this sentence (in section* ***Symbiont metabolism)*** *to better conform to results and added the references.*

### Rewiever #3, 2nd revision: Purificación López-García

The manuscript by Zachar and Szathmáry has been improved and deserves publication. However, I would still like to comment on a few points.

I understand that the authors do not want to enter in the discussion of the abiotic factors involved in the selection of particular symbiotic consortia because it will lengthen their manuscript. They say they ignore abiotic conditions to concentrate in the symbiotic interactions. The problem is that metabolic symbiotic interactions do depend on the environmental setting and it is not possible to understand the former without taking into account the latter. Ecology, as they say, has to do with the study of the "dynamical coexistence (or extinction) of different species in local space and time". But in the real material world where biology thrives and where eukaryotes evolved, ‘space’ implies the biotope, the natural setting imposing a variety of abiotic constraints, plus the interactions (cooperative, competitive, neutral) with the rest of the biological community. The lack of a true (microbial) ecological perspective prevents the authors to fully understand some hypotheses, leading to some incorrect or simplistic assumptions. They have an excessively fixed, theoretical view that does not consider metabolic flexibility within microbes (“facultative” metabolism operating under varying environmental conditions) and flexibility of symbiotic interactions depending on the environmental conditions (typically syntrophies, and symbioses in general, may be obligatory or not depending on the environment). Yet, this is crucial to understand how eukaryotes evolved from one (or several) metabolic symbiosis. Zachar and Szathmáry argue against a methanogenic archaeon at the origin of eukaryotes because methanogenesis genes are not found in eukaryotes, and they imagine that at least some remnants should be found in eukaryotic genomes. It is funny that they acknowledge in their manuscript the possibility to fully lose photosynthesis (and its genes), but not methanogenesis (and its genes)... However, we do know that methanogenesis has been lost several times independently in different archaeal branches (e.g. haloarchaea, Thermococcales, and most likely most TACKL lineages) and with it, the full set of methanogenesis genes. This is only natural from a microbial ecology and evolution perspective. Prokaryotic genomes are streamlined and, if you do not use a function (methanogenesis, nitrogen fixation, photosynthesis, etc.), you easily lose the corresponding genes. In many cases, it is possible to gain genes back by horizontal gene transfer whenever needed, and this often happens in natural environments provided the appropriate selective pressure appears (e.g. antibiotic resistance, degradation of complex compounds; although no case of horizontal gain of methanogenesis is known). So, it is only logical that complete loss of methanogenesis genes happened at the origin of eukaryotes should a eukaryogenic methanogenic consortia stabilize advantageous metabolic interactions with a facultative aerobic alphaproteobacterium that provided better energetic yields to the consortium in the presence of oxygen (as the syntrophy hypothesis states).

*We do acknowledge the importance of abiotic factors and also strongly believe in the early importance of flexible metabolism of host and symbiont. We even mention this as a possibility under Host metabolism (“One can assume that the host had a mixed metabolism, autotrophic in light, heterotrophic in dark”). We also admit that there are very few metabolic combinations that no microbe performs at all.*

*Losing photosynthesis without a trace (though we mention cristae as possible remnants) is indeed analogous to losing methanogenesis without a trace – thank you for pointing out our logical inconsistency. We certainly don’t want to imply that the lack of evidence in the microbial world is proof of absence. What we stated in our response was: “it is more probable that what is missing in eukaryotes […] was not there ab initio”, and you are right that this should equally apply to methanogenesis and photosynthesis. On the other hand, we never stated that the ancestral symbiont was certainly primarily photosynthetic: most of the discussed theories assume a primarily non-photosynthetic symbiont (Additional file* 1 *: Table S2), which of course means that lacking photosynthetic genes are primarily lacking and not lost. We admit, that the lack of methanogenesis can be a primary or secondary trait, but the line in Additional file* 1 *: Table S2 however (“missing genes of methanogenic metabolism in the eukaryotic genome”), is a fact.*

Talking about the syntrophy hypothesis, Zachar and Szathmáry say that the proposed metabolism within the eukaryogenic consortium is untenable because methanogenesis requires strict anaerobic conditions and the alphaproteobacterial ancestor of mitochondria at some point starts to respire oxygen. They say “It is true, that this step precedes the integration of the mitochondria, and one can independently evaluate the latter, but according to the latest formulation [3]”, the early mitochondrion relied on the host’s methane. We have corrected the statement to “methanogenic metabolism untenable”. I think they severely misunderstand the syntrophy hypothesis, which is possibly the most detailed model from a microbial ecology point of view. The syntrophy hypothesis proposes an evolving syntrophic consortium along time and across redox gradients. The ancestor of mitochondria is a versatile (able to shift between different energy metabolism modes depending on the environment) alphaproteobacterium that is facultatively aerobic (= respires oxygen when it is available and entertains other energy metabolic reactions when oxygen is missing) and also methanotrophic (being able to oxidize methane when oxygen is not present). This alphaproteobacterium (not yet an early mitochondrion, as Zachar and Szathmáry incorrectly imply) establishes a symbiosis with an existing consortium of a deltaproteobacterial ancestor of myxobacteria and a methanogen. Metabolic exchanges occur in anoxic conditions. However, the consortium can experience shifts in the redox gradient and get exposed to more oxygenated areas (as many facultative aerobes today in freshwater and marine sediments). Then, oxygenic respiration takes place. Under very low oxygen pressure, it might still be possible that methanogenesis still operates if the alphaproteobacterium removes all the oxygen reaching the consortium via aerobic respiration. However, what the syntrophy hypothesis very clearly states is that at a given transition point, the consortium evolves to retain only the much more efficient oxygen respiration (which by the way also opens a panoply of new ecological niches for the eukaryogenic consortium). In this situation, methanogenesis is totally inhibited by oxygen, completely useless for the consortium and fully lost with all its genes (as it has repeatedly been fully lost in many archaea, including aerobic archaea). At that particular point, we could talk of an early mitochondrion; it would be a facultative aerobic organelle in a symbiotic consortium that has fully lost methanogenesis (and along with it the archaeal membrane where many methanogenesis enzymes lie). Even if some residual methanotrophic activity might potentially still be present in the mitochondrion, methane would no longer come from the host, but occasionally from the environmental setting. Actually, under the syntrophy hypothesis, one key eukaryogenetic step (because it triggers severe cellular changes) is the loss of the methanogenesis subsequent to the permanent shift to oxygen-respiration by the alphaproteobacterium. Until then, we only had a symbiotic consortium; from here onwards we have a true proto-eukaryote and an early mitochondrion.

*We accept this scenario as a possible evolutionary route, and have added a paragraph explaining it in more detail under Additional file* 1 *: S4. 7 Host metabolism (and removed the “untenable” statement from Table* *2* *of the main text).*

Zachar and Szathmáry say that myxobacterial genes are absent from eukaryotes. However, this is also incorrect. Myxobacteria share several features with eukaryotes (see references in Moreira & Lopez-Garcia 1998, Lopez-Garcia and Moreira 2006), myxobacterial genes are involved in fatty acid oxidation in eukaryotes (Schluter et al., PLoS ONE 2011) and, in more general terms, there is a deltaproteobacterial signal in eukaryotes (Hug et al., Mol Biol Evol 2010, Pittis et al. Nature 2016). These genes might derive from other kind of horizontal gene transfers and not necessarily from a symbiotic event; yet they are there.

*We never intended to state that there is no deltaproteobacterial (or specifically myxobacterial) signal in the eukaryote genome – there is. We cannot find the incriminated passage in the manuscript to which López-García refers to.*

## Additional file

Additional file 1: Supplemental Information includes the detailed description and extensive analysis of hypotheses, one figure, and three tables and can be found with this article online at TBA. (DOCX 258 kb)

## Abbreviations

ANT: Adenine nucleotide translocator, ATP/ADP translocase
ETC: Electron transport chain
FECA: First eukaryal common ancestor, i.e. the initial ancestor of the eukaryotic lineage
HGT: Horizontal gene transfer
LECA: Last eukaryal common ancestor, i.e. common ancestor of all extant Eukaryota
MRO: Mitochondrion-related organelles (anaerobic mitochondria, hydrogenosomes and mitosomes)
TACK: Thaumarchaeota, Aigarchaeota, Crenarchaeota (eocytes), Korarchaeota

## References

[1] Maynard Smith J, Szathmáry E (1995). The major transitions in evolution.

[2] Blackstone NW (2013). Why did eukaryotes evolve only once? Genetic and energetic aspects of conflict and conflict mediation. Philos Trans R Soc Lond B: Biol Sci..

[3] Szathmáry E (2015). Toward major evolutionary transitions theory 2.0. Proc Natl Acad Sci U S A.

[4] López-García P, Moreira D (2015). Open questions on the origin of eukaryotes. Trends Ecol Evol.

[5] McInerney JO, O’Connell MJ, Pisani D (2014). The hybrid nature of the Eukaryota and a consilient view of life on earth. Nat Rev Microbiol.

[6] Ku C, Nelson-Sathi S, Roettger M, Sousa FL, Lockhart PJ (2015). Endosymbiotic origin and differential loss of eukaryotic genes. Nature.

[7] Pittis AA, Gabaldón T (2016). Late acquisition of mitochondria by a host with chimaeric prokaryotic ancestry. Nature.

[8] Martin WF, Neukirchen S, Zimorski V, Gould SB, Sousa FL (2016). Energy for two: new archaeal lineages and the origin of mitochondria. BioEssays.

[9] Gray MW (2015). Mosaic nature of the mitochondrial proteome: implications for the origin and evolution of mitochondria. Proc Natl Acad Sci.

[10] López-García P, Eme L, Moreira D. Symbiosis in eukaryotic evolution. J Theoretical Biol. 2017. In press. 10.1016/j.jtbi.2017.02.031 PMC5638015 28254477

[11] Keeling PJ, Koonin EV. Origin and evolution of eukaryotes. Cold Spring Harbor Perspectives in Biology. Cold Spring Harbor Laboratory Press; 2014. p. 416. https://www.cshlpress.com/default.tpl?cart=140992238322770318&fromlink=T&linkaction=full&linksortby=oop_title&--eqSKUdatarq=1010.

[12] Martin WF, Garg S, Zimorski V (2015). Endosymbiotic theories for eukaryote origin. Philos Trans R Soc Lond B Biol Sci..

[13] Lane N, Martin W (2010). The energetics of genome complexity. Nature.

[14] Mentel M, Martin W (2008). Energy metabolism among eukaryotic anaerobes in light of Proterozoic ocean chemistry. Philos Trans R Soc Lond B Biol Sci..

[15] Poole AM, Gribaldo S. Eukaryotic origins: how and when was the mitochondrion acquired? Cold Spring Harb Perspect Biol. 2014;6. 10.1101/cshperspect.a015990 PMC4292153 25038049

[16] Martijn J, Ettema TJG (2013). From archaeon to eukaryote: the evolutionary dark ages of the eukaryotic cell. Biochem Soc Trans.

[17] Spang A, Saw JH, Jorgensen SL, Zaremba-Niedzwiedzka K, Martijn J (2015). Complex archaea that bridge the gap between prokaryotes and eukaryotes. Nature..

[18] Ettema TJG (2016). Mitochondria in the second act. Nature.

[19] Williams TA, Embley TM. Changing ideas about eukaryotic origins. Philos Trans R Soc Lond B Biol Sci. 2015;370(1678):20140318. 10.1098/rstb.2014.0318 PMC4571560 26323752

[20] Degli Esposti M (2016). Late mitochondrial acquisition, really?. Genome Biol Evol.

[21] Koonin EV (2010). The origin and early evolution of eukaryotes in the light of phylogenomics. Genome Biol.

[22] Koumandou VL, Wickstead B, Ginger ML, van der Giezen M, Dacks JB (2013). Molecular paleontology and complexity in the last eukaryotic common ancestor. Crit Rev Biochem Mol Biol.

[23] Ball SG, Bhattacharya D, Weber APM (2016). Pathogen to powerhouse. Science.

[24] Számadó S, Szathmáry E (2006). Selective scenarios for the emergence of natural language. Trends Ecol Evol.

[25] Koonin EV, Yutin N (2014). The dispersed archaeal eukaryome and the complex archaeal ancestor of eukaryotes. Cold Spring Harb Perspect Biol.

[26] Williams TA, Embley TM (2014). Archaeal “dark matter” and the origin of eukaryotes. Genome Biol Evol.

[27] Guy L, Saw JH, Ettema TJ (2014). The archaeal legacy of eukaryotes: a phylogenomic perspective. Cold Spring Harb Perspect Biol.

[28] Raymann K, Brochier-Armanet C, Gribaldo S (2015). The two-domain tree of life is linked to a new root for the archaea. Proc Natl Acad Sci.

[29] Lang BF, Burger G. Mitochondrial and eukaryotic origins: a critical review. In: Advances in Botanical Research, volume 63. Amsterdam: Academic press, Elsevier; 2012. p. 1–20.

[30] Rodríguez-Ezpeleta N, Embley TM (2012). The SAR11 group of alpha-proteobacteria is not related to the origin of mitochondria. PLoS One.

[31] Embley TM, Martin W (2006). Eukaryotic evolution, changes and challenges. Nature.

[32] Müller M, Mentel M, van Hellemond JJ, Henze K, Woehle C (2012). Biochemistry and evolution of anaerobic energy metabolism in eukaryotes. Microbiol Mol Biol Rev.

[33] Koonin EV (2015). Archaeal ancestors of eukaryotes: not so elusive any more. BMC Biol.

[34] Cavalier-Smith T (1987). The origin of eukaryote and archaebacterial cells. Ann N Y Acad Sci.

[35] Forterre P (2011). A new fusion hypothesis for the origin of Eukarya: better than previous ones, but probably also wrong. Res Microbiol.

[36] Cavalier-Smith T (2006). Origin of mitochondria by intracellular enslavement of a photosynthetic purple bacterium. Proc R Soc Lond B Biol Sci.

[37] Cavalier-Smith T (2014). The neomuran revolution and phagotrophic origin of eukaryotes and cilia in the light of intracellular coevolution and a revised tree of life. Cold Spring Harb Perspect Biol.

[38] de Duve C (2007). The origin of eukaryotes: a reappraisal. Nat Rev Genet.

[39] Sagan L (1967). On the origin of mitosing cells. J Theor Biol.

[40] Whatley JM, John P, Whatley FR (1979). From extracellular to intracellular: the establishment of mitochondria and chloroplasts. Proc R Soc Lond B Biol Sci.

[41] Gray MW (2014). The pre-endosymbiont hypothesis: a new perspective on the origin and evolution of mitochondria. Cold Spring Harb Perspect Biol..

[42] Koonin EV (2015). Origin of eukaryotes from within archaea, archaeal eukaryome and bursts of gene gain: eukaryogenesis just made easier?. Philos Trans R Soc London B Biol Sci..

[43] Yutin N, Koonin EV (2012). Archaeal origin of tubulin. Biol Direct.

[44] Martin WF, Tielens AGM, Mentel M, Garg SG, Gould SB (2017). The physiology of phagocytosis in the context of mitochondrial origin. Microbiol Mol Biol Rev.

[45] Martin W, Müller M (1998). The hydrogen hypothesis for the first eukaryote. Nature.

[46] Searcy DG (1992). Origins of mitochondria and chloroplasts from sulfur-based symbioses.

[47] Lane N, Martin W (2012). The origin of membrane bioenergetics. Cell.

[48] López-García P, Moreira D (2006). Selective forces for the origin of the eukaryotic nucleus. BioEssays.

[49] Degli Esposti M (2014). Bioenergetic evolution in proteobacteria and mitochondria. Genome Biol Evol.

[50] Lane N (2011). Energetics and genetics across the prokaryote-eukaryote divide. Biol Direct.

[51] Lane N. Bioenergetic constraints on the evolution of complex life. Cold Spring Harb Perspect Biol. 2014;6. 10.1101/cshperspect.a015982 PMC3996473 24789818

[52] Booth A, Doolittle WF (2015). Eukaryogenesis, how special really?. Proc Natl Acad Sci.

[53] Al Mamun M, Albergante L, Moreno A, Carrington JT, Blow JJ (2016). Inevitability and containment of replication errors for eukaryotic genome lengths spanning megabase to gigabase. Proc Natl Acad Sci.

[54] Lynch M, Marinov GK (2015). The bioenergetic costs of a gene. Proc Natl Acad Sci.

[55] Lynch M, Marinov GK. Membranes, energetics, and evolution across the prokaryote-eukaryote divide. Elife. 2017;6:e20437. 10.7554/eLife.20437 PMC5354521 28300533

[56] Davidov Y, Jurkevitch E (2009). Predation between prokaryotes and the origin of eukaryotes. BioEssays.

[57] Searcy DG (2003). Metabolic integration during the evolutionary origin of mitochondria. Cell Res.

[58] Lang BF. Mitochondria and the origin of eukaryotes. Vienna: Springer; 2014. chapter 1. p. 3–18. doi:10.1007/978-3-7091-1303-5. http://dx.doi.org/10.1007/978-3-7091-1303-5.

[59] Ettema TJG, Lindås AC, Bernander R (2011). An actin-based cytoskeleton in archaea. Mol Microbiol.

[60] Hartman H, Fedorov A (2002). The origin of the eukaryotic cell: a genomic investigation. Proc Natl Acad Sci.

[61] Zaremba-Niedzwiedzka K, Caceres EF, Saw JH, Bäckström D, Juzokaite L (2017). Asgard archaea illuminate the origin of eukaryotic cellular complexity. Nature.

[62] Čuboňová L, Sandman K, Hallam SJ, DeLong EF, Reeve JN (2005). Histones in Crenarchaea. J Bacteriol.

[63] Nunoura T, Takaki Y, Kakuta J, Nishi S, Sugahara J (2011). Insights into the evolution of archaea and eukaryotic protein modifier systems revealed by the genome of a novel archaeal group. Nucleic Acids Res.

[64] Godde JS (2012). Breaking through a phylogenetic impasse: a pair of associated archaea might have played host in the endosymbiotic origin of eukaryotes. Cell & Bioscience.

[65] López-García P, Moreira D (1999). Metabolic symbiosis at the origin of eukaryotes. Trends Biochem Sci.

[66] Lombard J, López-García P, Moreira D (2012). The early evolution of lipid membranes and the three domains of life. Nat Rev Microbiol.

[67] Gould SB, Garg SG, Martin WF (2016). Bacterial vesicle secretion and the evolutionary origin of the eukaryotic endomembrane system. Trends Microbiol.

[68] Andersson SGE, Kurland CG (1999). Origins of mitochondria and hydrogenosomes. Curr Opin Microbiol.

[69] Kurland CG, Andersson SGE (2000). Origin and evolution of the mitochondrial proteome. Microbiol Mol Biol Rev.

[70] Cavalier-Smith T (2006). Cell evolution and earth history: stasis and revolution. Philos Trans R Soc Lond B Biol Sci.

[71] Jékely G. Origin of eukaryotic endomembranes: a critical evaluation of different model scenarios. In: Advances in Experimental Medicine and Biology, vol. 607. New York: Springer; 2007. p. 38–51. doi:10.1007/978-0-387-74021-8_3. 10.1007/978-0-387-74021-8_3. 10.1007/978-0-387-74021-8_3 17977457

[72] Diekmann Y, Pereira-Leal JB (2013). Evolution of intracellular compartmentalization. Biochem J.

[73] Cavalier-Smith T (2013). Symbiogenesis: mechanisms, evolutionary consequences, and systematic implications. Annu Rev Ecol Evol Syst.

[74] Woese CR (1977). Endosymbionts and mitochondrial origins. J Mol Evol.

[75] Brindefalk B, Ettema TJG, Viklund J, Thollesson M, Andersson SGE (2011). A phylometagenomic exploration of oceanic alphaproteobacteria reveals mitochondrial relatives unrelated to the SAR11 clade. PLoS One.

[76] Degli Esposti M, Chouaia B, Comandatore F, Crotti E, Sassera D (2014). Evolution of mitochondria reconstructed from the energy metabolism of living bacteria. PLoS One.

[77] Williams KP, Sobral BW, Dickerman AW (2007). A robust species tree for the Alphaproteobacteria. J Bacteriol.

[78] Wang Z, Wu M (2015). An integrated phylogenomic approach toward pinpointing the origin of mitochondria. Sci Rep.

[79] Degli Esposti M, Cortez D, Lozano L, Rasmussen S, Nielsen HB, et al. Alpha proteobacterial ancestry of the [Fe-Fe]-hydrogenases in anaerobic eukaryotes. Biol Direct. 2016;11 10.1186/s13062-016-0136-3 PMC4967309 27473689

[80] van der Giezen M (2009). Hydrogenosomes and mitosomes: conservation and evolution of functions. J Eukaryot Microbiol.

[81] Stairs CW, Leger MM, Roger AJ. Diversity and origins of anaerobic metabolism in mitochondria and related organelles. Philos Trans R Soc London B Biol Sci. 2015;370:1–13. 10.1098/rstb.2014.0326 PMC4571565 26323757

[82] Embley TM, van der Giezen M, Horner DS, Dyal PL, Foster P (2003). Mitochondria and hydrogenosomes are two forms of the same fundamental organelle. Philos Trans R Soc Lond B Biol Sci.

[83] van der Giezen M, Tovar J, Clark CG. Mitochondrion-derived organelles in protists and fungi. In: a survey of cell biology, academic press, volume 244 of International Review of Cytology. 2005. p. 175–225. doi:10.1016/S0074-7696(05)44005-X. http://www.sciencedirect.com/science/article/pii/S007476960544005X. 10.1016/S0074-7696(05)44005-X 16157181

[84] Baum DA, Baum B (2014). An inside-out origin for the eukaryotic cell. BMC Biol.

[85] Rochette NC, Brochier-Armanet C, Gouy M (2014). Phylogenomic test of the hypotheses for the evolutionary origin of eukaryotes. Mol Biol Evol.

[86] Sousa FL, Neukirchen S, Allen JF, Lane N, Martin WF. Lokiarchaeon is hydrogen dependent. Nat Microbiol. 2016;1. 10.1038/nmicrobiol.2016.34 27572645

[87] Ferla MP, Thrash JC, Giovannoni SJ, Patrick WM (2013). New rRNA gene-based phylogenies of the *Alphaproteobacteria* provide perspective on major groups, mitochondrial ancestry and phylogenetic instability. PLoS One.

[88] Sassera D, Lo N, Epis S, D’Auria G, Montagna M (2011). Phylogenomic evidence for the presence of a flagellum and cbb3 oxidase in the free-living mitochondrial ancestor. Mol Biol Evol.

[89] Wang Z, Wu M (2014). Phylogenomic reconstruction indicates mitochondrial ancestor was an energy parasite. PLoS One.

[90] Yutin N, Wolf MY, Wolf YI, Koonin E. The origins of phagocytosis and eukaryogenesis. Biol Direct. 2009;4. 10.1186/1745-6150-4-9 PMC2651865 19245710

[91] Gabaldón T, Huynen MA (2003). Reconstruction of the proto-mitochondrial metabolism. Science.

[92] Gabaldón T, Pittis AA (2015). Origin and evolution of metabolic sub-cellular compartmentalization in eukaryotes. Biochimie.

[93] Ku C, Martin WF (2016). A natural barrier to lateral gene transfer from prokaryotes to eukaryotes revealed from genomes: the 70% rule. BMC Biol.

[94] Muñoz-Gómez S, Slamovits C, Dacks J, Baier K, Spencer K (2015). Ancient homology of the mitochondrial contact site and cristae organizing system points to an endosymbiotic origin of mitochondrial cristae. Curr Biol.

[95] Nakayama T, Kamikawa R, Tanifuji G, Kashiyama Y, Ohkouchi N (2014). Complete genome of a nonphotosynthetic cyanobacterium in a diatom reveals recent adaptations to an intracellular lifestyle. Proc Natl Acad Sci U S A.

[96] Liu Z, Müller J, Li T, Alvey RM, Vogl K (2013). Genomic analysis reveals key aspects of prokaryotic symbiosis in the phototrophic consortium *Chlorochromatium aggregatum*. Genome Biol.

[97] Jeon KW (1972). Development of cellular dependence on infective organisms: micrurgical studies in amoebas. Science.

[98] Dey G, Thattai M, Baum B (2016). On the archaeal origins of eukaryotes and the challenges of inferring phenotype from genotype. Trends Cell Biol.

[99] van den Ent F, Amos LA, Löwe J (2001). Prokaryotic origin of the actin cytoskeleton. Nature.

[100] Szwedziak P, Wang Q, Freund SMV, Löwe J (2012). FtsA forms actin-like protofilaments. EMBO J.

[101] Jékely G. Origin and evolution of the self-organizing cytoskeleton in the network of eukaryotic organelles. Cold Spring Harb Perspect Biol. 2014;6. 10.1101/cshperspect.a016030 PMC4142967 25183829

[102] Amiri H, Karlberg O, Andersson GES (2003). Deep origin of plastid/parasite ATP/ADP translocases. J Mol Evol.

[103] Andersson GE, Karlberg O, Canbäck B, Kurland CG (2003). On the origin of mitochondria: a genomics perspective. Philos Trans R Soc Lond B Biol Sci.

[104] Mereschkowsky C (1910). Theorie der zwei Plasmaarten als Grundlage der Symbiogenesis, einer neuen Lehre von der Entstehung der Organismen. Biol Centralbl.

[105] Gould SB (2016). Infection and the first eukaryotes. Science.

[106] Martin WF, Roettger M, Ku C, Garg SG, Nelson-Sathi S, et al. Late mitochondrial origin is pure artefact. bioRxiv. 2016. http://dx.doi.org/10.1101/055368. 10.1093/gbe/evx027 PMC5516564 28199635

[107] Lynch M, Marinov GK (2016). Reply to lane and Martin: mitochondria do not boost the bioenergetic capacity of eukaryotic cells. Proc Natl Acad Sci.

[108] Cavalier-Smith T. The chimaeric origin of mitochondria: photosynthetic cell enslavement, gene-transfer pressure, and compartmentation efficiency. Berlin, Heidelberg: Springer; 2007. p. 161–99. doi:10.1007/978-3-540-38502-8_8. http://dx.doi.org/10.1007/978-3-540-38502-8_8.

[109] Martin W, Hoffmeister M, Rotte C, Henze K (2001). An overview of endosymbiotic models for the origins of eukaryotes, their ATP-producing organelles (mitochondria and hydrogenosomes), and their heterotrophic lifestyle. Biol Chem.

[110] Moreira D, López-García P (1998). Symbiosis between methanogenic archaea and δ-proteobacteria as the origin of eukaryotes: the syntrophic hypothesis. J Mol Evol.

[111] Searcy DG (2006). Rapid hydrogen sulfide consumption by *Tetrahymena pyriformis* and its implications for the origin of mitochondria. Eur J Protistol.

