# Supplementary material for: Breath-giving cooperation: critical review of origin of mitochondria hypotheses: Major unanswered questions point to the importance of early ecology
Source: Biol Direct. 2017 Aug 14;12:19. doi: 10.1186/s13062-017-0190-5 (PMC5557255; doi:10.1186/s13062-017-0190-5)
Supplement: Additional file 1: — Supplemental Information includes the detailed description and extensive analysis of hypotheses, one figure, and three tables and can be found with this article online at TBA. (DOCX 258 kb) [file 13062_2017_190_MOESM1_ESM.docx]

Breath-giving cooperation:
Critical review of origin of mitochondria hypotheses
Supplemental information

István Zachar^1,2^*, Eörs Szathmáry^1,2,3^

^1^ Eötvös Loránd University, Department of Plant Systematics, Ecology and Theoretical Biology, Pázmány P. sétány 1/C, Budapest 1117, Hungary

^2^ Evolutionary Systems Research Group, MTA, Centre for Ecological Research, Hungarian Academy of Sciences, Klebelsberg Kunó str. 3., Tihany 8237, Hungary

^3^ Parmenides Foundation, Kirchplatz 1, 82049 Pullach/Munich, Germany

* corresponding author

# Abbreviations

LECA: last eukaryal common ancestor, i.e. common ancestor of all extant Eukaryota

FECA: first eukaryal common ancestor, i.e. the initial ancestor of the eukaryotic lineage

HGT: horizontal gene transfer

TACK: Thaumarchaeota, Aigarchaeota, Crenarchaeota (eocytes), Korarchaeota

ROS: reactive oxygen species

ETC: electron transport chain

G1P/G3P: glyceraldehyde 1/3-phosphate

ESCRT: endosomal sorting complexes required for transport, a cytosolic protein complex involved in membrane remodelling

ANT: adenine nucleotide translocator, ATP/ADP translocase

PFO, PNO, PFL: pyruvate:ferredoxin oxidoreductase, pyruvate:NADP oxidoreductase, pyruvate formate lyase

MRO: mitochondrion-related organelles (anaerobic mitochondria, hydrogenosomes and mitosomes)

NPC: non-alphaproteobacterial component of the mitochondrial proteome

1. Partners

## Host

There is a wide variety of taxa postulated as initial host. Many suggested a bacterium (proteobacterium [1-4], a Planctomycetes [5], or a neomuran [6, 7]; see Table 1), but the archaeal host model [8-17] gains more and more support from phylogenomic analyses [18-25]. While results indicate that the host was an archaeon (or at least closer to the Archaea than to any bacteria), it is still debated, which exact group it belonged to (cf. [8, 26-32]) and what its metabolism was like. The eukaryotic origin from the archaeal TACK superphylum (comprising Thaumarchaeota, Aigarchaeota, Crenarchaeota and Korarchaeota) [9, 20-23] (consistent with an extended eocyte tree [16, 33] and not with the 3-domains tree [34-37]) is supported by proteins found in TACK members that were thought to be uniquely eukaryotic [8, 31, 38].

Most recently, Asgard archaea (a sister to Lokiarchaea [20]) was suggested as the closest relative of the host as its composite genome encodes the most homologs of eukaryotic signature proteins [39] of all sequenced prokaryotes [20] [40]. The ESCRT machinery in eukaryotes is involved in membrane remodelling of the nuclear envelope. It is not clear however, what is the purpose of its homologs in Archaea [40], or why it was retained (assuming secondary (thermo)reduction [32]). The real problem however with Asgard archaea (and Lokiarchaeota) is that no one has seen a living cell of the group. Its physiology and processes are only known indirectly, if at all, and it is unknown whether it really possess a dynamic cytoskeleton governing cell shape, vesicle trafficking and primitive organelles (as suggested by [19, 41, 42]).

While there is a strong phylogenetic evidence supporting an archaeal relation of many eukaryotic genes, the archaeal host *per se* is not exactly proven [26]. At most we can say that there is a *substantial* *archaeal contribution* to the eukaryotic genome, either *as* host or *to* host. Assuming that horizontal gene transfer (HGT) was extensive before the nuclear lineage has stabilized, one cannot exclude the possibilities (however unlikely) that archaeal genes were not inherited vertically but acquired horizontally, possibly from other endosymbionts. And there are problems with archaeal host models (e.g. membrane type).

## Symbiont

It is now known that the LECA was mitochondriate and all mitochondria-related organelles (MRO: anaerobic mitochondria, mitosomes, hydrogenosomes) are monophyletic [28, 43, 44] and any loss of mitochondria is secondary and polyphyletic [18, 43, 45-52]. Phylogenetic analyses support overwhelmingly that the ancestor of mitochondria was a member of the Alphaproteobacteria [10, 28, 53-61]. Proto-mitochondria evolved early after the separation of the γ‑ and β‑lineages from the central α‑lineage [62, 63]. Alphaproteobacteria contain photosynthetic, non-photosynthetic, autotrophic, heterotrophic, anaerobic and aerobic species, or even some that can be all of these. There are also entirely endoparasitic clades. The closest extant relative of mitochondria is unknown, but it most probably is in the Rickettsiales. Free-living Alphaproteobacteria (Pelagibacterales, previously the SAR11 clade) have been considered [64, 65] assuming thus an ancestrally free-living mitochondria, though this option was refuted [55, 66-68]. More recent results either suggest methylotrophic Alphaproteobacteria (Rhizobiales) as the closest relatives [62, 69] or Rickettsiales with closest sister clades being Rickettsiaceae and Anaplasmataceae [67]. The latter suggests an ancestor already residing in the host.

Rickettsiales are intracellular obligate parasites of eukaryotes exploiting the phagocytotic machinery to enter cells in food vacuoles and then enter the host’s cytoplasm by lysing the vacuole membrane [70]. Though they enter their host via phagocytosis, there is no evidence that the mitochondrial ancestor also did it with the archaeal host [71]. Extant rickettsias have a much less streamlined genome and their inner-membrane ATP exporters are not of the original eukaryotic origin (found in mitochondria) but probably acquired from plastids [72]. This indicates that their pathogenic evolution has started recently and was a second invasion event, long after the mitochondrial one [12, 72].

1. Hypotheses

## Syntrophy hypothesis

The updated version of the syntrophy hypothesis by López-García and Moreira [1, 73] proposes two serial endosymbiotic events responsible for the origin of nucleus and mitochondria, respectively. Hydrogen transfer mediated syntrophic symbiosis between a strict anaerobic, methanogenic euryarchaeon (the endosymbiotic nucleus), and a fermenting, heterotrophic, hydrogen-producing ancestral gram-negative myxobacterium (a deltaproteobacterial host) lead to the initial association. The archaeal partner ultimately lost its membrane, and invaginating myxobacterial membrane formed the nuclear envelope. The host outer membrane was lost quite early, which improved the exchange of molecules with the external environment. Many metabolic reactions that previously took place in the periplasm could be transferred to other membranous compartments, presumably to mitochondria and/or peroxisomes. Methanogenic metabolism could have been lost up till this point in favour of other anaerobic metabolisms, compatible with the facultatively aerobic mitochondrial ancestor.

The mitochondrial ancestor was engulfed by the myxobacterium early after the archaeal capture, possibly via primitive phagocytosis. It was a highly versatile, facultative aerobe, heterotrophic (primarily non-photosynthetic) alphaproteobacterium, possibly methanotrophic, respiring O_2_ if present, being microaerophilic at least, or other oxidizing molecules, e.g. methane, nitrate or fumarate. During the early phase of the relationship, mitochondria oxidized the archaeal methane and provided CO_2_ in turn and their capacity to utilize oxygen was secondary, or may even have evolved later [73]. The alphaproteobacterium had a methane source, the archaeon had a methane sink, an extra source of CO_2_, and protection against the possible presence of O_2_. The fermenting myxobacterium benefited from the increased methanogenesis rate.

## Photosynthetic symbiont theory

The theory by Cavalier-Smith assumes that the initial benefit of the relationship came from the photosynthetic symbiont [74, 75] (suggested earlier by others [11, 76]). The cenancestral neomuran (the clade consisting of archaea and eukaryotes [77]) performing phagocytosis but lacking a nucleus gave rise to the karyotic proto-eukaryote, that had at least a primitive endomembrane system and cytoskeleton. The host was a gram-positive, facultatively aerobe heterotroph, who probably already had oxidative phosphorylation. It directly answers the origin of bacterial membranes of eukaryotes. It captured a similarly facultatively aerobe photosynthetic purple non‑sulphur bacterium (close to *Rhodospirillum*, see [60]), probably photosynthesizing under anaerobic conditions and respiring under oxic conditions [74, 75, 77]. There were already autogenous oxygen-respiring organelles in the proto-eukaryote (the endoplasmic reticulum and peroxisomes), and most likely these provided preadaptations for the mitochondrial acquisition [75].

At the initial phase, the host was probably preying on the bacterium via phagocytosis. The host was pre-adapted for phagotrophy by secreting digestive exoenzymes [77]. The bacterium leaked some photosynthate (e.g. oxaloacetate) to prevent the host from digesting it. This might have been the case even before engulfment and certainly before any protein importer was inserted into the symbiont’s membrane. After engulfment, the phagosomal membrane failed to fuse with the lysosome and was ultimately degraded, presumably before any protein was inserted into the symbiont membrane, allowing the symbiont to multiply freely in the host’s cytosol. The first step for enslavement need not have to be the insertion of ANT but any carrier would suffice to better extract the photosynthate; and there are many more such carriers. When the endosymbiont was permanently enslaved, the host lost its respiratory machinery.

## Hydrogen-hypothesis

The model of Martin and Müller [10, 78-81], in its updated and extended form, is the most debated mainstream symbiogenetic, mitochondria-first scenario. The host is assumed to be an anaerobic, strictly H_2_ dependent autotroph Euryarchaeon. It doesn’t have to be methanogenic (as it was assumed earlier but then turned out to be untenable), any H_2_-dependent host suffices. The symbiont was a metabolically versatile, facultatively anaerobe but oxygen tolerating ancestral alphaproteobacterium with the capacity of oxidative phosphorylation (respiration) under aerobic conditions and H_2_ production (fermentation) under anaerobic, heterotrophic conditions.

The relationship initially was extracellular metabolic symbiosis driven by hydrogen transfer, and the host’s demand for hydrogen provided both the cause and means for the partnership to work. The alleged advantage of symbiosis apart of metabolic syntrophy could have been tolerance of oxygen in an increasingly oxygen-rich environment, as the alphaproteobacterium could decrease the local oxygen tension for the archaeon. The bacterium itself did not get any metabolic help from the archaeon apart from consuming its waste hydrogen, and thus decreasing local hydrogen tension. The method of inclusion couldn’t have been phagocytosis, as the early host never had the energetic means to operate a sophisticated cell-shaping and membrane-remodelling machinery [82]. It must have been slow engulfment through the syntrophic cell-surface contact. After the merger, the symbiont provided its respiratory chain and H_2_-producing fermentations from the inside, while the host provided fermentable organic compounds. As the host acquired genes for heterotrophic carbon metabolism from the symbiont, there was no longer any selective pressure to maintain its autotrophic lifestyle, resulting in the loss of ETC and bioenergetic membranes [78]. Still, the archaeal ATPase remained under functional constraint and was function reversed to acidify food vacuoles at the expense of cytosolic ATP, presumably those extracted from the mitochondria, as ANTs were inserted in the mitochondrial inner membrane [83]. Lastly, the archaeal host underwent membrane replacement, triggered by vesicles dispatching from the mitochondria [83]. In the long run, the energetic boost provided by the proto-mitochondrion triggered the evolution of phagocytosis, the nucleus and genome expansion [13, 82]. Without the endosymbiont’s energy, no other phagocytotic cell on par with eukaryotes in complexity can be expected.

## Sulfur-cycling hypothesis

Searcy’s scenario postulates metabolic syntrophy based upon reciprocal exchange of sulfur compounds [11, 12, 84-86] (also see [17, 87]). The host was a *Thermoplasma*-like wall-less, sulphur-respiring, facultative aerobic euryarchaeon living in acidic environments (e.g. [12, 17, 85, 87-90]). The first step was that the host lost its cell wall, presumably as an adaptation for contact respiration upon elemental sulfur. A rudimentary cytoskeleton was evolved to increase contact surface of insoluble sulfur. It also made possible to acquire the symbiont via slow engulfment, assuming that the partners already were in a mutual metabolic relationship. The symbiont was an aerobic alphaproteobacterium, closely related to Rhodospirillaceae [84], that oxidized H_2_S to sulfur either photosynthetically or by aerobic respiration; in turn, the host reduced sulfur to H_2_S. Sulfur cycled repeatedly, acting as an electron carrier between the two organisms [12]. The engulfed symbiont provided metabolic compartmentation and higher efficiency and it also separated reactive oxygen producing reactions from the cytoplasm and DNA.

## Origin-by-infection hypothesis

Searcy [12] has also considered that the original bacterial partner was an infectious, Rickettsia-like pathogen living off the host. The host was the same facultative aerobic *Thermoplasma*-like euryarchaeon as above. In this exploitative relationship, the host didn’t benefit at all, while the free-living, alphaproteobacterium (not related directly to modern rickettsias) relied on the organic compounds, possibly ATP, available in the host’s cytosol. The symbiont must have been non-photosynthetic, as otherwise it wouldn’t rely on the host’s organic materials. This unilateral exploitation might render the host crippled, but if not, the relationship could still be unstable as the infected cell had to compete with uninfected ones, leading thus to extinction. However, if the parasite does not reduce the evolutionary fitness of the host too much, there could be stable coexistence and ultimately the pair can evolve to have greater reproductive success.

## Pre-endosymbiont hypothesis

Gray assumes that most of the mitochondrial proteome (80-90%, the non-alphaproteobacterial component, NPC) was already present in the host in the form of an endogenously evolved pre-endosymbiont organelle, the pre-mitochondrion, that preadapted the host for a later acquisition of the alphaproteobacterial symbiont [53, 91] (also see the earlier plasmid sequestration theory by Mahler [92, 93]). The host was an aerobic, phagocytotic pre-eukaryote (perhaps evolving from an archaea) that already had an endomembrane system. The pre-mitochondrion concentrated enzymes and reactants of most present mitochondrial metabolic pathways, like the peroxisome. Thus its benefit was metabolic compartmentation, at the expense of the host’s cytosolic ATP. The organelle already had membrane structural proteins, a protein import mechanism plus ion-, metabolite, aminoacid-, and nucleotide-transporters, however, since it was energy-consuming, its nucleotide carrier system imported ATP and exported ADP unlike how modern mitochondria do it.

Later on an aerobic alphaproteobacterial endosymbiont was acquired by phagocytosis. It gradually took over the pre-mitochondrion’s functions as it performed the same metabolic roles but had its own ATP-generating mechanism and was not relying on cytosolic ATP much. The benefit of the symbiont was to acquire a steady resource of organic compounds from the host’s cytosol. Its metabolic products (except ATP) were exported the same way as products of the pre-mitochondrion, as those transporters could be integrated into the new symbiont easily. Also, the premitochondrion had ANTs, pumping ATP in, which were then inserted into the bacterial symbiont, and having their function reversed to export ATP to the cytosol. This effectively turned the symbiont to an energy generator. Ultimately, the symbiont took over the metabolic role of the pre-mitochondrion, which was reduced, or perhaps retained as the peroxisome [53].

## Phagocytosing archaeon theory

Martijn and Ettema [8] assumes an almost fully eukaryotic, phagocytosing archaea as host (also by [94-96], similar to the original archezoa hypothesis) that acquired mitochondria lastly. The host (most probably from TACK) already had all the eukaryotic inventions (actin, tubulin, the ubiquitin protein modifier machinery, several proteins involved in transcription and translation). The loss of cell wall allowed (but did not trigger selectively) the appearance of flexible actin-based cytoskeleton which could have facilitated the formation of cellular protrusions. It lead to a primitive phagocytotic machinery, capable to capture various prokaryotic cells for digestion. This resulted in massive HGT, accounting for the chimaeric nature of eukaryotes. To protect the genetic material from unwanted recombination the nuclear envelope formed by invagination resulting in a primitive karyotic cell type. At that stage, an alphaproteobacterium was engulfed, establishing an endosymbiotic interaction with the host, leading to a protomitochondrial cell type. It is likely that the symbiotic interaction was already established before the ingestion but there is no explanation on what this early relationship was and why the phagocytosed bacterium was not digested.

## Oxygen detoxification hypothesis

Also known as the ox-tox model. Andersson and Kurland [72, 97-99] (also see [13]) postulates an anaerobic (or less aerobic) but phylogenetically unspecified heterotroph and a free-living aerobic alphaproteobacterium. The host was not specified initially, but it was either an archaeon or a primitive eukaryote [98]. Triggered by the catastrophic atmospheric oxygen increase starting around 2 Ga, the environment became increasingly hostile for the anaerobic host species. This facilitated the metabolic relationship, where the symbiont could remove toxic oxygen locally by respiring (providing a selective edge for the pair) while the host provided the endosymbiont with substrates such as pyruvate, originating from e.g. its own glycolysis [98]. The host slowly engulfed the detoxifying partner, but no phagocytosis is involved.

1. Evaluation of hypotheses along the major questions about observable facts
2. The singular nature of eukaryotes

All eukaryotes primarily have mitochondria or MROs, there are no known second origin of mitochondria nor eukaryotes and there are no sister groups to Eukarya being at par with them regarding complexity. If mitochondria provided such an enormous opportunity as argued by Lane and Martin [100-102], why in 4.5 billion years did eukaryotes only evolve once [103] and no other prokaryote was integrated as a respiring energy provider? Endosymbiosis is widespread within eukaryotes (e.g. plastids [104]), we know that there were transient symbioses contributing to the eukaryotic genome that did not stabilize in the long run [105] and there are also endosymbiotic cases for prokaryotes [106-109]. On the other hand, primary endosymbiosis is extremely rare. There are a handful of options for the why: either there was a barrier for eukaryogenesis (ecological, environmental or energetic) that was only crossed once, or it was a relatively easy step but all other trials failed to survive for some reason. Considering that both prokaryotes and amitochondriate eukaryotes happily exist, it seems unlikely that parallel trials were inferior in fitness terms and went extinct accordingly. While it is possible that new karyotic lineages could not invade already occupied eukaryotic or archezoan niches, we should at least see their phylogenetic signal (which we don’t). Many possible mutual metabolic fusions could have existed before the advent of mitochondria, neither of those turned out to be fruitful. Since such parallel lineages did not appear from time to time, Forterre [5] excludes the latter option.

The only other long-term primary endosymbionts are cyanobacteria engulfed by the ancestor of Archaeplastida [110] (and more recently by *Paulinella* [111-113] and tentatively *Epithium* [114]). Both events happened after mitochondria were acquired (Archaeplastida arose some ~1.6‑1.4 Ga ago [115, 116]). Why is primary endosymbiosis so rare? On the other hand, why is that secondary (and tertiary, etc.) endosymbioses are prevalent in Eukarya? A conservative estimate based on Keeling [117] (also [110, 118]) assumes 10 independent non-primary endosymbiotic events of plastid lineages. Clearly, plastid-endosymbioses happened many times and happened easily, presumably because the phagocytotic machinery and the necessary plastid inner/outer membrane importers (after the first plastid was acquired) were already in place. But why plastids were incorporated multiple times but not mitochondria [104]? If cyanobacteria make so much better partners providing the host with photosynthetic energy, why the very first stable endosymbiont was not a photosynthetic bacterium?

The first option also implicitly requires a uniquely lucky context that made proto-eukaryotes make the leap. Many of the contending scenarios assume multiple rare steps leading to the acquisition of mitochondria: two successive endosymbiotic events in the syntrophy hypothesis [1, 73] (though multiple metabolic symbioses are frequent, see [119]); the evolution of a pre-endosymbiont organelle that preadapted the host for acquiring the real endosymbiont (plus quick reversion of the ANT in the endosymbiont) [53, 91]; or simply the prior invention of phagocytosis [8, 74, 77]. The problem with these scenarios is that no intermediate forms remained. Parasitic symbiont theories [12, 120], by assuming that the parasite reduces the host’s fitness, provide a natural barrier preventing frequent symbiogenesis. Again, there had to be a unique context, for example fluctuating environment, where the host-parasite pair had an advantage over independent individuals.

The traditionally postulated environmental cause for the unique and singular context of eukaryogenesis was the accumulation of free oxygen leading to an oxygen catastrophe, known as the Great Oxygenation Event ([62, 121, 122], ox‑tox hypothesis [98]). Anaerobic species were forced to retreat to anoxic habitats or couple themselves with aerobic partners able to locally reduce toxic oxygen tensions. While the great oxygenation around 2.3-2.4 Ga ago is clearly coupled to oxygenic photosynthesis [123], it remains unproven that oxygenation events were the direct cause of the emergence of eukaryotes. Neither is there a clear evidence that there was a global catastrophe triggered by oxygen increase. Geophysical evidence shows that oxic surface waters throughout the Proterozoic (and during the appearance of the Eukarya) were underlain by anoxic and sulfide-enriched waters [124, 125] meaning that anaerobic habitats were always available widespread and many strictly anaerobic species are thriving there which altogether render the global oxygenation event less likely a discrete catastrophe than a gradual process over perhaps a billion years [123]. Accordingly, it is hard to consider it a unique, singular and sudden environmental event prompting rapid eukaryotic origin.

Lane, on the other hand, argues that the singularity is not a result of an environmental cause or the loss of complex prokaryotes evolved in parallel, which undoubtedly were there [5, 101], but the single acquisition of mitochondria was the trigger. It released an energetic constraint and allowed an increase both in cell and genome size and ultimately lead to the sexual cell cycle and the accumulation of eukaryotic traits in a single population [100-102]. However, the fact that there are (secondarily reduced) amitochondriate eukaryotes in the “archezoan” niche barely having more genes than large prokaryotes (~10K) indicates that for example phagocytosis is feasible without mitochondria. It is very likely that mitochondria-provided energy was indispensable for ultimate genome expansion and further eukaryotic inventions, but Lane’s claim of a 10-fold intermediate genome increase to experiment with new genes early after mitochondrial acquisition is unlikely, for at least two reasons. Firstly, it goes against the concept of gradual evolution of complex traits, as was pointed out by Szathmáry [126], denying the possibility of stepwise accumulation of adaptations. Even a small increase in genome size provides a huge exploration space. Secondly, a ten-fold increase in a reasonable FECA genome would lead to increased rate of replicative errors, making such large proto-eukaryotic genomes untenable without sophisticated error-correcting mechanisms, lacking at that time [127] (the argument is further detailed in S5). Accordingly, it is unlikely, that a huge genome increase was sudden and solely dependent on mitochondrial power [100]. See Figure 2 for a visual understanding of the energetic issue.

Martin speculates that the origin of Haloarchaea (extreme halophile Euryarchaea) may be analogous (though not related) to eukaryogenesis [78] in the sense that an anaerobic archaeon received multiple bacterial genes via HGT most prominently the ETC for oxygen respiring [128, 129]. The major difference in their case is that the bacterial ETC was inserted into the host’s plasma membrane and not into the endosymbiont’s one (as there is no endosymbiont retained, if there were any). While Haloarchaea are not the missing link of eukaryotes, such examples emphasize two things: eukaryotic inventions and processes happened independently in Archaea, and massive HGT is possible without phagocytosis.

The only remaining reasonable theory that accounts for singularity is from Cavalier-Smith who suggests an initially photosynthetic bacterial partner [74, 75]. As the host was a heterotrophic phagocytotic predator, endosymbiosis seems to be inevitable. Furthermore, a photosynthetic prey only has to evolve some importers to exuded photosynthate to control the host. After engulfment, aerobic respiration was energetically more beneficial for the host (after ATP exporters were planted) hence the symbiont didn’t became the first plastid. This could have been the major bottleneck hindering eukaryogenesis, however, it is still not clear why an inevitable endosymbiosis lead to nucleogenesis *only once*. Photosynthetic endosymbiotic purple nonsulfur bacteria are known e.g. in phagotrophic ciliates [130].

1. Lack of intermediates and transitional forms

Either all eukaryotic inventions were rolled out in an extreme short time or there was strong selection in a prolonged time that ensured no intermediates survived. Fast eukaryogenesis seems unlikely, as for example nuclear transfer and gene loss in mitochondria were substantial (e.g. mitochondrial ribosome acquired 19 new proteins [131]) and must have taken a long time (see [98]). There is a large group of eukaryote-specific, well-conserved genes and complex subcellular structures that suggest a rather long evolutionary trajectory instead of an accelerated one after the merger [132]. Even for plastids (where the host might have had some advantage as it had already gained access to endosymbiotic membrane proteins once [133]) the transition is thought to have happened over a long period [104].

On the other hand, if there was strong selection for new inventions and against intermediates, what could this selective force be? A similar transition was the emergence of the genetic code which also lacks intermediates, primarily disparate codings. However, in case of the code, there *was* a channeling force: the benefit of consensus. If all organisms speak the same code they can exchange information horizontally without having to switch between dictionaries. Clearly, the optimum is to have one shared dictionary instead of multiple. The analogy however is not flawless: it is hard to see how interference of intermediate proto-eukaryotes could single out a particular LECA. Were all the intermediates inferior fitness-wise? This seems unlikely in light of living amitochondriate eukaryotes.

The third (and most likely) option is to accept that some prokaryotes are truly descendants of early intermediates. There are many features thought to be exclusive to Eukarya that were found in prokaryotes, e.g. cytoskeletal components like crenactin in Archaea [134] and tubulin homologs in Bacteria [135] and Archaea [136], membrane remodeling ability [137], histones in Crenarchaeota [138, 139], even endosymbiosis [106, 107] (for more, see [100]). The problem is that independent evidences do not form a gradual evolutionary route to their fully fledged eukaryotic form, but rather indicates independent origins. Furthermore, while there are rudimentary archaeal cytoskeletons, there are no phagocytotic Archaea, nor primarily amitochondriate eukaryotes with nucleus or organelles, nor mitochondriate prokaryotes. A negative result (lack of something) however is not a proof: there might be extant intermediates or at least clues we haven’t found yet. Gray suggests that the remnant of the pre-endosymbiont organelle might be the peroxisome [53]. However, it is still not clear whether peroxisomes (as pre-endosymbionts) predate mitochondria or the other way around [140, 141].

As a matter of fact, only one theory postulates a strong-enough selective force that could account for the elimination for intermediate stages: the parasitic symbiont hypothesis [12], where transient stages are assumed to be ecologically unstable. However, Assuming that other inventions evolved in parallel with mitochondria, this provides an explanation for the LECA event horizon [142].

1. The chimaeric nature of eukaryotes and membrane conversion

The host wither originated as an archaeon or as a bacterium. In case of an archaeal host, one has to explain the origin of both bacterial membranes and bacterial metabolic enzymes. In case of a bacterial host, archaeal information machinery needs an explanation.

Considering an archaeon, the hydrogen hypothesis assumes that it was the endosymbiont that provided the bacterial genes for glycolysis, and heterotrophy for the host [10, 78] (similarly in the sulfur-cycling hypothesis), as hydrogen-dependency excludes heterotrophy [143]. Müller et al. found that genes of glycolysis have a strong similarity with proteobacterial homologs, leading to the assumption that glycolytic enzymes (except enolase) were not inherited from the archaeal host, but from the endosymbiont [43]. However, their data only indicates a strong association with proteobacteria, but not explicitly with the alpha lineage (also see [98]). While many mitochondrial genes survived in the nucleus, none of these are of the core heterotrophic metabolism. All recent phylogenies of the pyruvate metabolizing enzymes associated with anaerobes show eukaryotes as monophyletic, however, the closest related prokaryotic homologs to eukaryotes are never alpha-, rather delta- or epsilonproteobacterial or from firmicutes [105]. Even if modern eukaryotic glycolysis is of alphaproteobacterial origin, according to Martin [10, 78], that does not mean that the host never had an equivalent process, that was inferior and replaced by the symbiont’s. The ox-tox and photosynthetic symbiont hypotheses bypass the problem by assuming an already heterotrophic host. Similarly, in the phagocytosing archaeon theory, the host is primarily heterotrophic, and the phagocytosis-induced extensive HGT accounts for the mosaic nature of the genome and the faster evolution of the nuclear genes [8]. This explains the larger evolutionary distance between eukaryotic and archaeal genes in comparison with the stronger similarity between Alphaproteobacteria and mitochondria. Since phagocytosis predates mitochondria, it also explains the origin of the non-alphaproteobacterial components (NPC), as these were acquired due to HGT before the endosymbiont was captured.

If the host was a bacterium, either a gram-negative (from e.g. Deltaproteobacteria [73] or the Planctomycetes-Verrucomicrobia-Chlamydiae clade [5]) or a gram-positive (a neomuran) [74, 75], the membrane is primarily bacterial and conversion doesn’t have to be accounted for. Presumably, the host then received archaeal genes *en* *masse* [5, 7] and not via independent HGT from various sources as the archaeal components of eukaryotes associate closely with TACK archaea and not with diverse groups. Unless another, archaeal endosymbiont is assumed (e.g. [1], though not supported phylogenomically and ecologically), it is hard to account for the tight phylogenetic match of archaeal genes in eukaryotes. Not surprisingly, the scenario that a bacterial host selectively replaced its whole genetic machinery (including ribosomes) with archaeal counterparts via multiple HGT from different sources has never been proposed, as it seems even more problematic than membrane replacement.

Non-alphaproteobacterial genes present in eukaryotes can be also explained by either the host or the mitochondrial ancestor acquiring them horizontally *before* the merger [144]. Cavalier-Smith claims archaeal components to originate vertically from the ancestral neomuran that gave rise both to eukaryotes and to archaea. López-García and Moreira [1] (and [5]) postulates a second, archaeal endosymbiont that formed the pre-nucleus before mitochondria. While the syntrophic theory readily explains the chimaeric nature of the eukaryotes (archaeal genes from the endosymbiotic archaea, bacterial membranes from the myxobacterium, anaerobic enzymes of modern mitochondria from the facultatively aerobe alphaproteobacterial ancestor), it does not answer why the archaeal genome took over the consortium and not the myxobacterial one. If it was by chance, then we should see alternative eukaryotes with (myxo)bacterial informational genes. Plus the myxobacterial host (being gram-negative) had to lose its outer membrane and dismantle the archaeal endosymbiont’s membrane just to set up instead the nuclear envelope by invagination, postulating thus two more steps. Ultimately, phylogenomic analyses do not support a third, major genome donor apart of the archaeal and proteobacterial sources [145], discrediting thus endosymbiotic nucleus models.

If the host was an archaeon, the bacterial-type eukaryotic membranes (cytoplasmic, endoplasmic, nuclear) need an explanation. Bacterial lipid synthesis was either acquired via HGT possibly before the symbiont, or the endosymbiont itself triggered the conversion and provided the bacterial lipids [71, 83]. Consequently, there had to be a prolonged time during which stable gene-, and lipid-exchange between partners could establish the new membrane organization (see the scenario of Dey [38]). Furthermore, if any replacement of the host’s plasma membrane has ever happened then it only seems logical that the endomembrane system and the nuclear envelope (being invaginations of the plasma membrane) had evolved *after* the modifications done to the membrane.

Martin speculates that vesicles detaching from mitochondria converted the host’s membranes from archaeal to bacterial [83]. Mitochondrial derived vesicles are formed from the outer membrane of intact mitochondria [146] that fuse whit the peroxisomes and lysosomes for degradation. While membrane replacement is not necessary for eukaryogenesis at Cavalier-Smith, his neomuran origin of Archaea does postulate it (though in reverse direction) when they branched off. (Note, that the neomuran hypothesis postulates a simple replacement from a non-archaeal ancestor, regardless of mitochondria, while Martin believes that the host was a bona fide archaeon that already had mitochondria when the replacement happened.) Either way, no such transition between archaeal and bacterial membranes (in any direction) is known [147]. There are other factors against the neomuran hypothesis: there are known archaea with double membranes (*Ignicoccus hospitalis* [148, 149]), which weakens the assumed conservative nature of the number of cell membranes and therefore it cannot be used to support the monophyly of archaea, eukaryotes and gram-positive bacteria [147].

One can assume that membrane replacement, if ever happened, was gradual, as any sudden transition was likely to break down metabolism. A slow transition on the other hand would involve mixed membranes – something that is unknown in nature. Mixed membranes were assumed to be unstable ([150], also when pre-cells formed [151, 152]), though an experimental study indicates that mixed liposomes of archaeal and bacterial lipids are stable [153]. Probably the replacement of complex, natural membranes is not easy, as any change in lipids most require a change in the specifically adapted integral proteins depending on the lipid context [154]. The fact that bacterial importers acquired are retained actively in archaeal membranes (in Haloarchaea [128]) seems to indicate, that mixing one type of membrane with proteins of another type is not a problem.

Willams [155] pointed out that the genetic background for the synthesis of both types of lipids (archaeal and bacterial) are common to all eukaryotes, archaea and bacteria (e.g. the archaeal isoprene synthesizing mevalonate pathway is ancestral to all three domains, originating from the last universal common ancestor [156]), suggesting that neither the transition from ester to ether lipids in the common ancestor of all Archaea, nor a subsequent reversion in Eukarya, would require radical genomic changes (cf. [147, 157]). A clear experimental support of this would be to genetically engineer an archaeon to replace its own G1P isoprenoid lipids with bacterial G3P lipids, or at least have a single cell where both biosynthetic pathways are active and produce mixed membranes.

There is one important question about motives. A conversion from archaeal to bacterial membrane in eukaryotes seems unnecessary, as non-extremophile archaea living in normal conditions still retain their archaeal membrane, indicating that it does not pose any disadvantage. Therefore, theories postulating an archaeal host have to account for a selective advantage of using the bacterial membrane over the archaeal one, and not replacing the symbiont’s membrane with archaeal lipids (at least it was smaller). **At present there is no clear selective advantage of any membrane over the other**. Lastly, it must be emphasized that even if replacement happened during eukaryogenesis there is no known other case in life’s history where membranes were replaced, not even locally for an organelle.

1. Lack of membrane bioenergetics in host

All membranous compartments in prokaryotes are involved in energy metabolism via chemiosmotic coupling [1]. No eukaryotes have electron transport chains (ETC) in their plasma membranes. Clearly, the host lost all components of membrane bioenergetics and ATP synthesis other than the A‑ATPase itself (continuing as V-ATPase with its function reversed to acidify lysosomes). If the host was autotrophic, and initially performed photosynthesis and/or respiration via its bioenergized plasma membrane, how and when did its membrane loose its ETC and phosphorylation ability? Was it the consequence of integrating the endosymbiont (as Martin assumes [78]) or the host primarily lacked ETC in its plasma membrane?

The latter would mean that the host either had other, internal bioenergetic membranes overtaking energy metabolism from the plasma membrane prior to mitochondria, or that the host (primarily) relied solely on fermentation and entirely lacked an ETC, which is unlikely (though there are such strictly fermentative bacteria, e.g. lactic acid bacteria [158]). There are various solutions proposed for endogenously derived features to relieve the plasma membrane of its bioenergetic functions, like the endomembrane system postulated by [75, 159] or [8]). These organelles provided selective advantage by both increasing the bioenergetic surface and localizing reactions in a compartment to increase efficiency. Either endogenous or exogenous of origin, the organelle that implemented this metabolic compartmentation ultimately rendered the host’s energy metabolism obsolete. The major difference between endomembranes and mitochondria is that the first could have immediately provided the selective advantage while the latter had to be fully enslaved before paying off.

One important consequence of relinquishing energetic functions in the membrane is that it can then be used for other purposes, e.g. for full-time phagocytosis. Losing membrane bioenergetics before mitochondria usually also means that the host was already heterotrophic and phagocytosis came before mitochondria – as an autotrophic host would not rely on phagotrophy. Endogenous bioenergetic membranes could provide a moderate energetic boost *without* mitochondria – something that might still fall short to support phagocytosis, according to [82, 100, 102]. Nevertheless, evolving intracytoplasmic membranes is a quantitative change, doesn’t require specific qualitative changes in metabolism or regulation. It could have evolved gradually and might have supported a primitive phagocytotic machinery – you don’t have to be super-efficient, when there is no defenses evolved yet by your prey. Especially if all necessary components (binding the prey, excreting digestive enzymes and uptaking food) were already evolved before membrane remodelling [160].

1. Non-photosynthetic mitochondria

Both Alphaproteobacteria and Cyanobacteria are able to both photosynthesize and generate ATP through an ETC. Why was the former lost in mitochondria (see [161])? Was the photosynthetic capacity primarily missing in the mitochondrial ancestor (i.e. it was a free-living bacterium that already lost photosynthesis) or it was lost when the bacterium was engulfed? Did early photosynthesis in the latter case play any part in symbiogenesis? If the mitochondrial ancestor was photosynthetic, why did it not become the first plastid, especially in the light that later on, *only* photosynthetic endosymbionts were incorporated as plastids?

Most theories never considered that the symbiont was photosynthetic. The single scenario that not just postulates but requires a photosynthetic symbiont discussed here is by Cavalier-Smith [74, 75, 162] (originating from Woese [76]; also see [11, 126, 130, 163]). According to this theory (complying with the study of Esser [60]) the ancestral mitochondrion was a photosynthetic purple non-sulphur bacterium. The loss of photosynthesis happened during the streamlining of the mitochondrial genome, when the host focused on gaining more energy out of the slave. Purple nonsulfur bacteria can photosynthesize in light and respire in the dark, but not both at the same time. Since respiration provided more energy, photosynthesis was slowly reduced [74]. Furthermore, loss of photosynthesis occurred repeatedly within proteobacteria and among eukaryote algae [74]. Also, as the host was phagotrophic, it could supply the endosymbiont with a carbon source both in aerobic and anaerobic conditions. Since during the latter, photosynthesis does not work, mutations can easily eliminate photosynthetic ability under unfavorable anoxic conditions.

However, since the study of Esser, most recent analyses exclude photosynthetic species (Rhodobacterales [66, 69] and Rhodospirillales [63, 67, 69]) and the obligate endosymbiotic parasites of the Rickettsiales from the direct ancestry of mitochondria [69]. Rather, the ancestral symbiont was associated with extant alphaproteobacterial methylotrophs [62, 69] or was assumed to be already residing in the host as a possible parasite [67], meaning that they primarily lack the photosynthetic capability.

1. Origin of anaerobic MROs

It is generally assumed that the ancestral mitochondrion already had oxidative phosphorylation and an aerobic energy metabolism equivalent to that of today’s mitochondria [48, 61, 75, 99, 164] and thus anaerobic mitochondria, mitosomes and hydrogenosomes are secondary, independent adaptations to anaerobic environments [49, 98, 105, 165]. Accordingly, the ancestral mitochondrion was either aerobic or facultatively aerobic at least; the retained capacity to grow microaerobically or anaerobically might account for the modern distribution of secondarily anaerobic MROs [75].

The hydrogen and syntrophy hypotheses argue for the fact that anaerobic metabolism in modern MROs is ancestral and anaerobic pyruvate enzymes (e.g. PFO, PNO, PFL) derive from the facultatively aerobe proto-mitochondrion’s mixed metabolism. Hydrogen producing enzymes either originate from the ancestral mitochondrion [10] or from the myxobacterial host [1]. However, there is no direct evidence supporting an alphaproteobacterial origin of enzymes responsible for anaerobic energy metabolism in eukaryotes [105] and the signal for the myxobacterial contribution is weak [1]. While phylogenies of the pyruvate metabolizing enzymes associated with anaerobes show eukaryotes as monophyletic [166], the closest bacterial homologs are never from the Alphaproteobacteria [105]. These could be masked by extensive HGT [43], but interestingly enough, enzymes of aerobic metabolism (either in the nucleus or in the mitochondrial DNA) are not masked by the same HGT and kept their close similarity with alphaproteobacterial homologs [105]. Available data indicate that non-standard mitochondrial gene functions present in MROs are not the primary descendants of the corresponding gene functions in the Alphaproteobacteria but are secondary adaptations evolved from pre-existing mitochondrial and host nuclear genes [99, 165]. Stairs et al. compellingly argue for the independent origins of anaerobic MROs in various eukaryotic groups, as a result of adaptation to anaerobic environments via gene loss and/or HGT (either by the host digesting prey or by transient endosymbionts) most probably after phagocytosis was fully fledged and major eukaryotic clades have diversified [105].

1. Evaluation of hypotheses along the major questions about historicals
2. Metabolism of host

Though phylogenetically the host seems to be closest to Archaea, its original metabolism, anaerobic or aerobic, photosynthetic or heterotrophic, is still debated. The nature of the host is important for multiple reasons. Its metabolism specifies whether the proposed symbiont was compatible or not while its trophic nature specifies the mechanism of inclusion. Energy-wise, it is certainly gram-positive fermentative bacteria lacking effective membrane bioenergetics or lacking membrane ETC overall (i.e. no respiration, no photosynthesis) that could benefit the most in relative terms from an energy-producing endosymbiont. It would also explain the lack of bioenergetics in eukaryotic membranes and the bacterial-type single membrane of eukaryotes. However, unlike crime scenes, one cannot simply assume the perpetrator by finding the bacteria who would have benefited the most.

If the host was a crenarchaeote (eocyte) or from another TACK group [16, 18, 20, 167-169], a methanogenic metabolism is unlikely [145] as methanogenetic species mostly belong to Euryarchaeota ([158], cf. [71]). Methanogenesis doesn’t really fit the picture for metabolic reasons either: its strict anaerobic nature makes it incompatible with aerobic respiration and mitochondria; there is no trace of methanogenesis in the eukaryotic genome; extant methanogenic archaeal genes do not resemble archaea-related eukaryotic genes (though mitochondrial genes in eukaryotes do resemble their alphaproteobacterial homologs); there is no plausible evolutionary path leading from anaerobic methanogenesis to aerobic metabolism [12, 77]; there are clues that the host’s ancestral metabolism was already (at least facultatively) aerobic or microaerophilic [17, 74, 87, 170]. The same arguments apply to the syntrophy hypothesis of [1], where the methanogenetic partner endosymbiotically contributed to the nucleus. In general, there are so many arguments pointing against a methanogenic host, that Martin has abandoned it in favor of a general anaerobic (but oxygen-tolerating) metabolism, producing H_2_ [10, 78]. There is another problem though with hydrogen-based metabolism. While Rhodospirillales can produce H_2_ fermentatively, mitochondria group robustly with Rickettsiales instead [67, 171] - neither of which is capable of hydrogen production.

The lack of methanogenetic genes in eukaryotes can be primary or secondary, just like the lack of photosynthetic genes in mitochondria. Both methanogenesis and photosynthesis were lost multiple times independently in respective prokaryotic groups, which again points to the extreme versatility and adaptability of prokaryotes to their environments. A note is in order: methanogenic syntrophy, while refuted by many, is nevertheless a viable option – it is only incompatible with fully aerobic environments (and metabolism). If the ancestral symbiont was facultatively aerobic and methanotrophic [1], it could have been able to perform metabolic exchange with the methanogenic partner in case of anaerobic conditions. Later on, as the environment was becoming more oxygenated, aerobic respiration became the prominent pathway of the bacterial partner, rendering methanogenesis unnecessary. Since aerobic respiration is more efficient energetically, it was a highly beneficial selective step and also a one-way route as methanogenic genes were subsequently lost.That most of the eukaryotic energy metabolism is assumed to be of proteobacterial origin (glycolysis, TCA cycle, etc. [43, 105]) does not mean, that the host didn’t have archaeal or bacterial, possibly metabolically inferior equivalents. As a matter of fact, some studies didn’t find significant proof for the bacterial origin of glycolytic enzymes [172] or alphaproteobacterial origin for anaerobic enzymes of energy metabolism in eukaryotes [105] (and references thereof), contrary to what [10] assumed. According to the syntrophy [1] and the neomuran [7, 74, 173] hypotheses the host already had bacterial metabolic enzymes, either vertically inherited (those that do not associate with proteobacteria in phylogenetic analyses), or acquired via HGT (possibly from proteobacteria).

As there is a growing amount of evidence that the ancestral symbiont was aerobic, or at least facultatively aerobic, cf. [53, 174, 175], it only seems reasonable to assume the host to be aerobic, or at least tolerating mild oxygen exposure. Cavalier-Smith postulated that the host already had oxidative phosphorylation [75, 77]. After taming the symbiont, the LECA probably had mixed anaerobic and aerobic metabolism [43], and a dynamic cytoskeleton that used up lots of energy.

If the host was already phagotrophic before the symbiont, it had to be heterotrophic and might have relinquished most of its bioenergetic membranes (see above). Ultimately, the question will be decided by closing in on the host phylogenetically – or perhaps finding clues in eukaryotes of an ancient, archaeal oxidative phosphorylation process. If the host was at least facultatively aerobic than strictly anaerobic metabolic syntrophies are not viable. If the host already had the capacity for oxidative phosphorylation, the benefit provided by the symbiont couldn’t be as large as in case of an anaerobically respiring host. Lokiarchaea, proposed closest living relative of the host, were claimed to be hydrogen dependent based on genome reconstruction, that lends some support to the hydrogen [176]and syntrophy hypotheses, with the caveat that no living lokiarchaeon has yet been observed.

1. Metabolism of symbiont

While the endosymbiont is clearly of alphaproteobacterial origin, its metabolic nature is similarly debated as the host’s (cf. [67, 69, 75]). Was it photosynthetic or heterotrophic, could it tolerate oxygen, did it prefer oxic or anoxic environments, was it a free-living prey, an ectosymbiotic partner or was already exploiting the host? The early metabolic role accounts for the initial benefit of the endosymbiont and possibly for the method of inclusion. There is a wide range of metabolism done by extant mitochondria related organelles and neither ATP synthesis, nor respiration is common to all [28].

Traditional views postulate a strict aerobic ancestor of mitochondria [98], while most symbiogenetic models postulate a facultative aerobe, suggesting that eukaryotes first evolved in anoxic or microaerophilic environments [1, 10, 73]. Unfortunately, pinpointing the ancestral metabolism of modern mitochondria is far from trivial. It turned out that no single, ancestrally unifying metabolic feature of modern mitochondria exists that is shared by all MROs (not even iron-sulphur cluster assembly [177-179]) [47]. This is understandable if one compares various mitochondrial genome sizes. While one can speculate about the original function of ancestral mitochondria based on gene and protein phylogenies, it is a fact that the largest mitochondrial genome consists of 2-~70 genes [180] and the standard genome size of the intracellular parasitic Alphaproteobacteria (e.g. *Bartonella* or *Rickettsia*) is around 834-1600 genes [98]. This means that the genome was reduced by at least one order of magnitude, and most protein coding genes were lost in the reductive process.

It now seems adequately supported that the ancestral symbiont was at least facultatively aerobic (cf. [53, 164, 181]), capable of oxidative phosphorylation under low oxygen condition [175], and all anaerobic mitochondria are secondarily descended by polyphyletic gene losses [48, 49]. Degli Esposti and co., by reconstructing the ancestral metabolism claim that complementary molecular and genetic analyses of bioenergetic proteins indicate that the pathway stemming from methylotrophic Alphaproteobacteria (Rhizobiales) is the most probable route of mitochondrial evolution though they only looked at proteins involved in bioenergy production both in bacteria and mitochondria [69]. Also, their second best candidate is *Rhodopseudomonas*, which is photosynthetic (i.e. it’s less parsimonious as it also had to lose its photosynthetic capability). Gray, reviewing recent proteomic studies [53], conclude that the ancestral mitochondrion was probably a (facultatively) aerobic APB, with an ETC and the capability for oxidative phosphorylation, TCA cycle and pyruvate metabolism, with an essentially complete protein import apparatus [53, 182], which was surely required for a stable host-endosymbiont relationship before any nuclear gene-exchange could have happened. Also prominent are metabolite transporters in the reconstructed mitochondrion in these studies, which might hint that the initial metabolic relationship was the exchange of small metabolites (photosynthates) between host and symbiont, as suggested by [74]. Wang and Wu postulate, that the pre-mitochondrion had an ATP/ADP translocase (unrelated to the one installed later by the host) that the symbiont used to extract energy from the host’s cytosol, rendering it thus a parasite instead of an energy provider [67].

Searcy’s sulfur-cycling hypothesis was based on the early result that mitochondria derived from Rhodospirillaceae [60], called purple sulfur bacteria (*sic*) by Searcy [12, 84]. However, according to most recent phylogenomics, purple sulfur bacteria belong to the Chromatiales (Gammaproteobacteria) [158], Rhodospirillaceae are actually purple nonsulfur bacteria, and neither the *γ*‑lineage, nor Rhodospirillaceae are the closest relatives of mitochondria. This does not exclude however that the ancient mitochondrial ancestor was a sulphur-oxidizing alphaproteobacterium.

One clue that could inform about the ancestral metabolism of early mitochondria is its internal membrane system. Endomembranes are quite common in prokaryotes, evolved multiple times independently for respiration or photosynthesis, as invaginations of the plasma membrane (e.g. in Cyanobacteria, Proteobacteria and specifically in Alphaproteobacteria [137, 183, 184] and methanotrophic bacteria [185]). A recent study found prokaryotic homologs of proteins involved in the morphogenesis of mitochondrial cristae (MICOS complex; lacking in cristae-less anaerobic mitochondria, e.g. in hydrogenosomes), moreover, they found that homologs of the core Mic60 are present only in other Alphaproteobacteria (though not exactly in Rickettsiales), thus being ancestral to the mitochondrion [186]. It is possible, though unlikely, that the Mic60 was recruited later for membrane invaginations and was evolved for some entirely different function. The real question though is that if cristae predate engulfment, did they evolve to increase metabolic surfaces for respiration or for photosynthesis?

1. Initial relationship

Before any evolutionary adaptation is fixed, it must have been preceded by an ecologically stable period. Often the partnership is assumed to be initially mutual, though, benefits and costs are not mutually exclusive [12], which suggests that there could have been severe costs for individual partners. The initial relationship couldn’t have been ATP harvesting by the host in exchange for carbon compounds (cf. [58, 98, 187]), as the ATP/ADP translocase (ANT), apparently of eukaryotic origin [72, 187], was added later [74]. Furthermore, a free-living bacterium wouldn’t want to pump ATP to the environment nor could it find free ATP, so the ancestor very likely lacked a nucleotide translocase in its plasma membrane. This fact, along with others (endosymbiosis was slow; loss of host’s ETC in all membranes; initial lack of protein import mechanism, etc.) indicates that present metabolic roles cannot be assumed for the ancestral partners. The relationship and ecological setup of host and symbiont must have been different to what they are now and their initial relationship might not have been mutual at all.

Most theories postulate metabolic syntrophy that preexisted the engulfment of the symbiotic partner. Cavalier-Smith assumes that the host used up oxygen and excreted CO_2_ while the symbiont leaked photosynthate by fixing CO_2_ (and breathed oxygen, but since they were both facultative aerobes, this was of no importance) [74]. The sulphur-cycling hypothesis builds on the facts that sulfur requires little activation energy, well suited for biological redox reactions, is widely used in both anoxic and oxic environments and was widely available on Earth, even before O2 [12]. While H_2_S is highly toxic, inhibiting mitochondrial respiration, it is not harmful if kept below 1 *µ*M [12], which could explain the dire need of metabolic cooperation of host and symbiont. The hydrogen hypothesis assumes that the symbiont required reduced, fermentable organic compounds from the host, in exchange for producing hydrogen. However, the host, being autotrophic, couldn’t provide these in excess, neither had it katabolic pathways to generate such compounds from e.g. lysing a few (ecto)symbionts (not that it had any such enzymes, as it was not heterotrophic). Hence, there is an unaccounted, unstable phase until the host acquired the symbiont’s genes for carbon metabolism and importers and ultimately developed a heterotrophic lifestyle [78], which, if wasn’t extremely short, was probably detrimental. However, there are too many eukaryotic-specific genes that evolved during this time to support an extremely rapid merger. At least this alleged speed is not visible from Alphaproteobacteria-, and Archaea-related eukaryotic genes.

Apart of the metabolic compatibility of host and symbiont, there are ecological considerations as well. Theories that require the aerobic partner of the syntrophic consortium to reduce oxygen-tensions [13, 98] fail to explain why the symbiont later took residence inside the host, and not dispersed over its surface, to prevent the diffusion of oxygen [12]. Other syntrophic theories take it granted that if there is metabolic compatibility, there will be syntrophy. However, in many cases, compatibility is not enough. Even if there is a real demand on the sink side and there is excess at the source side, there could be other restricting factors (see the problem with the ox-tox theory below).

Phagocytotic scenarios usually assume that the host preyed on the mitochondrial ancestor, but since they cannot answer how unilateral predation turned to mutual cooperation (why the host didn’t digest away its prey), they postulate a pre-existing metabolic symbiosis [8, 53, 74, 91]. There are some theories that explicitly suggest a parasitic bacterial ancestor [12, 120]. If the relationship was not mutually beneficial initially, there had to have an advantage for the pair as a unit.

1. Early selective advantage

Before the ATP-exporting mechanism was in place, the relationship could have been costly to one of the parties. There had to be an overall benefit at the level of the pair balancing the costs so that the newly forming unit of evolution had the selective edge over individual competitors. Why and how was a possibly complicated and costly early relationship evolutionary stable and advantageous? How could both partners benefit such that selfish cheaters (those not conforming to the rules) could not disrupt the cooperation?

The selective advantage could have been in effect even before enclosing the symbiont, and in this case, it refers to the benefit that the partnership received so that it could outcompete their free-living relatives. The late benefit is clear: metabolic support from the host is returned as the ATP generated by the symbiont. The metabolic compartmentation (assumed by all theories) and ATP resource, of course, increased bioenergy production enormously [100]. Through it couldn’t be used to explain the early stability and selective superiority of the relationship before ANT was inserted.

There are multiple other factors proposed as selective advantage for the initial pair. Oxidative phosphorylation was suggested early [188, 189] and more recently [101, 190] as an immediate benefit as it can provide a huge energetic boost, but only if the host didn’t already had it, in which case it has no direct benefit. More recent syntrophic (and other) theories assume a facultatively aerobic host [12, 17, 74, 87], which effectively removes the advantage of (re)gaining oxidative phosphorylation. On the other hand, protecting the cytoplasm and DNA from oxygen or reactive oxygen species was also a candidate factor [12, 13, 97]), though now it is generally rejected. The topology doesn’t make sense (why are oxygen-detoxifying symbionts in the inside if oxygen first had to diffuse through the cytoplasm [12]?), environmental causes are debatable (anoxic environments, e.g. below-surface oceans, were not saturated at all by the oxygen increase), protection against oxygen is now done by completely different agents in modern eukaryotes (for more, see [101]). The fundamental flaw in the ox-tox theory (and similar) however is that it assumes that the external oxygen is sinked by mitochondria to reduce oxidation damage due to reactive oxygen species (ROS) – while in reality, it is the mitochondria (and plastids) that produce most of the ROS.

Postulating a *facultatively* aerobic symbiont for an anaerobic host (like the syntrophy hypothesis), does not solve the problem: either there was oxygen, in which case the symbiont is no use protecting against it, or there was no oxygen at all, which then makes it unnecessary to postulate oxidative phosphorylation for the mitochondria. Similarly, the early benefit of mitochondria couldn’t have been oxidative phosphorylation if the host already had it (see aerobic host scenarios, especially [74]).

ATP generation as the core benefit made its comeback more recently in the pre-endosymbiont theory [91]. Mitochondria could overtake the role of the endogenous pre-endosymbiotic organelle (responsible for aerobic respiration) by being able to generate ATP themselves, assuming that they had the same metabolic role. In this case, ATP is indirectly useful, as the symbiont does not provide ATP, though neither does it feed on the host’s pool. However, among other problems (like the lack of phagocytotic archaea and membrane conversion), this theory is unable to specify what the original relationship was between host and symbiont, before engulfment. Furthermore, it expects that the ANTs (coming from the pre-endosymbiont, where they pumped ATP in) were immediately reversed in function when they were inserted into the real symbiont’s membrane, otherwise the symbiont would have consumed all the host’s cytosolic ATP. If ANT was not inserted into the symbiont membrane at all, than what triggered its reversal?

Cavalier‑Smith offers one such case where phagocytosis could have been prevented by the symbiont [74] and where oxidative phosphorylation was not the benefit provided by the symbiont, both partners being aerobic. A photosynthetic symbiont could have leaked photosynthate to prevent direct digestion by a heterotrophic host – and this provided more relative benefit to a photosynthetic than to a heterotrophic symbiont (assuming both being aerobe). This could work even before engulfment, and could have provided the selective edge for the host to survive situations where there was no available carbon source for a heterotroph. This idea was picked up by [191], who proposed that it was resource-poor conditions that lead to the mutual association between partners to provide a selective advantage over non-associated species. Consequently, an originally predatory relationship was turned to prudent harvesting so that in poor times the host had a population of living bacteria inside its cytoplasm to prey on when needed [126]. The prey, being farmed, could also survive hard times. When the host finally evolved ANT, the relationship turned to unilateral exploitation. This, however, still leaves some questions unanswered. Why would a photosynthetic bacterium suffer from the same ecological hardships as a heterotrophic archaeon does, so that it worth for it to be farmed?

Blackstone proposes an interesting scenario based on the selective digestion premise under stressful conditions, putting emphasis on ecological and evolutionary considerations instead of on simple metabolic compatibility [103]. The proto-mitochondria could in turn emit ATP to stabilize the host and avoid its own (and possibly its copies’) digestion. However, selfish symbionts able to switch off their ANT will have a selective advantage on the short run. Without ANT, such a mutant would convert all of its ADP to ATP and due to high metabolic performance, reactive oxygen formation would ultimately damage it. ANT, in turn, would provide selective advantage to those symbionts that have it, as it maintains a state 3 metabolism, where ADP/ATP ratio is high, but ROS are only produced in moderate amounts, and possibly the host would digest those mitochondria being damaged due to lack of ANT.

In the origin-by-infection scenario, the initial infection must have dealt a reproductive disadvantage for the host, rendering the pair selectively inferior [12]. Searcy did not provide any resolution to this issue, neither did he explain why the pathogen was temperated to symbiosis instead of the host evolving defensive measures to get rid of the infection (though he points out that such reversals did happen many times with parasites). It is certainly possible that initially defective parasites induce resistance and ultimately dependence in the host [192] where adaptations are also required from the parasite to invade and survive within the host [193].

1. Mechanism of inclusion

According to the endosymbiotic origin, the alphaproteobacterial endosymbiont entered the host’s cytoplasm in some way. If it was engulfed by the host, there should be (at least) three membranes wrapping the mitochondrion – though there are only two. A host-derived membrane has either degraded or never existed at all. The latter would mean that the invading bacterium was a parasite that could penetrate the host’s membrane, without bursting the cell. If the host was phagocytotic, who or what provided the energy requirements of the expensive machinery before actually having mitochondria? Why the prey was not digested? The three main mechanisms are therefore phagocytosis, slow (syntrophic) inclusion and bacterial invasion (see Table 1).

Unfortunately, there is no evidence of a third membrane ever wrapping mitochondria, which could decide between engulfment or invasion. The outer mitochondrial membrane is of alphaproteobacterial origin, supported by the clear sequence similarity of outer membrane proteins between mitochondria and Alphaproteobacteria [74, 77, 194-197]. Phagocytotic [74] and syntrophic scenarios [1, 10] generally assume that the third membrane was simply degraded spontaneously or by the host (or lysed by the endosymbiont as in the inside-out hypothesis [71]), however, no plausible model of this process was ever proposed. The lack of a third membrane indicates a non-phagocytotic entry for those suggesting parasitic origins.

While theoretically possible, it is unlikely, that the modern outer mitochondrial membrane was derived of the host (either from a bacterium or from an archaeon after membrane conversion) and the alphaproteobacterial outer membrane was lost instead. As the periplasmic space of bacteria (even in gram-positive ones with a single membrane) is an essential metabolic compartment of energy harnessing, and is kept as such in mitochondria, this is unlikely. The lack of a host-derived membrane however does not mean that there wasn’t one ever. Primary cyanobacterial endosymbiosis also ended up with two membranes, but in that case it is more obvious that there was a phagosomal membrane around the plastid that was lost, as the LECA is thought to have been phagocytotic [26, 198], plus non-primary plastids still retain the phagosomal wrapper [104, 110] (which does not hinder their replication or metabolic function at all, though require more sophisticated import signaling and mechanism [199]). However, this was questioned by Koonin and Yutin on the same grounds as for the mitochondrion: the lack of a phagosomal membrane around the first plastids makes it questionable whether the ancestral bikont was really phagocytotic [94]. On the other hand, while there are known parasitic Alphaproteobacteria, no parasitic Cyanobacteria are known, and the ancestry of phagocytosis (at least within LECA) seems rather sound (see [95, 200]).

There is a growing amount of evidence that prokaryotes, and especially TACK archaea have eukaryotic-homolog cytoskeletal proteins [38, 134, 136, 201, 202] and nucleotide-driven dynamic filament systems and membrane remodelling capabilities [134, 137, 203, 204] (also in Asgard [40] and Loki archaea [20], though pangenome data also indicates the lack of endocytosis [205]). Contrary to the claims of Lane and Martin [100], it looks like operating a dynamic filament system in prokaryotes is energetically far from impossible, and simple phagocytosis could exist even without a dynamic actin cytoskeleton [200] and it could have been enough to engulf the mitochondrial symbiont (and many more), ultimately leading to the multiple independent evolution of complex phagocytosis in eukaryotes [94, 206]. Furthermore, a limited capability could be more than enough to induce massive HGT which could have been responsible for rapid evolution by selectively recruiting new genes captured from transient symbionts (or lysed food) [8, 206].

Phagocytotic host theories [7, 8, 19, 20, 91, 94, 121, 142, 150, 206-208] however are generally silent on how the prey eluded digestion. According to Martijn and Ettema [8] and Cavalier-Smith [74], being eaten was avoided possibly due to a pre-existing symbiotic interaction between the two parties. But it is not explained how the host could selectively prevent lysing food vacuoles after it took bacteria in. There is one more questions prompted by the phagocytotic origin: was the phagosomal membrane degraded before or after host protein insertions? It seems easier to explain insertions if there is one less membrane, however, losing a membrane requires extra time, which further prolonged a possibly unstable relationship lacking proper exchange mechanisms.

The facts that there exist cases of endosymbiosis among prokaryotes that do not rely on phagocytosis [106-109] and that all archaea have a cell wall (except Thermoplasma) and consequently, are incapable of phagocytosis [77] led some to entirely drop an early phagocytosis scenario [209]. Syntrophic scenarios [1, 10, 12] assume that the symbiont was slowly engulfed by extending cytoplasmic protrusions around them until they are enclosed. For energetic reasons, Lane and Martin find it the only possible way to kick start eukaryogenesis [100]. Others however find it problematic to accept that a prokaryote lacking a dynamic cytoskeleton engulfed another cell [160]. However, just like phagocytosing prokaryotes, no syntrophic engulfment is documented or known. Most syntrophic scenarios do not explain why a syntrophic consortium that functions fine leads to bacteria being internalized. Furthermore, there is no clear model to explain the vertical inheritance of half-engulfed stages. On the other hand, phagocytosis-induced endosymbioses are abundant and stable (e.g. non-primary plastids).

The third option is that the symbiont itself enters a non-phagocytotic host, possibly as a predator or parasite [12, 41, 120] (also mentioned by [72, 99]). It seems to be supported by the widespread parasitism in Rickettsiales and the lack of a phagosomal membrane. While modern intracellular parasites evolved much later and independently of mitochondria to specialize to eukaryotic hosts [67, 205], it was found that the ANT in plastids and parasitic rickettsias are homologs, probably originating in the alphaproteobacterial ancestor of mitochondria and Rickettsia (different from modern mitochondrial ANT that originates from the host) [72, 175]. It suggests that the ancestral symbiont was equipped with a nucleotide transporter that could steal ATP from the host [72, 99, 175].

In case of a parasitic symbiont there is no need to assume expensive phagocytosis for the prokaryotic host (though it helps) or sophisticated anti-digestion measures for the symbiont, as it resides in the cytoplasm directly. However, it requires some inventions from the bacterial side to enter the host without bursting it (see [132]). Searcy didn’t provide a mechanistic model of parasitic inclusion [12]. Bacterial intracellular predators are known, e.g. *Bdellovibrio* and *Daptobacter*, that both penetrate their hosts [210]. The problem with puncturing the membrane, as *Bdellovibrio* does it, is that it usually causes the death of the host. Note, that present endoparasitic Rickettsia (and Holosporales) enter the host via its own phagocytotic mechanism and not by puncturing its plasma membrane, though *Holospora*, when inside, can cross the host’s internal membranes. Bacteria like *Burkholderia*, invade *Rhizopus* using chitinolytic enzymes, without affecting host integrity [211]. However, there is no contemporary examples of a bacterial endosymbiont resident in an archaeal host, neither there are parasitic Cyanobacteria, which explains (in the parasitic scenario) why photosynthesizers had to wait until a proper phagocytotic mechanism was in place [41].

One interesting recent model suggests that prokaryotic predators first invaded the periplasmic space of a double membraned archaeal host and were captured in the invaginations of the plasma membrane [120]. Consequently, the plasma membrane became the endomembrane system of the host (nuclear envelope included) and the outer membrane became the single plasma membrane. This idea seemingly accounts for the chimaeric nature, the single membrane of eukaryotes, and oxygen-detoxifying species on the outside – however, the proposed membrane changes seem problematic. It does not account for the continuous topology of the double nuclear membrane around the nucleoplasm, neither does it explain how all the inner-membrane proteins were repositioned in the outer membrane, and how the host’s cytoplasmic machinery got into the periplasm, that became the new cytoplasm. Nevertheless, it provides a fresh deviation from cemented ideas.

1. Vertical transmission

At any point in eukaryogenesis, the forming new unit of evolution, a chimaeric cell, must divide so that its selective advantage is heritable. What ensured vertical transmission of the endosymbiont (cf. [212]). Before nuclear transfer, mitochondrial genes were all transmitted via the vertical inheritance of the proto-mitochondria. Unless there is no vertically inherited endosymbiont, partnership can easily be disrupted by selfish parasitic/pathogenic traits in horizontal spread (see [213]). If there is a mechanism that can ensure vertical transmission (e.g. the inside-out theory [71]), then there is reasonable selective drive for cooperation to evolve and beneficial mutations to fixate. The fact that nuclear transfer of genes is not an instantaneous process and perhaps there wasn’t even a nucleus at that point, one has to explain how the partnership remained stable without the evolutionary means to control any possible selfish mutation.

In nucleus-before-mitochondria theories (e.g. [1, 13, 132, 173, 214]), the cell nucleus or at least a separated nuclear compartment predates the symbiont. Such a protection might have helped the genetic transfer, the nuclear lineage being more stable than if genes mingle in the cytoplasm. However, the ecological relationship of host and symbiont must have been stable on a much faster timescale, where one cannot expect to have evolutionary processes to stabilize.

In phagocytotic and syntrophic theories, the phagosomal membrane had to be degraded for the symbiont to be able to freely multiply in the cytoplasm. However, present bacterial symbionts (and plastids) retaining the phagosomal membrane prove that their reproduction is not hindered by the extra membrane. Furthermore, in syntrophic scenarios, there had to be large-scale surface contact between host (cytoplasm) and proto-mitochondria for a long-enough time to increase the probability of vertical inheritance of the symbiont, but also excluding parasites (see [71]). On the other hand, if the symbiont was a parasite, once inside, it could naturally reproduce in the host’s cytoplasm and are transmitted vertically when it undergoes fission.

1. The energetic argument of eukaryotic origins

Apart from heavy criticism [215] (also see its rebuttal [216]), there is a fundamental flaw in Lane’s argument (adopted by Martin [100-102]), recognized by Szathmáry [126]. Lane argues that mitochondria provided the necessary energy surplus for eukaryotic complexification and for an ultimate 200 000-fold genome increase. This might be true in general (though for criticism, see [212]), but he also claims that there had to be an *intermediate increase* in gene count for eukaryotic traits (like phagocytosis) to be evolved *de novo*, as the cell had to be able to experiment with new gene families, and this cost was payed for by mitochondria. Consequently, the fact that “archezoan” eukaryotes evolved reductively *and* the claim that eukaryotic inventions were costly lead to his false implicit conclusion that amitochondriate eukaryotes can only be evolved reductively, but not directly from prokaryotes, and hence no primarily amitochondriate eukaryotes are expected [101] (see Figure 2).

Even Lane admits he does not know the factor of the intermediate increase in gene counts, but guesses at a 10-fold increase ([101] p. 22). This suggested intermediate increase is problematic on two accounts. Firstly, there is no objection against any single eukaryotic trait (phagocytosis included) to evolve gradually, without experimenting first with costly new gene families [126]. The only arguments might be that 1) we don’t see them (yes we do, e.g. dynamic cytoskeleton [94, 134, 135]) and 2) they are too expensive to evolve (yes, but so was photosynthesis and it did evolve in cyanobacteria). “Archezoa” are indeed secondarily reduced but this does not mean that no prokaryote can evolve to their level primarily, without mitochondria.

Secondly, any sudden multiplication of the gene count would result in untenable amount of replicative errors. A recent modelling study concluded that regularly spaced replication origins could alleviate this problem. However, as the replicon size (or genome size before multiple origins) increases above 250 Mb, the probability of double-fork stall errors goes above 1%. As the replicon size further increases, errors become inevitable and rather sophisticated postreplicative mechanism are required which were supposedly not present in early eukaryotes [127]. Accordingly, it is unlikely, that a huge genome increase was sudden and solely dependent on mitochondrial power [100] before multiple replication origins or error detection and repair were evolved.

Once the host has integrated mitochondria, it is certainly easy to just multiply them to cheaply gain additional energy. But keep in mind that the taming of mitochondria did not happen immediately, meaning that energy was not available for multiplication at all *ab initio*. Before mitochondrial modularity, the simplest possible way to gain some energy was probably to increase internal respiratory membrane surfaces, which does not require more genes as cyanobacteria testify. Of course, it did not provide 1000-fold energy increase, perhaps only 10-fold, but this could have been enough for a few new genes and adaptations to accumulate and phagocytosis to emerge.

# Supplemental figures


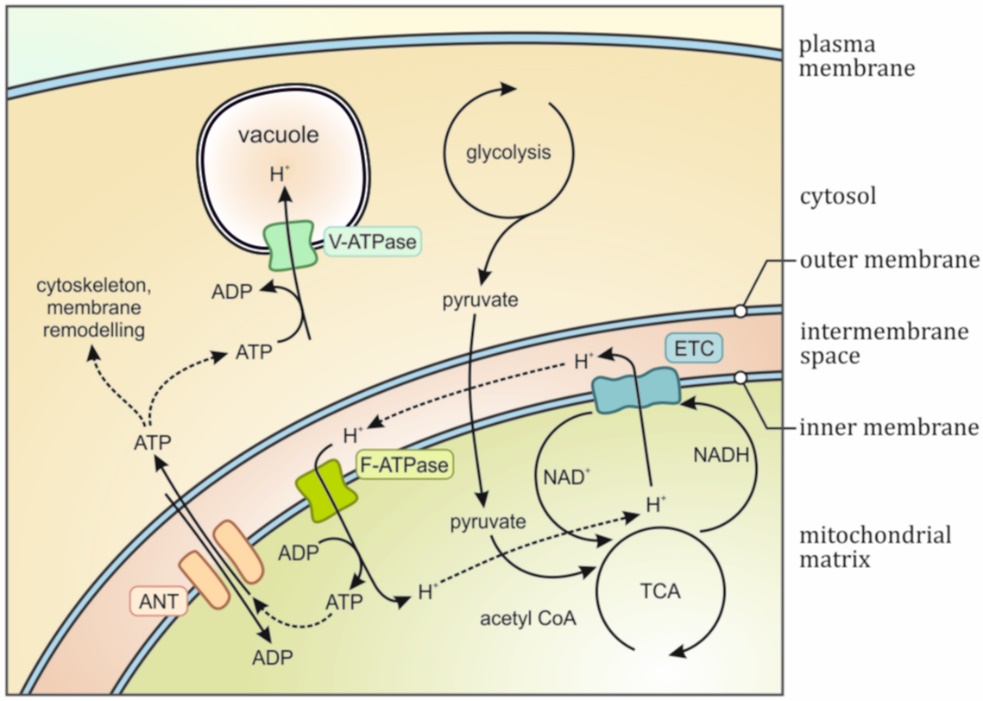


Figure S1. Simplified eukaryotic cell organization, focusing on modern energy production of mitochondria. The mitochondrion (at the bottom) has a double membrane. In its inner membrane are the electron transport chain (ETC) letting protons into the intermembrane space (originally the periplasmic space) during respiration. The F‑ATPase harnesses the proton gradient to produce ATP, which, in turn, is pumped out by the ADP‑ATP translocase into the cytosol where it is used up in various ways (operating V-ATPase to acidify vacuoles, cytoskeleton, etc.). Cristae are omitted for sake of simplicity.

# Supplemental tables

Table S1. Possible combinations of components and scenarios representing various combinations. Hypothesis in bold are discussed and evaluated in detail in the main text. AN = anaerobe, A = aerobe (at least facultatively), U = unspecified). Most of the theories assume a facultatively aerobic alphaproteobacterial symbiont.

|  |  | host (cytoplasm and possible nucleus) | | |  |  |
| --- | --- | --- | --- | --- | --- | --- |
|  |  | **primitive eukaryote** | **archaeon** | **bacterium** |  |  |
| ecological relationship (inclusion mechanism) | **syntrophy (+ / +) (engulfment)** | - Archezoa hypothesis [194, 217] | - inside-out theory [71, 218] |  | **U** | **host metabolism when acquiring mitochondria** |
|  |  |  | - viral eukaryogenesis (+virus as nucleus) [219] - **hydrogen hypothesis** [10, 78-81] - organic acid syntrophy [85, 188, 189] - oxygen detoxification theory of Vellai et al. [13] | - **syntrophy hypothesis** (+archaeon as nucleus) [1, 73] | **AN** |  |
|  |  | - **ox-tox model** [97, 98] | |  |  |  |
|  |  |  | - **sulfur-cycling hypothesis** [11, 12, 84-86] |  | **A** |  |
|  | **predation (+ / -) (phagocytosis)** |  | - **phagocytosing archaeon theory** [8] | - PTV hypothesis [5] | **U** |  |
|  |  |  | - viral eukaryogenesis [219] |  | **AN** |  |
|  |  | - **pre-endosymbiont hypothesis** [53, 91] |  | - **photosynthetic symbiont theory** [74, 75] - peroxisomal system before mitochondria (+archaeal LGT) [122, 150] | **A** |  |
|  | **parasitism or bacterial predation (- / +) (invasion)** |  | - **origin-by-infection hypothesis** [12] |  | **U** |  |
|  |  |  | - bacterial predation [120] |  | **AN** |  |
|  |  |  |  |  | **A** |  |

Table S2. Answers of the various mitochondrial origin hypotheses to questions about observed facts. HGT = horizontal gene transfer, MRO = mitochondria-related organelles.

| **Hypothesis** | **Eukaryotic singularity** | **Absent intermediates** | **Chimaeric eukaryotes and membrane conversion** | **Lack of membrane bioenergetics in host** | **Non-photosynthetic mitochondria** | **Origin of anaerobic MROs** |
| --- | --- | --- | --- | --- | --- | --- |
| **Hydrogen hypothesis** [10, 78-81] | mitochondrial energy surplus relaxed the energetic barrier and made genome increase and new inventions possible; Haloarchaea as second origin? | only mitochondrial cells could reach higher complexity, transition was short, intermediates quickly disappeared (missing mitochondriate but primarily non-phagocytotic cells, mixed membranes, etc.) | archaeal genes are vertically inherited; mitochondria-produced vesicles fused with archaeal plasma membrane, leading to gradient replacement | archaeal A-type ATPase, that was re-targeted and function-reversed to the would-be-lysosome, a vesicle that originated from the outer membrane of the mitochondria. | symbiont was primarily non-photosynthetic | primarily derived anaerobic MROs: ancestral proto-mitochondrion was already a facultative aerobe, that could selectively loose/retain genes and functionality in various clades |
| **Photosynthetic symbiont theory** [74, 75] | endosymbiosis was probably a necessity after phagocytosis | ? (missing remnants of photosynthesis-genes in modern mitochondria, primarily amitochondriate eukaryotes, phagocytotic bacteria) | extensive HGT accounts for archaeal genes, non-alphaproteobacterial genes were directly inherited from the bacterial ancestor; plasma membrane is primarily bacterial type, archaeal membrane conversion? | host was already a phagocytotic heterotroph that relinquished plasma membrane respiration prior to mitochondria | secondarily lost in symbiont (host benefited more from a respiring than from a photosynthesizing symbiont) | ancestral proto-mitochondrion was photosynthesizing under anaerobic conditions and respiring under oxic conditions; anaerobic mitochondria are secondarily derived |
| **Syntrophy hypothesis** [1, 73] | ? | ? (missing genes of methanogenic metabolism in the eukaryotic genome, amitochondriate or anucleated proto-eukaryotes showing myxobacterial features, remnants of the ancient archaeal membrane around the DNA) | archaeal genes are vertically inherited from the nuclear endosymbiont; plasma membrane is primarily bacterial type | original myxobacterial outer membrane is lost when the mitochondria and peroxisome appear (as the periplasm is inferior in energy metabolism). The archaeal membrane dissolves when the methanogenic capacity Is not needed anymore (due to mitochondria) | symbiont was primarily non-photosynthetic | anaerobic enzymes from ancestral mitochondria, PFO from myxobacterium |
| **Phagocytosing archaeon theory** [8] | the mitochondrial energetic boost was required to make the step | ? (missing phagocytosing archaea) | archaeal genes are vertically inherited; phagocytosis-induced HGT; membrane conversion? | ? | ? (symbiont was probably primarily non-photosynthetic) | ? |
| **Pre-endosymbiont hypothesis** [53, 91] | ? (internal organelles are rare in prokaryotes) | ? (missing remnants of the pre-endosymbiont organelle (peroxisome?), primarily amitochondriate eukaryotes, phagocytosing archaea, archaea with a metabolic organelle) | mitochondrial NPC originate from pre-endosymbiotic HGT in host; membrane conversion? Does not account for the origin of archaeal genes | pre-mitochondria took over the bioenergetic functions from the plasma membrane; the pre-endosymbiont organelle did not respire | ? (symbiont was probably primarily non-photosynthetic) | ? |
| **Sulfur-cycling hypothesis** [11, 12, 84-86] | ? | ? | archaeal genes are vertically inherited; membrane conversion? | ? | ? | does not account for different mitochondrial types |
| **Origin-by-infection hypothesis** [12] | ? | either the parasite is tamed and then all intermediates are naturally inferior, or the parasite ultimately caused the extinction of the lineage | archaeal genes are vertically inherited; membrane conversion? | ? | ? (symbiont was probably primarily non-photosynthetic) | ? |
| **Oxygen detoxification** **hypothesis** [97-99] | great oxygen catastrophe | ? | ? | ? | ? | anaerobic MROs are secondarily derived |

Table S3. Answers of the various mitochondrial origin hypotheses to questions about evolutionary unknowns. The ecological relationship of host and symbiont is indicated with H/S, where + and - stands for beneficial and disadvantageous relationship, respectively. Since metabolic compartmentation by the (proto-)mitochondrion is assumed by all theories after engulfment and integration therefore it is ignored, focusing on the advantage that predated engulfment and lead to metabolic efficiency.

| **Hypothesis** | **Metabolism of host** | **Metabolism of symbiont** | **Initial relationship** | **Early selective advantage** | **Mechanism of inclusion** | **Vertical transmission** |
| --- | --- | --- | --- | --- | --- | --- |
| **Hydrogen hypothesis** [10, 78-81] | anaerobic, H_2_ dependent, strict autotrophic archaeon | highly versatile, facultative anaerobe able to respire in aerobic, or ferment (and produce H_2_) under anaerobic conditions | +/+; anaerobic metabolic syntrophy driven by H_2_ transfer | metabolic syntrophy, H_2_-sinking capacity of symbiont | selection for an increasing surface area lead to slow syntrophic engulfment | ? |
| **Photosynthetic symbiont theory** [74, 75] | phagocytotic, facultatively aerobe gram-positive bacterium | photosynthetic, facultatively aerobe alphaproteobacterium | +/-; preying, later metabolic syntrophy, driven by exchange of photosynthate from symbiont and CO_2_+minerals from host | photosynthesis, leaked photosynthate for host | phagocytosis; the phagosomal membrane failed to fuse with lysosomes and thus subsequently was degraded and lost | ? (free replication only after the phagosomal membrane was lost) |
| **Syntrophy hypothesis** [1, 73] | fermenting, heterotrophic, H_2_-producing ancestral gram-negative myxobacterium (and strict anaerobic methanogenic euryarchaeon) | highly versatile, facultative aerobe (non-photosynthetic) alphaproteobacterium | +/+; metabolic syntrophy, driven by methanotrophy | initially, the nuclear symbiont provided CH_4_ for the alphaproteobacterial symbiont, that provided CO_2_ in turn; later on, the mitochondria protected against O_2_ | some form of simple phagocytosis both for the nucleus and mitochondria (syntrophic surrounding of alphaproteobacteria by the consortium in [73]) | ? |
| **Phagocytosing archaeon theory** [8] | phagocytotic heterotrophic TACK archaeon | aerobic (?) alphaproteobacterium | +/-; unspecified symbiotic relationship; preying | ? (energy production for host, even before engulfment) | phagocytosis | ? |
| **Pre-endosymbiont hypothesis** [53, 91] | aerobic, phagocytotic heterotrophic pre-eukaryote | aerobic alphaproteobacterium | +/+; steady resource of organic compounds for the symbiont; host doesn’t have to provide ATP for the symbiont | symbiont runs on its own ATP instead of host’s; why and how was ANT reversed? | phagocytosis | ? |
| **Sulfur-cycling hypothesis** [11, 12, 84-86] | wall-less, sulphur-respiring, (facultatively) aerobic euryarchaeon (not compatible with modern phylogenomic results) | aerobic alphaproteobacterium, oxidizing H_2_S photosynthetically or by aerobic respiration | +/+; metabolic symbiosis mediated by reciprocal sulfur-transfer | ? (separating ROS-producing reactions) | increasing contact surface on elemental S lead to inclusion of partner | ? |
| **Origin-by-infection hypothesis** [12] | (same as above) | aerobic free living rickettsia-like alphaproteobacterium turned intracellular parasite | -/+; parasitic alphaproteobacterium, consuming host's organic nutrients, possibly metabolites, even ATP | none | ? | parasites can naturally multiply inside the host |
| **Oxygen detoxification** **hypothesis** [97-99] | anaerobic (or less aerobic) unspecified heterotroph, either an archaeon or a primitive eukaryote [98] | free-living aerobic alphaproteobacterium | +/+; metabolic syntrophy driven by oxygen tolerant and oxygen sensitive species | symbiont decreases local oxygen tension | ? (assumingly non-phagocytotic) | ? |

# Supplemental references

[1] López-García P, Moreira D (2006) Selective forces for the origin of the eukaryotic nucleus. BioEssays 28: 525–533.

[2] Lake JA, Rivera MC (1994) Was the nucleus the first endosymbiont? Proceedings of the National Academy of Sciences of the United States of America 91: 2880.

[3] Horiike T, Hamada K, Miyata D, Shinozawa T (2004) The origin of eukaryotes is suggested as the symbiosis of *Pyrococcus* into γ-proteobacteria by phylogenetic tree based on gene content. Journal of Molecular Evolution 59: 606–619.

[4] Gupta RS, Golding GB (1996) The origin of the eukaryotic cell. Trends in Biochemical Sciences 21: 166–171.

[5] Forterre P (2011) A new fusion hypothesis for the origin of Eukarya: better than previous ones, but probably also wrong. Research in Microbiology 162: 77–91.

[6] Cavalier-Smith T (1987) The origin of eukaryote and archaebacterial cells. Annals of the New York Academy of Sciences 503: 17–54.

[7] Cavalier-Smith T (2014) The neomuran revolution and phagotrophic origin of eukaryotes and cilia in the light of intracellular coevolution and a revised Tree of Life. Cold Spring Harbor Perspectives in Biology 6: 1–31.

[8] Martijn J, Ettema TJG (2013) From archaeon to eukaryote: the evolutionary dark ages of the eukaryotic cell. Biochemical Society Transactions 41: 451–457.

[9] Koonin EV, Yutin N (2014) The dispersed archaeal eukaryome and the complex archaeal ancestor of eukaryotes. Cold Spring Harbor Perspectives in Biology 6.

[10] Martin W, Müller M (1998) The hydrogen hypothesis for the first eukaryote. Nature 392: 37–41.

[11] Searcy DG (1992) Origins of mitochondria and chloroplasts from sulfur-based symbioses, Singapore: World Scientific. pp. 47–78.

[12] Searcy DG (2003) Metabolic integration during the evolutionary origin of mitochondria. Cell Research 13: 229–238.

[13] Vellai T, Takács K, Vida G (1998) A new aspect to the origin and evolution of eukaryotes. Journal of Molecular Evolution 46: 499–507.

[14] Rivera MC, Jain R, Moore JE, Lake JA (1998) Genomic evidence for two functionally distinct gene classes. Proceedings of the National Academy of Sciences of the United States of America 95: 6239–6244.

[15] Zillig W (1991) Comparative biochemistry of Archaea and Bacteria. Current Opinion in Genetics & Development 1: 544–551.

[16] Lake JA, Henderson E, Oakes M, Clark MW (1984) Eocytes: a new ribosome structure indicates a kingdom with a close relationship to eukaryotes. Proceedings of the National Academy of Sciences of the United States of America 81: 3786–3790.

[17] Margulis L, Dolan MF, Guerrero R (2000) The chimeric eukaryote: Origin of the nucleus from the karyomastigont in amitochondriate protists. Proceedings of the National Academy of Sciences of the United States of America 97: 6954–6959.

[18] McInerney JO, O’Connell MJ, Pisani D (2014) The hybrid nature of the Eukaryota and a consilient view of life on Earth. Nature Reviews Microbiology 12: 449–455.

[19] Pittis AA, Gabaldón T (2016) Late acquisition of mitochondria by a host with chimaeric prokaryotic ancestry. Nature 531: 101–104.

[20] Spang A, Saw JH, Jorgensen SL, Zaremba-Niedzwiedzka K, Martijn J, et al. (2015) Complex archaea that bridge the gap between prokaryotes and eukaryotes. Nature 521: 173–179.

[21] Williams TA, Embley TM (2014) Archaeal “dark matter” and the origin of eukaryotes. Genome Biology and Evolution 6: 474–481.

[22] Guy L, Saw JH, Ettema TJ (2014) The archaeal legacy of eukaryotes: a phylogenomic perspective. Cold Spring Harbor Perspectives in Biology 6: a016022.

[23] Raymann K, Brochier-Armanet C, Gribaldo S (2015) The two-domain tree of life is linked to a new root for the Archaea. Proceedings of the National Academy of Sciences 112: 6670-6675.

[24] Lasek-Nesselquist E, Gogarten JP (2013) The effects of model choice and mitigating bias on the ribosomal tree of life. Molecular Phylogenetics and Evolution 69: 17–38.

[25] Saw JH, Spang A, Zaremba-Niedzwiedzka K, Juzokaite L, Dodsworth JA, et al. (2015) Exploring microbial dark matter to resolve the deep archaeal ancestry of eukaryotes. Philosophical Transactions of the Royal Society of London B: Biological Sciences 370: 20140328.

[26] López-García P, Moreira D (2015) Open questions on the origin of Eukaryotes. Trends in Ecology & Evolution 30: 697–708.

[27] Koonin EV (2010) The origin and early evolution of eukaryotes in the light of phylogenomics. Genome Biology 11: 209.

[28] Embley TM, Martin W (2006) Eukaryotic evolution, changes and challenges. Nature 440: 623–630.

[29] Poole AM, Penny D (2007) Evaluating hypotheses for the origin of eukaryotes. BioEssays 29: 74–84.

[30] Gribaldo S, Poole AM, Daubin V, Forterre P, Brochier-Armanet C (2010) The origin of eukaryotes and their relationship with the Archaea: are we at a phylogenomic impasse? Nature Reviews Microbiology 8: 743–752.

[31] Guy L, Ettema TJG (2011) The archaeal ’TACK’ superphylum and the origin of eukaryotes. Trends in Microbiology 19: 580–587.

[32] Forterre P (2013) The common ancestor of Archaea and Eukarya was not an archaeon. Archaea : 1–18.

[33] Lake JA (1988) Origin of the eukaryotic nucleus determined by rate-invariant analysis of rRNA sequences. Nature 331: 184–186.

[34] Woese CR, Fox GE (1977) Phylogenetic structure of the prokaryotic domain: The primary kingdoms. Proceedings of the National Academy of Sciences of the United States of America 74: 5088–5090.

[35] Harris JK, Kelley ST, Spiegelman GB, Pace NR (2003) The genetic core of the universal ancestor. Genome Research 13: 407–412.

[36] Ciccarelli FD, Doerks T, von Mering C, Creevey CJ, Snel B, et al. (2006) Toward automatic reconstruction of a highly resolved tree of life. Science 311: 1283–1287.

[37] Yutin N, Makarova KS, Mekhedov SL, Wolf YI, Koonin EV (2008) The deep archaeal roots of eukaryotes. Molecular Biology and Evolution 25: 1619–1630.

[38] Dey G, Thattai M, Baum B (2016) On the archaeal origins of eukaryotes and the challenges of inferring phenotype from genotype. Trends in Cell Biology 26: 476–485.

[39] Hartman H, Fedorov A (2002) The origin of the eukaryotic cell: A genomic investigation. Proceedings of the National Academy of Sciences 99: 1420-1425.

[40] Zaremba-Niedzwiedzka K, Caceres EF, Saw JH, Bäckström D, Juzokaite L, et al. (2017) Asgard archaea illuminate the origin of eukaryotic cellular complexity. Nature 541: 353–358.

[41] Ball SG, Bhattacharya D, Weber APM (2016) Pathogen to powerhouse. Science 351: 659–660.

[42] Archibald J (2015) Endosymbiosis and eukaryotic cell evolution. Current Biology 25: R911–R921.

[43] Müller M, Mentel M, van Hellemond JJ, Henze K, Woehle C, et al. (2012) Biochemistry and evolution of anaerobic energy metabolism in eukaryotes. Microbiology and Molecular Biology Reviews 76: 444–495.

[44] Hrdý I, Hirt RP, Dolezal P, Bardonova L, Foster PG, et al. (2004) *Trichomonas* hydrogenosomes contain the NADH dehydrogenase module of mitochondrial complex I. Nature 432: 618–622.

[45] Ku C, Nelson-Sathi S, Roettger M, Sousa FL, Lockhart PJ, et al. (2015) Endosymbiotic origin and differential loss of eukaryotic genes. Nature 524: 427–432.

[46] Koonin EV (2015) Archaeal ancestors of eukaryotes: not so elusive any more. BMC Biology 13: 84.

[47] van der Giezen M (2009) Hydrogenosomes and mitosomes: conservation and evolution of functions. Journal of Eukaryotic Microbiology 56: 221–231.

[48] Embley TM, van der Giezen M, Horner DS, Dyal PL, Foster P (2003) Mitochondria and hydrogenosomes are two forms of the same fundamental organelle. Philosophical Transactions of the Royal Society of London B: Biological Sciences 358: 191–203.

[49] van der Giezen M, Tovar J, Clark CG (2005) Mitochondrion-derived organelles in protists and fungi. In: A Survey of Cell Biology, Academic Press, volume 244 of *International Review of Cytology*. pp. 175–225. doi: [10.1016/S0074-7696(05)44005-X](http://dx.doi.org/10.1016/S0074-7696(05)44005-X). http://­www.sciencedirect.com/­science/­article/­pii/­S007476960544005X.

[50] Tovar J, León-Avila G, Sanchez LB, Sutak R, Tachezy J, et al. (2003) Mitochondrial remnant organelles of *Giardia* function in iron-sulphur protein maturation. Nature 426: 172–176.

[51] Tovar J, Fischer A, Clark CG (1999) The mitosome, a novel organelle related to mitochondria in the amitochondrial parasite *Entamoeba histolytica*. Molecular Microbiology 32: 1013–1021.

[52] Bui ET, Bradley PJ, Johnson PJ (1996) A common evolutionary origin for mitochondria and hydrogenosomes. Proceedings of the National Academy of Sciences 93: 9651–9656.

[53] Gray MW (2015) Mosaic nature of the mitochondrial proteome: Implications for the origin and evolution of mitochondria. Proceedings of the National Academy of Sciences 112: 10133-10138.

[54] Lang BF, Burger G (2012) Mitochondrial and eukaryotic origins: a critical review, Amsterdam, the Netherlands: Academic Press, Elsevier, volume 63 of *Advances in Botanical Research*. pp. 1–20.

[55] Rodríguez-Ezpeleta N, Embley TM (2012) The SAR11 group of alpha-proteobacteria is not related to the origin of mitochondria. PLoS ONE 7: e30520.

[56] Gray MW, Doolittle WF (1982) Has the endosymbiont hypothesis been proven? Microbiological Reviews 46: 1–42.

[57] Gray MW (1992) The endosymbiont hypothesis revisited. International Review of Cytology 141.

[58] Andersson SGE, Zomorodipour A, Andersson JO, Sicheritz-Pontén T, Alsmark UCM, et al. (1998) The genome sequence of *Rickettsia prowazekii* and the origin of mitochondria. Nature 396: 133–140.

[59] Lang BF, Gray MW, Burger G (1999) Mitochondrial genome evolution and the origin of eukaryotes. Annual Review of Genetics 33: 351–397.

[60] Esser C, Ahmadinejad N, Wiegand C, Rotte C, Sebastiani F, et al. (2004) A genome phylogeny for mitochondria among α-proteobacteria and a predominantly eubacterial ancestry of yeast nuclear genes. Molecular Biology and Evolution 21: 1643–1660.

[61] Gray MW (2012) Mitochondrial evolution. Cold Spring Harbor Perspectives in Biology 4.

[62] Degli Esposti M (2014) Bioenergetic evolution in proteobacteria and mitochondria. Genome Biology and Evolution 6: 3238–3251.

[63] Williams KP, Sobral BW, Dickerman AW (2007) A robust species tree for the Alphaproteobacteria. Journal of Bacteriology 189: 4578–4586.

[64] Thrash JC, Boyd A, Huggett MJ, Grote J, Carini P, et al. (2011) Phylogenomic evidence for a common ancestor of mitochondria and the SAR11 clade. Scientific Reports 1.

[65] Georgiades K, Madoui MA, Le P, Robert C, Raoult D (2011) Phylogenomic analysis of *Odyssella thessalonicensis* fortifies the common origin of *Rickettsiales*, *Pelagibacter ubique* and *Reclimonas americana* mitochondrion. PLoS ONE 6: e24857.

[66] Brindefalk B, Ettema TJG, Viklund J, Thollesson M, Andersson SGE (2011) A phylometagenomic exploration of oceanic alphaproteobacteria reveals mitochondrial relatives unrelated to the SAR11 clade. PLoS ONE 6: e24457.

[67] Wang Z, Wu M (2015) An integrated phylogenomic approach toward pinpointing the origin of mitochondria. Scientific Reports 5.

[68] Viklund J, Ettema TJ, Andersson SGE (2012) Independent genome reduction and phylogenetic reclassification of the oceanic SAR11 clade. Molecular Biology and Evolution 29: 599–615.

[69] Degli Esposti M, Chouaia B, Comandatore F, Crotti E, Sassera D, et al. (2014) Evolution of mitochondria reconstructed from the energy metabolism of living bacteria. PLoS ONE 9: e96566.

[70] Emelyanov VV (2001) Rickettsiaceae, *Rickettsia*-like endosymbionts, and the origin of mitochondria. Bioscience Reports 21: 1–17.

[71] Baum DA, Baum B (2014) An inside-out origin for the eukaryotic cell. BMC Biology 12: 76.

[72] Amiri H, Karlberg O, Andersson GES (2003) Deep origin of plastid/parasite ATP/ADP translocases. Journal of Molecular Evolution 56: 137–150.

[73] Moreira D, López-García P (1998) Symbiosis between methanogenic archaea and δ-proteobacteria as the origin of eukaryotes: The syntrophic hypothesis. Journal of Molecular Evolution 47: 517–530.

[74] Cavalier-Smith T (2006) Origin of mitochondria by intracellular enslavement of a photosynthetic purple bacterium. Proceedings of the Royal Society of London B: Biological Sciences 273: 1943–1952.

[75] Cavalier-Smith T (2007) The chimaeric origin of mitochondria: photosynthetic cell enslavement, gene-transfer pressure, and compartmentation efficiency, Berlin, Heidelberg: Springer Berlin Heidelberg. pp. 161–199. doi: [10.1007/978-3-540-38502-8_8](http://dx.doi.org/10.1007/978-3-540-38502-8_8). http://­dx.doi.org/­10.1007/­978-3-540-38502-8_8.

[76] Woese CR (1977) Endosymbionts and mitochondrial origins. Journal of Molecular Evolution 10: 93–96.

[77] Cavalier-Smith T (2002) The phagotrophic origin of eukaryotes and phylogenetic classification of Protozoa. International Journal of Systematic and Evolutionary Microbiology 52: 297–354.

[78] Martin WF, Garg S, Zimorski V (2015) Endosymbiotic theories for eukaryote origin. Philosophical Transactions of the Royal Society of London B: Biological Sciences 370: 1–18.

[79] Martin W, Hoffmeister M, Rotte C, Henze K (2001) An overview of endosymbiotic models for the origins of eukaryotes, their ATP-producing organelles (mitochondria and hydrogenosomes), and their heterotrophic lifestyle. Biological Chemistry 382: 1521–1539.

[80] Martin W (1999) A briefly argued case that mitochondria and plastids are descendants of endosymbionts, but that the nuclear compartment is not. Proceedings of the Royal Society of London B: Biological Sciences 266: 1387–1395.

[81] Martin W, Koonin EV (2006) Introns and the origin of nucleus-cytosol compartmentalization. Nature 440: 41–45.

[82] Lane N, Martin W (2012) The origin of membrane bioenergetics. Cell 151: 1406–1416.

[83] Gould SB, Garg SG, Martin WF (2016) Bacterial vesicle secretion and the evolutionary origin of the eukaryotic endomembrane system. Trends in Microbiology 24: 525–534.

[84] Searcy DG (2006) Rapid hydrogen sulfide consumption by *Tetrahymena pyriformis* and its implications for the origin of mitochondria. European Journal of Protistology 42: 221–231.

[85] Searcy DG, Stein DB, Green GR (1978) Phylogenetic affinities between eukaryotic cells and a thermophilic mycoplasma. Biosystems 10: 19–28.

[86] Searcy DG (2014) Elemental sulfur reduction by eukaryotic cytoplasm consistent with an ancient sulfur symbiosis. Nitric Oxide 39, Supplement: S13.

[87] Margulis L, Chapman M, Guerrero R, Hall J (2006) The last eukaryotic common ancestor (LECA): Acquisition of cytoskeletal motility from aerotolerant spirochetes in the Proterozoic Eon. Proceedings of the National Academy of Sciences 103: 13080–13085.

[88] Margulis L (1970) Origin of eukaryotic cells: Evidence and research implications for a theory of the origin and evolution of microbial, plant, and animal cells on the Precambrian Earth. New Haven, CT: Yale University Press, 349 pp.

[89] Margulis L (1996) Archaeal-eubacterial mergers in the origin of Eukarya: phylogenetic classification of life. Proceedings of the National Academy of Sciences of the United States of America 93: 1071–1076.

[90] Hixon WG, Searcy DG (1993) Cytoskeleton in the archaebacterium *Thermoplasma acidophilum*? viscosity increase in soluble extracts. Biosystems 29: 151–160.

[91] Gray MW (2014) The pre-endosymbiont hypothesis: a new perspective on the origin and evolution of mitochondria. Cold Spring Harbor Perspectives in Biology 6.

[92] Mahler HR (1981) Mitochondrial evolution: organization and regulation of mitochondrial genes. Annals of the New York Academy of Sciences 361: 53–75.

[93] Mahler HR, Perlman PS, Hanson DK, Dhawale S (1981) Introns in mitochondria and their possible significance in evolution. In: Scudder GGE, Reveal JL, editors, Evolution Today. Pittsburg, Pennsylvania: Hunts Institute for Botanical Documentation, pp. 245–256.

[94] Yutin N, Wolf MY, Wolf YI, Koonin E (2009) The origins of phagocytosis and eukaryogenesis. Biology Direct 4.

[95] Jékely G (2007) Origin of phagotrophic eukaryotes as social cheaters in microbial biofilms. Biology Direct 2.

[96] Poole AM, Neumann N (2011) Reconciling an archaeal origin of eukaryotes with engulfment: a biologically plausible update of the Eocyte hypothesis. Research in Microbiology 162: 71–76.

[97] Andersson SGE, Kurland CG (1999) Origins of mitochondria and hydrogenosomes. Current Opinion in Microbiology 2: 535–541.

[98] Kurland CG, Andersson SGE (2000) Origin and evolution of the mitochondrial proteome. Microbiology and Molecular Biology Reviews 64: 786–820.

[99] Andersson GE, Karlberg O, Canbäck B, Kurland CG (2003) On the origin of mitochondria: a genomics perspective. Philosophical Transactions of the Royal Society of London B: Biological Sciences 358: 165–179.

[100] Lane N, Martin W (2010) The energetics of genome complexity. Nature 467: 929–934.

[101] Lane N (2011) Energetics and genetics across the prokaryote-eukaryote divide. Biology Direct 6: 1–31.

[102] Lane N (2014) Bioenergetic constraints on the evolution of complex life. Cold Spring Harbor Perspectives in Biology 6.

[103] Blackstone NW (2013) Why did eukaryotes evolve only once? Genetic and energetic aspects of conflict and conflict mediation. Philosophical Transactions of the Royal Society of London B: Biological Sciences 368: 1–7.

[104] Keeling PJ (2010) The endosymbiotic origin, diversification and fate of plastids. Philosophical Transactions of the Royal Society of London B: Biological Sciences 365: 729–748.

[105] Stairs CW, Leger MM, Roger AJ (2015) Diversity and origins of anaerobic metabolism in mitochondria and related organelles. Philosophical Transactions of the Royal Society of London B: Biological Sciences 370.

[106] Husnik F, Nikoh N, Koga R, Ross L, Duncan R, et al. (2013) Horizontal gene transfer from diverse bacteria to an insect genome enables a tripartite nested mealybug symbiosis. Cell 153: 1567–1578.

[107] von Dohlen CD, Kohler S, Alsop ST, McManus WR (2001) Mealybug β-proteobacterial endosymbionts contain γ-proteobacterial symbionts. Nature 412: 433–436.

[108] Wujek DE (1979) Intracellular bacteria in the blue-green alga *Pleurocapsa minor*. Transactions of the American Microscopical Society 98: 143–145.

[109] Thao ML, Gullan PJ, Baumann P (2002) Secondary (γ-proteobacteria) endosymbionts infect the primary (β-proteobacteria) endosymbionts of mealybugs multiple times and coevolve with their hosts. Applied and Environmental Microbiology 68: 3190–3197.

[110] Zimorski V, Ku C, Martin WF, Gould SB (2014) Endosymbiotic theory for organelle origins. Current Opinion in Microbiology 22: 38–48.

[111] Marin B, Nowack ECM, Melkonian M (2005) A plastid in the making: evidence for a second primary endosymbiosis. Protist 156: 425–432.

[112] Bodył A, Mackiewicz P, Gagat P (2012) Organelle evolution: *Paulinella* breaks a paradigm. Current Biology 22: R304–R306.

[113] McFadden GI (2014) Origin and evolution of plastids and photosynthesis in eukaryotes. Cold Spring Harbor Perspectives in Biology 6.

[114] Nakayama T, Kamikawa R, Tanifuji G, Kashiyama Y, Ohkouchi N, et al. (2014) Complete genome of a nonphotosynthetic cyanobacterium in a diatom reveals recent adaptations to an intracellular lifestyle. Proceedings of the National Academy of Sciences of the United States of America 111: 11407–11412.

[115] Yoon HS, Hackett JD, Ciniglia C, Pinto G, Bhattacharya D (2004) A molecular timeline for the origin of photosynthetic eukaryotes. Molecular Biology and Evolution 21: 809–818.

[116] Parfrey LW, Lahr DJG, Knoll AH, Katz LA (2011) Estimating the timing of early eukaryotic diversification with multigene molecular clocks. Proceedings of the National Academy of Sciences of the United States of America 108: 13624–13629.

[117] Keeling PJ (2013) The number, speed, and impact of plastid endosymbioses in eukaryotic evolution. Annual Review of Plant Biology 64: 583–607.

[118] Leliaert F, Smith DR, Moreau H, Herron MD, Verbruggen H, et al. (2012) Phylogeny and molecular evolution of the green algae. Critical Reviews in Plant Sciences 31: 1–46.

[119] López-García P, Eme L, Moreira D (2017) Symbiosis in eukaryotic evolution. Journal of Theoretical Biology .

[120] Davidov Y, Jurkevitch E (2009) Predation between prokaryotes and the origin of eukaryotes. BioEssays 31: 748–757.

[121] Sagan L (1967) On the origin of mitosing cells. Journal of Theoretical Biology 14: 225–274.

[122] de Duve C (1969) Evolution of the peroxisome. Annals of the New York Academy of Sciences 168: 369–381.

[123] Lyons TW, Reinhard CT, Planavsky NJ (2014) The rise of oxygen in Earth’s early ocean and atmosphere. Nature 506: 307–315.

[124] Anbar AD, Knoll AH (2002) Proterozoic ocean chemistry and evolution: a bioinorganic bridge? Science 297: 1137–1142.

[125] Johnston DT, Wolfe-Simon F, Pearson A, Knoll AH (2009) Anoxygenic photosynthesis modulated Proterozoic oxygen and sustained Earth’s middle age. Proceedings of the National Academy of Sciences of the United States of America 106: 16925–16929.

[126] Szathmáry E (2015) Toward major evolutionary transitions theory 2.0. Proceedings of the National Academy of Sciences of the United States of America 112: 10104–10111.

[127] Al Mamun M, Albergante L, Moreno A, Carrington JT, Blow JJ, et al. (2016) Inevitability and containment of replication errors for eukaryotic genome lengths spanning megabase to gigabase. Proceedings of the National Academy of Sciences 11: E5765–E5774.

[128] Nelson-Sathi S, Dagan T, Landan G, Janssen A, Steel M, et al. (2012) Acquisition of 1,000 eubacterial genes physiologically transformed a methanogen at the origin of Haloarchaea. Proceedings of the National Academy of Sciences of the United States of America 109: 20537–20542.

[129] Groussin M, Boussau B, Szöllősi G, Eme L, Gouy M, et al. (2015) Gene acquisitions from bacteria at the origins of major archaeal clades are vastly overestimated. Molecular Biology and Evolution 33: 305–310.

[130] Fenchel T, Bernard C (1993) Endosymbiotic purple non-sulphur bacteria in an anaerobic ciliated protozoon. FEMS Microbiology Letters 110: 21–25.

[131] Desmond E, Brochier-Armanet C, Forterre P, Gribaldo S (2011) On the last common ancestor and early evolution of eukaryotes: reconstructing the history of mitochondrial ribosomes. Research in Microbiology 162: 53–70.

[132] Lang BF (2014) Mitochondria and the origin of eukaryotes, Vienna: Springer, chapter 1. pp. 3–18. doi: [10.1007/978-3-7091-1303-5](http://dx.doi.org/10.1007/978-3-7091-1303-5). http://­dx.doi.org/­10.1007/­978-3-7091-1303-5.

[133] Gross J, Bhattacharya D (2009) Mitochondrial and plastid evolution in eukaryotes: an outsiders’ perspective. Nature Reviews Genetics 10: 495–505.

[134] Ettema TJG, Lindås AC, Bernander R (2011) An actin-based cytoskeleton in archaea. Molecular Microbiology 80: 1052–1061.

[135] Erickson HP (2007) Evolution of the cytoskeleton. BioEssays 29: 668–677.

[136] Yutin N, Koonin EV (2012) Archaeal origin of tubulin. Biology Direct 7: 1–9.

[137] Diekmann Y, Pereira-Leal JB (2013) Evolution of intracellular compartmentalization. Biochemical Journal 449: 319–331.

[138] Čuboňová L, Sandman K, Hallam SJ, DeLong EF, Reeve JN (2005) Histones in Crenarchaea. Journal of Bacteriology 187: 5482-5485.

[139] Čuboňová L, Katano M, Kanai T, Atomi H, Reeve JN, et al. (2012) An archaeal histone is required for transformation of *Thermococcus kodakarensis*. Journal of Bacteriology 194: 6864–6874.

[140] Gabaldón T (2014) A metabolic scenario for the evolutionary origin of peroxisomes from the endomembranous system. Cellular and Molecular Life Sciences 71: 2373–2376.

[141] Bolte K, Rensing SA, Maier UG (2015) The evolution of eukaryotic cells from the perspective of peroxisomes. BioEssays 37: 195–203.

[142] Poole AM, Gribaldo S (2014) Eukaryotic origins: How and when was the mitochondrion acquired? Cold Spring Harbor Perspectives in Biology 6.

[143] Schönheit P, Buckel W, Martin WF (2016) On the origin of heterotrophy. Trends in Microbiology 24: 12–25.

[144] Schlüter A, Ruiz-Trillo I, Pujol A (2011) Phylogenomic evidence for a myxococcal contribution to the mitochondrial fatty acid beta-oxidation. PLoS ONE 6: 1–9.

[145] Rochette NC, Brochier-Armanet C, Gouy M (2014) Phylogenomic test of the hypotheses for the evolutionary origin of eukaryotes. Molecular Biology and Evolution 31: 832-845.

[146] Soubannier P Vincent adn Rippstein, Kaufman BA, Shoubridge EA, McBride HM (2012) Reconstitution of mitochondria derived vesicle formation demonstrates selective enrichment of oxidized cargo. PLoS ONE 7: 1-9.

[147] Lombard J, López-García P, Moreira D (2012) The early evolution of lipid membranes and the three domains of life. Nature Reviews Microbiology 10: 507–515.

[148] Junglas B, Briegel A, Burghardt T, Walther P, Wirth R, et al. (2008) Ignicoccus hospitalis and *Nanoarchaeum equitans*: ultrastructure, cell–cell interaction, and 3d reconstruction from serial sections of freeze-substituted cells and by electron cryotomography. Archives of Microbiology 190: 395–408.

[149] Huber H, Küper U, Daxer S, Rachel R (2012) The unusual cell biology of the hyperthermophilic crenarchaeon *Ignicoccus hospitalis*. Antonie van Leeuwenhoek 102: 203–219.

[150] de Duve C (2007) The origin of eukaryotes: a reappraisal. Nature Reviews Genetics 8: 1471–0056.

[151] Wächtershäuser G (2003) From pre-cells to Eukarya - A tale of two lipids. Molecular Microbiology 47: 13–22.

[152] Koga Y (2011) Early evolution of membrane lipids: how did the lipid divide occur? Journal of Molecular Evolution 72: 274–282.

[153] Shimada H, Yamagishi A (2011) Stability of heterochiral hybrid membrane made of bacterial sn-G3P lipids and archaeal sn-G1P lipids. Biochemistry 50: 4114-4120.

[154] Pogozheva ID, Tristram-Nagle S, Mosberg HI, Lomize AL (2013) Structural adaptations of proteins to different biological membranes. Biochimica et Biophysica Acta (BBA) - Biomembranes 1828: 2592–2608.

[155] Williams TA, Foster PG, Cox CJ, Embley TM (2013) An archaeal origin of eukaryotes supports only two primary domains of life. Nature 504: 231–236.

[156] Lombard J, Moreira D (2011) Origins and early evolution of the mevalonate pathway of isoprenoid biosynthesis in the three domains of life. Molecular Biology and Evolution 28: 87-99.

[157] Boucher Y, Kamekura M, Doolittle WF (2004) Origins and evolution of isoprenoid lipid biosynthesis in archaea. Molecular Microbiology 52: 515–527.

[158] Madigan MT, Martinko JM, Bender KS, Buckley DH, Stahl DA (2014) Brock biology of microorganisms. Benjamin Cummings, 14 edition, 1136 pp.

[159] Cavalier-Smith T (2006) Cell evolution and Earth history: stasis and revolution. Philosophical Transactions of the Royal Society of London B: Biological Sciences 361: 969–1006.

[160] Jékely G (2007) Origin of eukaryotic endomembranes: a critical evaluation of different model scenarios, New York, NY: Springer New York, volume 607 of *Advances in Experimental Medicine and Biology*. pp. 38–51. doi: [10.1007/978-0-387-74021-8_3](http://dx.doi.org/10.1007/978-0-387-74021-8_3). http://­dx.doi.org/­10.1007/­978-0-387-74021-8_3.

[161] Maynard Smith J, Szathmáry E (1999) The origins of life: from the birth of life to the origin of language. USA: Oxford University Press, 180 pp.

[162] Cavalier-Smith T (2013) Symbiogenesis: mechanisms, evolutionary consequences, and systematic implications. Annual Review of Ecology, Evolution, and Systematics 44: 145–172.

[163] Fenchel T, Bernard C (1993) A purple protist. Nature 362: 300–300.

[164] Gabaldón T, Huynen MA (2003) Reconstruction of the proto-mitochondrial metabolism. Science 301: 609.

[165] van der Giezen M, Slotboom DJ, Horner DS, Dyal PL, Harding M, et al. (2002) Conserved properties of hydrogenosomal and mitochondrial ADP/ATP carriers: a common origin for both organelles. The EMBO Journal 21: 572–579.

[166] Horner DS, Hirt RP, Embley TM (1999) A single eubacterial origin of eukaryotic pyruvate: ferredoxin oxidoreductase genes: implications for the evolution of anaerobic eukaryotes. Molecular Biology and Evolution 16: 1280–1291.

[167] Cox CJ, Foster PG, Hirt RP, Harris SR, Embley TM (2008) The archaebacterial origin of eukaryotes. Proceedings of the National Academy of Sciences of the United States of America 105: 20356–20361.

[168] Kelly S, Wickstead B, Gull K (2011) Archaeal phylogenomics provides evidence in support of a methanogenic origin of the Archaea and a thaumarchaeal origin for the eukaryotes. Proceedings of the Royal Society of London B: Biological Sciences 278: 1009–1018.

[169] Williams TA, Foster PG, Nye TMW, Cox CJ, Embley TM (2012) A congruent phylogenomic signal places eukaryotes within the Archaea. Proceedings of the Royal Society of London B: Biological Sciences 279: 4870–4879.

[170] Searcy DG (1987) Phylogenetic and phenotypic relationships between the eukaryotic nucleocytoplasm and thermophilic archaebacteria. Annals of the New York Academy of Sciences 503: 168–179.

[171] Fitzpatrick DA, Creevey CJ, McInerney JO (2006) Genome phylogenies indicate a meaningful α-proteobacterial phylogeny and support a grouping of the mitochondria with the rickettsiales. Molecular Biology and Evolution 23: 74–85.

[172] Canbäck B, Andersson SGE, Kurland CG (2002) The global phylogeny of glycolytic enzymes. Proceedings of the National Academy of Sciences 99: 6097–6102.

[173] Cavalier-Smith T (2002) The neomuran origin of archaebacteria, the negibacterial root of the universal tree and bacterial megaclassification. International Journal of Systematic and Evolutionary Microbiology 52: 7–76.

[174] Sassera D, Lo N, Epis S, D’Auria G, Montagna M, et al. (2011) Phylogenomic evidence for the presence of a flagellum and cbb3 oxidase in the free-living mitochondrial ancestor. Molecular Biology and Evolution 28: 3285–3296.

[175] Wang Z, Wu M (2014) Phylogenomic reconstruction indicates mitochondrial ancestor was an energy parasite. PLoS ONE 9: 1-11.

[176] Sousa FL, Neukirchen S, Allen JF, Lane N, Martin WF (2016) Lokiarchaeon is hydrogen dependent. Nature Microbiology 1.

[177] Lill R, Kispal G (2000) Maturation of cellular fe–s proteins: an essential function of mitochondria. Trends in Biochemical Sciences 25: 352–356.

[178] Katinka MD, Duprat S, Cornillot E, Metenier G, Thomarat F, et al. (2001) Genome sequence and gene compaction of the eukaryote parasite encephalitozoon cuniculi. Nature 414: 450–453.

[179] Embley TM, van der Giezen M, Horner D, Dyal P, Bell S, et al. (2003) Hydrogenosomes, mitochondria and early eukaryotic evolution. IUBMB Life 55: 387–395.

[180] Kannan S, Rogozin IB, Koonin EV (2014) MitoCOGs: clusters of orthologous genes from mitochondria and implications for the evolution of eukaryotes. BMC Evolutionary Biology 14: 1–16.

[181] Gabaldón T, Pittis AA (2015) Origin and evolution of metabolic sub-cellular compartmentalization in eukaryotes. Biochimie 119: 262–268.

[182] Dolezal P, Likic V, Tachezy J, Lithgow T (2006) Evolution of the molecular machines for protein import into mitochondria. Science 313: 314–318.

[183] van Niftrik LA, Fuerst JA, Damsté JSS, Kuenen JG, Jetten MSM, et al. (2004) The anammoxosome: an intracytoplasmic compartment in anammox bacteria. FEMS Microbiology Letters 233: 7–13.

[184] Drews G, Golecki JR (1995) Structure, molecular organization, and biosynthesis of membranes of purple bacteria, Dordrecht: Springer Netherlands. pp. 231–257. doi: [10.1007/0-306-47954-0_12](http://dx.doi.org/10.1007/0-306-47954-0_12). http://­dx.doi.org/­10.1007/­0-306-47954-0_12.

[185] Hanson RS, Hanson TE (1996) Methanotrophic bacteria. Microbiological Reviews 60: 439-71.

[186] Muñoz-Gómez S, Slamovits C, Dacks J, Baier K, Spencer K, et al. (2015) Ancient homology of the mitochondrial contact site and cristae organizing system points to an endosymbiotic origin of mitochondrial cristae. Current Biology 25: 1489–1495.

[187] Karlberg O, Canbäck B, Kurland CG, Andersson SGE (2000) The dual origin of the yeast mitochondrial proteome. Yeast 17: 170–187.

[188] John P, Whatley FR (1975) *Paracoccus denitrificans* and the evolutionary origin of the mitochondrion. Nature 254: 495–498.

[189] Margulis L (1981) Symbiosis in cell evolution: life and its environment on the early earth. San Francisco: Freeman.

[190] McInerney JO, Martin WF, Koonin EV, Allen JF, Galperin MY, et al. (2011) Planctomycetes and eukaryotes: A case of analogy not homology. BioEssays 33: 810–817.

[191] Maynard Smith J, Szathmáry E (1995) The major transitions in evolution. Oxford: Freeman & Co., 360 pp.

[192] Jeon KW (1972) Development of cellular dependence on infective organisms: micrurgical studies in amoebas. Science 176: 1122–1123.

[193] Jeon KW (1995) The large, free-living amoebae: wonderful cells for biological studies. Journal of Eukaryotic Microbiology 42: 1–7.

[194] Cavalier-Smith T (1983) Endosymbiotic origin of the mitochondrial envelope, de Gruyter Berlin. pp. 265–279.

[195] Schleiff E, Soll J (2005) Membrane protein insertion: mixing eukaryotic and prokaryotic concepts. EMBO Reports 6: 1023–1027.

[196] Mileykovskaya E, Dowhan W (2009) Cardiolipin membrane domains in prokaryotes and eukaryotes. Biochimica et Biophysica Acta 1788: 2084–2091.

[197] Zeth K (2010) Structure and evolution of mitochondrial outer membrane proteins of β-barrel topology. Biochimica et Biophysica Acta (BBA) - Bioenergetics 1797: 1292–1299.

[198] Koumandou VL, Wickstead B, Ginger ML, van der Giezen M, Dacks JB, et al. (2013) Molecular paleontology and complexity in the last eukaryotic common ancestor. Critical Reviews in Biochemistry and Molecular Biology 48: 373–396.

[199] Keeling PJ (2004) Diversity and evolutionary history of plastids and their hosts. American Journal of Botany 91: 1481-1493.

[200] Jékely G (2014) Origin and evolution of the self-organizing cytoskeleton in the network of eukaryotic organelles. Cold Spring Harbor Perspectives in Biology 6.

[201] van den Ent F, Amos LA, Löwe J (2001) Prokaryotic origin of the actin cytoskeleton. Nature 413: 39–44.

[202] Szwedziak P, Wang Q, Freund SMV, Löwe J (2012) FtsA forms actin-like protofilaments. The EMBO Journal 31: 2249–2260.

[203] Searcy DG, Hixon WG (1991) Cytoskeletal origins in sulfur-metabolizing archaebacteria. Biosystems 25: 1–11.

[204] Lindås AC, Bernander R (2013) The cell cycle of archaea. Nature Reviews Microbiology 11: 627–638.

[205] Gould SB (2016) Infection and the first eukaryotes. Science 352: 1065–1065.

[206] Koonin EV (2015) Origin of eukaryotes from within archaea, archaeal eukaryome and bursts of gene gain: eukaryogenesis just made easier? Philosophical Transactions of the Royal Society of London B: Biological Sciences 370.

[207] Williams TA, Embley TM (2015) Changing ideas about eukaryotic origins. Philosophical Transactions of the Royal Society of London B: Biological Sciences 370.

[208] Jékely G (2008) Eukaryotic membranes and cytoskeleton: origins and evolution, volume 607. Springer Science & Business Media, 145 pp.

[209] Garg SG, Martin WF (2016) Mitochondria, the cell cycle, and the origin of sex via a syncytial eukaryote common ancestor. Genome Biology and Evolution 8: 1950-1970.

[210] Guerrero R, Pedrós-Alió C, Esteve I, Mas J, Chase D, et al. (1986) Predatory prokaryotes: Predation and primary consumption evolved in bacteria. Proceedings of the National Academy of Sciences 83: 2138-2142.

[211] Moebius N, Üzüm Z, Dijksterhuis J, Lackner G, Hertweck C (2014) Active invasion of bacteria into living fungal cells. eLife 3: e03007.

[212] Booth A, Doolittle WF (2015) Eukaryogenesis, how special really? Proceedings of the National Academy of Sciences 112: 10278-10285.

[213] DiSalvo S, Haselkorn TS, Bashir U, Jimenez D, Brock DA, et al. (2015) *Burkholderia* bacteria infectiously induce the proto-farming symbiosis of *Dictyostelium* amoeba and food bacteria. Proceedings of the National Academy of Sciences 112: E5029–E5037.

[214] Margulis L, Dolan MF, Whiteside JH (2005) "Imperfections and oddities" in the origin of the nucleus. Paleobiology 31: 175–191.

[215] Lynch M, Marinov GK (2016) Reply to Lane and Martin: Mitochondria do not boost the bioenergetic capacity of eukaryotic cells. Proceedings of the National Academy of Sciences 113: E667-E668.

[216] Lane N, Martin WF (2016) Mitochondria, complexity, and evolutionary deficit spending. Proceedings of the National Academy of Sciences 113: E666.

[217] Cavalier-Smith T (1983) A 6-kingdom classification and a unified phylogeny. Endocytobiology II : 1027–1034.

[218] Baum DA (2015) A comparison of autogenous theories for the origin of eukaryotic cells. American Journal of Botany 102: 1954–1965.

[219] Bell PJL (2001) Viral eukaryogenesis: was the ancestor of the nucleus a complex DNA virus? Journal of Molecular Evolution 53: 251–256.
